# Expression of Structural Flavonoid Biosynthesis Genes in Dark-Blue and White Myrtle Berries (*Myrtus communis* L.)

**DOI:** 10.3390/plants10020316

**Published:** 2021-02-06

**Authors:** Silvia Medda, Maria Teresa Sanchez-Ballesta, Irene Romero, Leonarda Dessena, Maurizio Mulas

**Affiliations:** 1 Department of Agricultural Science, University of Sassari, Via De Nicola 9, 07100 Sassari, Italy; 30044461@studenti.uniss.it (S.M.); lalladessena@uniss.it (L.D.); 2 Instituto de Ciencia y Tecnologia de Alimentos y Nutrición (ICTAN-CSIC), Jose Antonio Novais, 10, 28040 Madrid, Spain; mballesta@ictan.csic.es (M.T.S.-B.); irene.romero@ictan.csic.es (I.R.); 3 Centre for Conservation and Evaluation of Plant Biodiversity, University of Sassari, Via De Nicola 9, 07100 Sassari, Italy

**Keywords:** myrtle berry, pigmentation, anthocyanins, flavonoids gene expression, RT-qPCR

## Abstract

Within the myrtle (*Myrtus communis* L.) species, different genotypes may produce dark-blue berries or white berries depending on the peel color upon ripening. One dark-blue cultivar and one white myrtle cultivar were used to study the molecular mechanisms underlying flavonoid biosynthesis. The relative expression levels of common (*PAL*, *CHS*, *CHI*, *DFR* and *LDOX*) and specific (*FLS*, *ANR*, *LAR* and *UFGT*) flavonoid genes were analyzed during fruit development by means of quantitative real-time polymerase chain reaction (RT-qPCR). Moreover, the anthocyanin content was determined, and it showed an increase with the ripening of the berries of the dark-blue cultivar. The results showed an increased transcript abundance of *PAL*, *CHI*, *DFR*, *LDOX* and *UFGT* gene expression in the dark-blue cultivar compared to the white one, as well as a strong positive correlation between the changes in gene expression and anthocyanin accumulation. The transcript levels of *UFGT* showed sharp increases at 150 and 180 days after full blooming (DAF) in the dark-blue cultivar, which corresponded with anthocyanin accumulation. However, ripening seemed to modulate the expression of genes implicated in flavonols (i.e., *FLS*) and flavan-3-ols (i.e., *LAR* and *ANR*) in different manners. However, whereas *FLS* transcript accumulation increased at the end of the ripening period in the dark-blue cultivar, *LAR* and *ANR* gene expression decreased in both cultivars.

## 1. Introduction

Phenolic compounds are secondary metabolites. They include several molecules characterized by an aromatic ring bound to one or more hydroxyl groups. Some examples of these compounds are phenolic acids, flavonoids, condensed tannins, and lignin. They are involved in plant defense against biotic and abiotic stresses [1]. Anthocyanins are members of this group and are water-soluble pigments belonging to a class of flavonoids that widely contribute to the blue, red, and purple coloring of several fruits, vegetables, and flowers. Their content and composition of plants may be influenced by several ecological factors, such as altitude, temperature, light exposure, and growing management [2,3,4,5,6]. The postharvest treatment and storage conditions of plant-derived food can affect the quantity and quality of these compounds [7,8]. Depending on the intensity, controlled abiotic stresses in plants can induce transcription factors that are involved in secondary metabolite biosynthesis and can affect the enzyme activity of the genes involved in that pathway [9]. Several studies have focused on two important functions of these pigments. Firstly, their use as natural colorants in the food industry to replace synthetic ones was the object of some research [10,11]. On the other hand, the implications of anthocyanins for human health have promoted numerous studies about the antioxidant, anticancer, anti-inflammatory, and antimutagenic activities of the different compounds, as well as their positive effects against cardiovascular disease and diabetes [12,13,14,15].

Fruit are potentially suitable as natural anthocyanin sources; of these, myrtle (*Myrtus communis* L.) is a spontaneous aromatic shrub that is widely spread in the Mediterranean region and belongs to the Myrtaceae family. It has been used and appreciated in folk medicine. The economic and scientific interest in this plant has also grown due to its high content of polyphenolic compounds. Several scientific studies have focused on its antioxidant properties and their correlation with the anthocyanin content in the fruit, leaves, and extracts of myrtle [16,17,18,19]. The organoleptic properties [20] and the shelf life [21] of the sweet myrtle liquor that is produced in Sardinia (Italy) are influenced by the amount of anthocyanins in the hydroalcoholic extract. Because of the high industrial demand for biomass and with the aims of protecting the natural population and providing raw material, a domestication and cultivation program for this species has been promoted [22]. The species has high phenotypic variability and may be classified according to the color of the ripe berries, such as dark red, dark blue (*M. communis* var. *melanocarpa* DC), or white (*M. communis* var. *leucocarpa* DC) [22]. Previous comparative studies between dark and white myrtle genotypes showed a higher total phenolic content and a higher anthocyanin content in extracts of pigmented berries compared to white berries [23,24]. However, most studies concerning myrtle have been carried out on dark berry genotypes, such as those mentioned above.

The precursors of most flavonoids are malonyl–Coenzyme–A (CoA)—derived from carbohydrate metabolism—and phenylalanine—derived from phenylpropanoid metabolism. Phenylalanine ammonia–lyase (PAL) catalyzes the transformation of phenylalanine into trans-cinnamic acid. The first enzyme involved in flavonoid biosynthesis is CHS (chalcone synthase), which catalyzes the condensation of three molecules of malonyl–CoA with a molecule of *p* coumaroyl–CoA to form naringenin chalcone. This product is rapidly isomerized by chalcone isomerase (CHI) into the flavanone naringenin. Through hydroxylation of naringenin by flavanone 3-hydroxylase (F3H), dihydrokaempferol is formed. Dihydrokaempferol can be subsequently hydroxylated at the 3′ position, by flavonoid 3′-hydroxylase (F3′H), forming dihydroquercetin. However, if hydroxylation occurs at the 3′ and 5′ positions of naringenin through the flavonoid 3′,5′-hydroxylase enzyme (F3′5′H), dihydromyricetin is formed. Dihydroquercetin is involved in the production of cyanidin-based anthocyanin, while dihydromyricetin is the precursor of delphinidin-based anthocyanin. Then, the dihydroflavonol-4-reductase enzyme (DFR) drives the reduction of dihydroflavanol into leucoanthocyanidins (flavan-3,4-diol), the precursors of anthocyanins. Alternatively, dihydroflavanols can be transformed into kaempferol, quercetin, and myricetin flavanols by the flavanol synthase enzyme (FLS). Leucoanthocyanidins are converted into anthocyanidins by leucoanthocyanidin dioxygenase (LDOX); subsequently, leucoanthocyanidins are stabilized and accumulate in the cellular vacuoles. This last step is catalyzed by enzyme UDP-glucose: the flavonoid 3-*O*-glucosyltransferase (UFGT) transfers the glucose moiety from UDP-glucose to the free hydroxyl group at the 3′ position of the heterocyclic ring of anthocyanidin. In contrast to anthocyanidin production, proanthocyanidins—which are also called condensed tannins and are oligomers or polymers of flavan-3-ol units—can be produced. Two enzymes—leucoanthocyanidin reductase (LAR) and anthocyanidin reductase (ANR)—can produce the flavan-3-ol monomers required for the formation of proanthocyanidin polymers [25,26].

Although there have been several studies [16,17,18,19] on the phenolic profile of myrtle leaves and berries, as well as their biological properties—e.g., their antioxidant activity—to the best of our knowledge, there is no information available about the biosynthesis at the gene expression level. Currently, only studies of the phenylalanine ammonia lyase (PAL) enzymatic activity during the development of berries and leaves in dark-blue and white myrtle cultivars have been carried out [27].

Previous studies of other species [28,29,30] have shown that the expression of most of the anthocyanin pathway genes is higher in dark fruits compared to white ones. In spite of the variable expression of genes involved in the flavonoid pathway in different species and tissues, a certain correlation with anthocyanin accumulation has been found [30]. For example, sweet red cherries show an up-regulated gene expression during ripening that is higher than that of yellow cultivars [30]. *PAL*, *CHS*, *CHI*, *DFR* and *LDOX* gene expression was up-regulated in the red “Yunhong-1” pear variety compared to the white “Zaobaimi” variety [31]. Likewise, Lin et al. [32] suggested that the pink–white mutant of Chinese bayberry was due to a reduction in the expression of key biosynthesis genes in the later stages of development. Moreover, their expression could be influenced by abiotic factors, such as temperature [33], light, [34] and hormones [35].

In this work, two myrtle cultivars—“Giovanna” with dark-blue fruit and “Grazia” with white fruit fruit—were identified according to the domestication process and the varietal selection of myrtle plants and were analyzed [22]. In a previous work [36], the metabolic profile of the “Giovanna” cultivar (namely in the work RUM 13) was analyzed. The study showed that the most abundant anthocyanins in ripe berries are delphinidin 3-*O*-glucoside, cyanidin 3*-O*-glucoside, petunidin 3-*O*-glucoside, and malvidin 3-*O*-glucoside (486.6, 144.6, 356.9, and 937.2 mg/100 g dry weight (DW), respectively). The detected flavanols with the highest amounts were myricetin 3-galacatoside (183.1 mg/100 g dry weight) and myricetin 3-rhamnoside (135.57 mg/100 g dry weight). Regarding the quantitative composition of the phenolic compounds found in a previous study, at ripening, the “Giovanna” cultivar showed a lower content of total phenolic compounds than the “Grazia” cultivar (37 and 46 mg gallic acid equivalent (GAE)/g DW for “Giovanna” and “Grazia”), as well as a lower content of total tannins (0.33 mg catechin equivalent (CE)/g DW for “Giovanna” and 2.73 mg CE/g DW for “Grazia”) [27]. From the perspective of genetic diversity, both cultivars have been evaluated using ISSR (Inter simple sequence repeat) and AFLP (Amplified fragment length polymorphism) molecular marker analyses. The results revealed a clear separation between the cultivars according to the berry pigmentation [37,38]. Indeed, all analyzed unpigmented cultivars showed common genome regions that were not found in pigmented cultivars.

The aim of this work was to provide preliminary information about the molecular mechanisms that lead to myrtle berry pigmentation by studying the expression of common and specific genes of flavonoid biosynthesis during the physiological ripening of the fruit at different stages of development as well as and how these changes correlate with total anthocyanin and phenolic content.

## 2. Results

### 2.1. Isolation and Characterization of Common and Specific Flavonoid Biosynthetic Genes

Nine partial cDNA clones, *PAL* (GenBank accession no. MT745992), *CHS* (GenBank accession no. MT745993), *CHI* (GenBank accession no. MT745994), *DFR* (GenBank accession no. MT745995), *LDOX* (GenBank accession no. MT745996), *FLS* (GenBank accession no. MT745997), *LAR* (GenBank accession no. MT745998), *ANR* (GenBank accession no. MT745999) and *UFGT* (GenBank accession no. MT746000) were isolated from *Myrtus communis* L. For this experiment, peel samples were used; the peel is the fruit tissue with the highest anthocyanin accumulation. The different fragments were isolated via real-time polymerase chain reaction (RT-PCR) using primers corresponding to the sequences from *Eucalyptus*. The *Eucalyptus* genome was chosen because all gene sequences from *Myrtus communis* were available in the databases, and both species belong to the same family—Myrtaceae. The sequence analysis indicated that the different partial clones showed more than 90% identity compared with the corresponding genes from *Eucalyptus grandis*. Moreover, the partial fragments presented an identity higher than 70% compared with the corresponding genes from *Vitis vinifera* (Table 1). Likewise, an identity higher than 60% was found when the different partial PCR fragments and genes from *Malus* × *domestica* were compared, as well as *Arabidopsis thaliana*, with the exception of *FLS* from *M. domestica*, which showed an identity of 55%. No *LAR* genes were found in *Arabidopsis*, [39] so only comparisons with the *LAR* genes of *E. grandis, V. vinifera,* and *M. domestica* are shown.

We would like to highlight that a detailed BLAST search showed that the *PAL* fragment from myrtle presented 95% identity when compared with five of the nine *PAL* genes from *Eucalyptus*. These five *PAL* genes (XM_010069015, XM_010069014, XM_010069016, XM_010069013 and XM_010069017) were located in chromosome 5 and presented a 95% identity. Likewise, in the case of *UFGT*, the BLAST search also showed a 91.04% identity compared with the two *UFGTs* (XM_010064907 and XM_010064884) identified in *Eucalyptus*. The alignments of the partial nucleotide sequences obtained from the purified PCR fragments and, likewise, the translated amplicon-amino acid sequences from *M. communis* with the corresponding nucleotide and full-length amino acid sequences from *E. grandis, V. vinifera, A. thaliana,* and *M. domestica* (Table 1), are presented in the Supplementary Materials (Figures S1–S18).

### 2.2. Expression of Common and Specific Flavonoid Biosynthetic Genes

**Figure 1:**
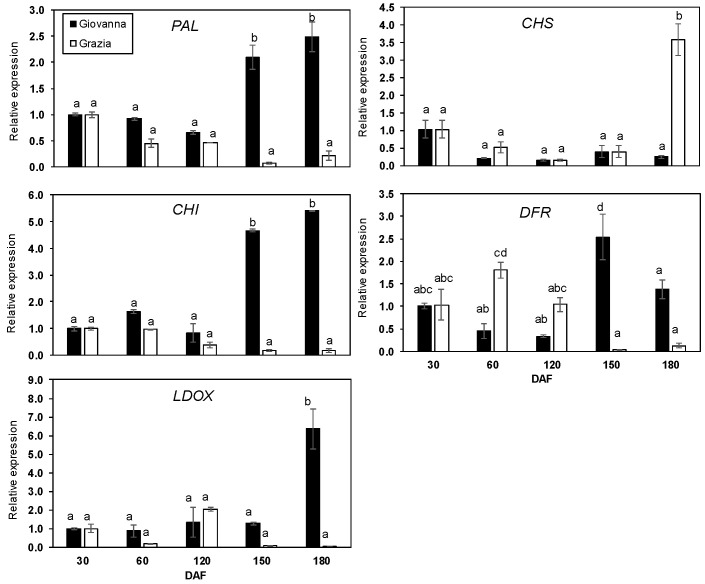
Expression analysis of *PAL*, *CHS*, *CHI*, *DFR*, and *LDOX* during five stages of “Giovanna” (dark-blue) and “Grazia” (white) myrtle fruit development. Transcript levels of each gene were assessed by RT-qPCR and normalized using actin as a reference gene. The results were calculated relative to a calibrator sample (30 days after full blooming (DAF) from dark-blue samples) using the formula 2^−ΔΔCt^. Error bars represent standard error of three biological replicates. Every replication consisted of 40 berries. Different letters represent statistically significant differences with Tukey’s B-test (*p* < 0.05). Tukey’s B-test was carried out by comparing all means calculated at different DAFs for both cultivars.

**Figure 2:**
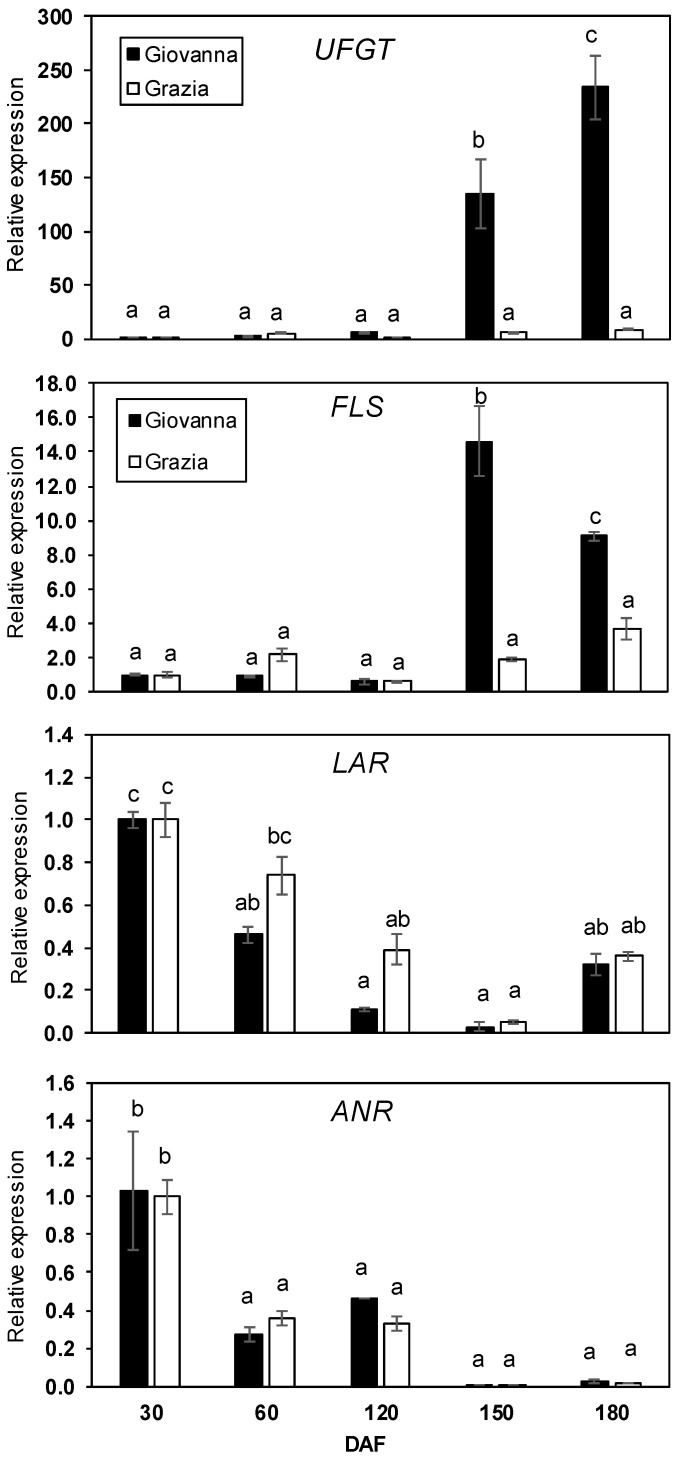
Expression analysis of *UFGT, FLS*, *LAR,* and *ANR* during five stages of “Giovanna” (dark-blue) and “Grazia” (white) myrtle fruit development. Transcript levels of each gene were assessed by RT-qPCR and normalized using actin as reference gene. The results were calculated relative to a calibrator sample (30 DAF from dark-blue samples) using the formula 2^−ΔΔCt^. Error bars represent standard error of three biological replicates. Every replication consisted of 40 berries. Different letters represent statistically significant differences with Tukey’s B-test (*p* < 0.05). Tukey’s B-test was carried out by comparing all means calculated at different DAFs for both cultivars.

The relative levels of five common genes of the flavonoid biosynthetic pathway (*PAL*, *CHS*, *CHI*, *DFR* and *LDOX*)—a specific anthocyanin gene (*UFGT*) and genes coding for enzymes implicated in the synthesis of flavonols (FLS) and flavan-3-ols (LAR and ANR)—were analyzed over the five developmental stages of dark-blue and white myrtle fruit (Figure 1 and Figure 2). The relative levels of the analyzed genes showed two distinct patterns of expression between the “Giovanna” and “Grazia” cultivars. In the dark-blue “Giovanna” fruit, the accumulation of *PAL*, *CHI*, and *UFGT* transcripts showed a sharp increase during the last stages of berry maturation at 150 and 180 DAF (days after full blooming) (Figure 1 and Figure 2). Moreover, *DFR* and *FLS* showed peak expression at 150 DAF, while *LDOX* showed a peak at 180 DAF. It is important to note that *UFGT* transcript expression increased by more than 200 times at 180 DAF (Figure 2). However, the *CHS* transcript did not vary throughout the development (Figure 1). Regarding the white “Grazia” cultivar, it is interesting to note that only the increase in *CHS* and the decrease in *DFR* expression showed significant differences. Thus, the relative change in *CHS* was approximately 2.5-fold at 180 DAF (Figure 1), whereas, in the case of *DFR*, (Figure 1) it was two-fold at 60 DAF and decreased with ripening. Finally, the *LAR* and *ANR* genes, which are implicated in flavan-3-ol biosynthesis, presented a similar pattern of accumulation in both cultivars. A significant decrease in the expression of both genes throughout the development period was observed. In the dark-blue fruit, the *LAR* gene decreased by about 60% at ripening compared to at 30 DAF, whereas *ANS* decreased by about 98% in ripe fruit compared to at 30 DAF (Figure 2).

### 2.3. Total Anthocyanin and Phenolic Content

**Figure 3:**
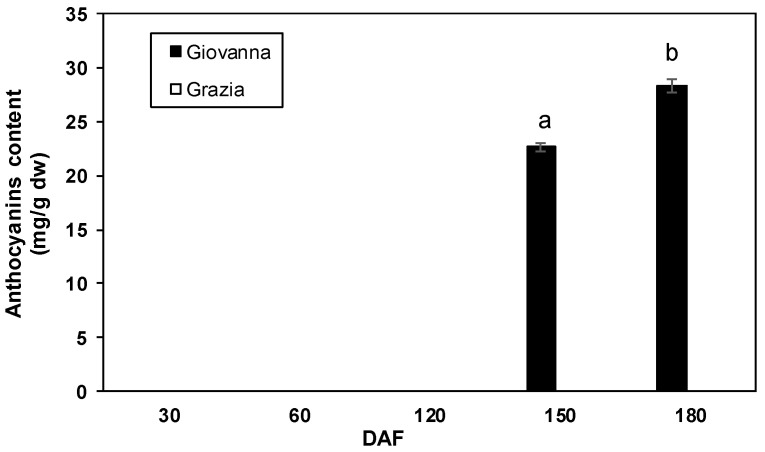
Total anthocyanin content at different development stages of “Giovanna” (dark-blue) and “Grazia” (white) fruit. Error bars represent the standard error of the mean of three biological replicates. Every replication consisted of 40 berries. Different letters represent statistically significant differences with Tukey’s B-test (*p* < 0.05). Tukey’s B-test was carried out by comparing all means calculated at different DAFs for both cultivars.

**Figure 4:**
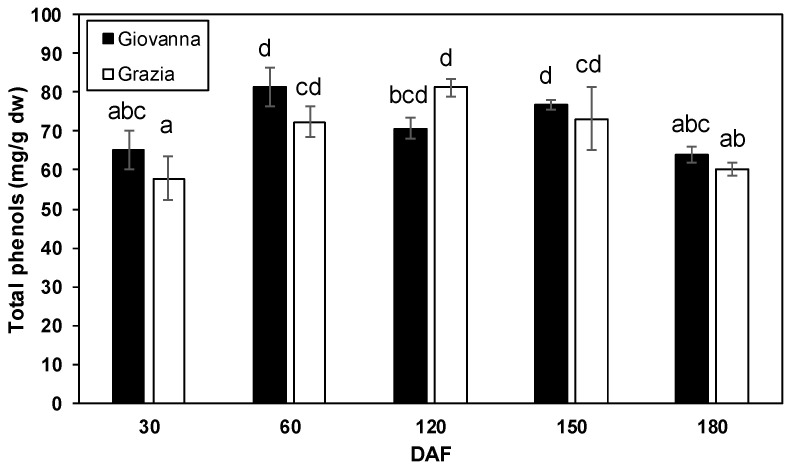
Total phenolic content at different development stages of “Giovanna” (dark-blue) and “Grazia” (white) fruit. Error bars represent the standard error of the mean of three biological replicates. Every replication consisted of 40 berries. Different letters represent statistically significant differences with Tukey’s B-test (*p* < 0.05). Tukey’s B-test was carried out by comparing all means calculated at different DAFs for both cultivars.

The total anthocyanin content detected in both genotypes reflected the phenotypical differences between them. Thus, total anthocyanin was only recorded in the “Giovanna” fruit at 120 and 150 DAF with 22.61 and 28.24 mg cyanidin 3-glucoside equivalent/g dry matter, respectively. However, anthocyanins were not detected in the “Grazia” samples (Figure 3). Moreover, strong positive correlations were noted between the *PAL*, *CHI*, *DFR*, *LDOX,* and *UFGT* transcript accumulation and the total anthocyanin content (*p* < 0.01) in the dark-blue “Giovanna” fruit (Table 2). Regarding the total phenolic content, no significant differences between dark-blue and white myrtle fruit were found. Two significant peaks were recorded for “Giovanna” at 60 and 150 DAF while one peak was found for “Grazia” at 120 DAF (Figure 4). Thereafter, the levels were maintained until 180 DAF, and they decreased to levels similar to those observed at 30 DAF. However, though the maximum levels were recorded at 60 DAF in the dark-blue fruit, they were recorded at 120 DAF in white ones. Moreover, it is important to note that no correlations were found between gene expression and total phenolic content (data not shown).

## 3. Discussion

This study is the first investigation of flavonoid biosynthesis in two myrtle cultivars from a molecular perspective. The use of dark-blue and white genotypes allowed us to understand the differences between the expression levels of key genes that are responsible for the different colorations of ripe berries in flavonoid synthesis. Partial fragments of *PAL*, *CHI*, *CHS*, *DFR*, *FLS*, *ANR*, *LAR*, *ANS*, and *UFGT* (Figure 1 and Figure 2) were isolated from myrtle fruit for the first time, and the relative levels of expression were measured via quantitative RT-PCR (RT-qPCR). We would like to highlight that, although the partial *PAL* and *UFGT* fragments from myrtle presented a high identity with five and two Eucalyptus paralogs, respectively, our results showed that the other partial fragments that were isolated only possessed a similar identity to one gene from Eucalyptus. Moreover, it is important to note that some of these key genes presented multiple copies with altered expression during fruit ripening [40,41,42,43,44]. However, in the present study, due to the lack of information about the myrtle genome, we cannot determine the number of copies at the present time.

The dark-blue fruit of the “Giovanna” cultivar showed a content of anthocyanins ranging from 22.61 to 28.24 mg of cyanidin 3-glucoside equivalent/g of dry matter during ripening. Another study on myrtle berries derived from the same domestication process as the cultivars analyzed in this study showed a content of 220.12 mg/100 g fresh weight after 150 DAF [20]. Moreover, it is also possible to find traces of anthocyanins in white myrtle fruit. The macerate obtained through the infusion of white berries in alcohol showed a high content of gallic and ellagic acids and flavonols, especially myricetin-3-*O*-rhamnoside, while malvidin-3-*O*-glucoside was present in traces [45].

**Figure 5:**
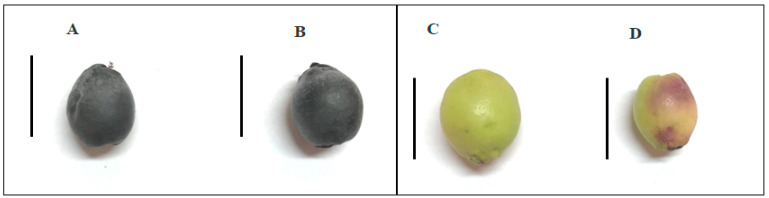
(**A**) “Giovanna” berry cultivar at 150 DAF; (**B**) “Giovanna” berry cultivar at 180 DAF; (**C**) “Grazia” berry cultivar at 150 DAF; (**D**) “Grazia” berry cultivar at 180 DAF. Scale bar equals 1 cm.

With regard to the biological differences between the cultivars, all of the myrtle cultivars are characterized by a high plant vigor with strong green biomass production, and this characteristic is linked to high fruit production. However, the mean plant production per year of “Grazia” is 4.5 kg of berries compared to 2.5 kg for “Giovanna”. Moreover, the ripening time of “Grazia” is about 30 days before that of “Giovanna”. As shown in Figure 5 at 180 DAF, the white “Grazia” cultivar during 180 DAF starts to change its peel color. However, the possible anthocyanin accumulation with overripening was under the sensitivity thresholds of the applied method.

In this study, an increase in *PAL* expression was observed from 30 to 180 DAF in the dark-blue “Giovanna” fruit (Figure 1), which was correlated with the anthocyanin content during the ripening stages (150 and 180 DAF) (Table 2). In some plant species, *PAL* expression increased with ripening and was correlated with fruit pigmentation [46]. In other species, such as apples, this correlation was not found [47]; thus, it is assumed that *PAL* is not a key enzyme for anthocyanin formation in these species. Consequently, these authors suggested that this close correlation does not always occur because anthocyanins may originate not only from phenylalanine, but also from other precursors if they are present in high amounts. However, our results suggest that, in myrtle berries, phenylalanine could be the main precursor of anthocyanin biosynthesis. In myrtle, *CHS* gene expression only increased at the last ripening stage in the white fruit (Figure 1). This trend was also observed in unpigmented strawberry cultivars (*Fragaria chiloensis* L.), as reported by Salvatierra et al. [48]. Furthermore, Ju et al. [47] and Kondo et al. [49] reported that *CHS* in apples was not involved in anthocyanin biosynthesis, as its activity remained constant throughout the entire development stage, regardless of anthocyanin synthesis. Goto-Yamamoto et al. [42] showed that the mRNA of *CHS3* accumulated mainly in the grape berry skin of red cultivars during coloration, while *CHS1* and *CHS2* accumulated in the leaves and berry skin of both white and red cultivars. Moreover, Ageorges et al. [43] showed that, among the three *CHS* identified in grapes, only *CHS3* was strongly associated with color. The fact that CHS acts in the first step of the flavonoid biosynthetic pathway for several compounds, including anthocyanins, suggests that, in myrtle fruit, the biosynthesis of other phenolic compounds could be involved, such as proanthocyanidins, as previously reported by Downey et al. [50]. Contrarily to *CHS* gene expression, the accumulation of *CHI* showed a peak at the end of the ripening stage (180 DAF) in the dark-blue cultivar, which was correlated with the content of total anthocyanins. In this case, *CHI* might be a rate-limiting enzyme in anthocyanin formation, in contrast to the findings of other studies [51,52]. *DFR* gene expression levels also increased with the ripening stage in the dark-blue myrtle cultivar, showing a peak at 150 DAF. Moreover, these changes were positively correlated with anthocyanin accumulation. The white “Grazia” cultivar showed an opposite trend, with higher levels at the early stages of development (especially at 60 DAF) compared to maturation. The *DFR* gene expression results are in agreement with those of several previous studies. Roy et al. [46] found a higher expression level in the ripe red strawberry genotype than in the white genotype. In addition, in the red fruit of *Fragaria pentaphylla*, a strong correlation between *DFR* gene expression and anthocyanin content was found [53]. Furthermore, the expression of *DFR* gradually increased with ripening in *Prunus persica* and was correlated with the accumulation of anthocyanins [54]. In pomegranate, *DFR* showed higher expression levels in the dark and red genotypes than in the green and white ones, and was correlated with anthocyanin accumulation [55]. On other hand, in *Ginkgo biloba*, an isoform of DFR related to anthocyanin accumulation was found, as well as one that was not related, which could be involved in tannic acid synthesis [56]. Regarding the *LDOX* and *UFGT* genes, we found that their expression increased with ripening and was strictly correlated with anthocyanin accumulation in the “Giovanna” cultivar. According to these results, in strawberry, an up-regulation of *LDOX* and *UFGT* in the red receptacle at the ripening stage was also observed [46,48]. By contrast, whereas *UFGT* expression and anthocyanin amount were strictly correlated in the red Tsugaru apple cultivar, *LDOX* did not seem to directly regulate anthocyanin formation [49]. In the dark-blue myrtle berry, *UFGT* expression recorded the sharpest increase out of all the studied genes. In contrast, in the white “Grazia” samples, *UFGT* was detected at very low expression levels. It is known that variations in color intensity can be attributed to differences in the expression of structural or regulatory genes. In pink–white Chinese bayberry fruit, *LDOX* and *UFGT* expression was 70% and 90% lower than that of the red fruit at the ripening stage [32]. Previous studies reported *UFGT* as the key gene of anthocyanin biosynthesis in sweet cherry [30], mangosteen [57], and litchi [58]. Moreover, Boss et al. [51] found *UFGT* expression exclusively in grape tissue, where anthocyanins were also detected, but only in the in red variety and not in the white one. In this sense, white grapes have arisen through mutations in two MYB transcription factors that specifically control *UFGT* expression [59,60]. Moreover, Takos et al. [61] showed that the transcript levels of multiple anthocyanin pathway genes, including *MdUFGT1* and *MdMYB1*, were much lower in non-red-skinned apples than in red-skinned ones, concluding that the expression level of this transcription factor could be the genetic basis for apple skin color. By contrast, Meng et al. [62] indicated that *UFGT* is important for anthocyanin accumulation in apple skins of different colors, especially in non-red apples. These authors showed that, whereas *MdUFGT2* was up-regulated only in non-red cultivars, *MdUFGT4* was up-regulated only in the red-skinned cultivar. Our results seem to indicate that *UFGT* could be a key gene responsible for the different pigmentations in the two myrtle genotypes. In this sense, in the future, it will be interesting to identify *MYBs* or other *UFGT* regulatory elements in order to determine if they are essential regulatory elements during myrtle ripening.

*UFGT* showed a higher level of expression in blood oranges than in common oranges, and its expression was correlated with *CHS* and *LDOX* expression [63]. In this sense, our results indicated a strong positive correlation between *UFGT* gene expression and *PAL* (r = 0.886, *p* < 0.01), *CHI* (r = 0.929, *p* < 0.01), *DFR* (r = 0.538, *p* < 0.01), and *LDOX* (r = 0.886, *p* < 0.01) expression. *FLS*, a key gene for flavonol accumulation, showed higher accumulation in the dark-blue myrtle cultivar, though no significant differences were observed in the white cultivar during the fruit development stages. These results are in acccordance with those of Wang et al. [64], who observed increased levels of the *FLS* transcript in red-fleshed crabapples in comparison to white ones. These authors indicated that the different colorings of fruits are derived from the different expression profiles of structural genes belonging to a network related to flavonoid biosynthesis. In fact, *FLS* was higher in the ripening stage than in the early development stage in the pigmented cultivar in citrus peels [65]. Moreover, our results seem to indicate that *PAL* expression could play a role in flavonol accumulation, showing a positive correlation with *FLS* accumulation (r = 0.778, *p* < 0.01). In this sense, Morales et al. [66] showed a positive correlation between *PAL* expression and the accumulation of four flavonols in *Betula pendula* leaves. The *ANR* and *LAR* genes, which are involved in proanthocyanidin biosynthesis, showed higher expression levels in the first stage (30 DAF) of development than in the ripening stage (180 DAF) for both myrtle cultivars; a similar pattern was observed in mulberry, in which *ANR* and *LAR* gene expression was highest in young fruitlets and decreased as the fruit developed. Moreover, the expression of these genes decreased with ripening in strawberries [48]. In addition to MYBs, members of several protein superfamilies mediate the transcriptional regulation of the flavonoid biosynthetic pathway, such as basic helix–loop–helix (bHLH) transcription factors and conserved WD40 repeat (WDR) proteins [67]. Therefore, an in-depth study of these regulatory elements may be of great interest for understanding the different expression profiles of the flavonoid genes of both cultivars.

Regardless of the total phenolic content, the maximum content was reached in the “Giovanna” berry at 60 DAF and in the “Grazia” one month later (at 120 DAF); it then decreased in both cultivars upon ripening. In the dark-blue myrtle cultivars, a decrease in total phenolic content and an increase in anthocyanin content were found during ripening [20]. Messaoud and Boussaid [24] indicated that the total phenolic content was higher in red myrtle berries than in white ones; however, in our work, no significant differences between the two cultivars were found. These differences could be explained by the fact that the total phenol content in the fruit depends on several factors, such as genetic factors, the location, and the year of sampling. Contrary to the anthocyanin content, no correlation was found between the total phenolic content and the expression levels analyzed in either cultivar, indicating that it is the anthocyanin trend that seems to regulate gene expression in myrtle during development and ripening. Moreover, the absence of a correlation between total phenolic content and gene expression levels may be due to the sensitivity of the assay used for total phenolic quantification. In fact, the presence of reducing sugars or other reducing compounds in the solution—analyzed via the Folin–Ciocalteu method—may, in part, lead to an overestimation of the total phenolic content [68]. By contrast, Parra-Palma et al. [69] found a positive correlation between total phenolic content and *FLS* and *ANR* expression levels in strawberries, suggesting that flavonols and condensed tannins are the major phenolic compounds in the first stages of ripening.

## 4. Materials and Methods

### 4.1. Plant Material

Two cultivars of myrtle (*Myrtus communis* L.) with dark-blue berries (“Giovanna”) and white berries (“Grazia”) were analyzed. Fifteen plants of each cultivar were grown in a collection field belonging to the University of Sassari in Fenosu (Oristano, Central–West Sardinia, Italy; 39°53′ North, 8°37′ East; 12 m a.s.l). Berry sampling was carried out over five stages of development, which were defined according to the number of days after flowering (DAF) in the season from July to December 2018 at 30, 60, 120, 150, and 180 DAF. The last stages, 150 and 180 DAF, can be considered overripening stages, particular for the “Grazia” cultivar, which showed earlier ripening with respect to the “Giovanna” cultivar (Figure 5).

The fruit was harvested from 20 plants of each cultivar, with a total of 120 berries sampled for each stage of development. Seeds and core pulp were removed from the berries, and peels with about 1 mm of pulp were ground using a pestle and mortar with liquid nitrogen and stored at −80 °C until analysis.

### 4.2. Total RNA Extraction, cDNA Synthesis, and RT-PCR

Total RNA was extracted from three biological replicates for each sample (and, for each biological replication, two technical replications were carried out) using the protocol indicated by [70] with some modifications. The extracted RNA quantification was conducted using a Nanodrop 8000 spectrophotometer (Thermo Fisher Scientific, Waltham, MA, USA). RNA integrity was verified through agarose gel electrophoresis. Total RNA was treated with Ambion^®^ DNA-free DNase Treatment (Life Technologies, Carlsbad, CA, USA) to remove possible DNA contamination. Then, cDNA was synthesized from 1 µg of treated RNA using an NZY First-Strand cDNA Synthesis kit (NZYTech™, Lisbon, Portugal) according to the manufacturer’s instructions. Gene-specific primer pairs for RT-PCR (Table 3) were designed with the Primer 3 software [71] using the gene sequences from *Eucaliptus* extracted from the NCBI (National Center for Biotechnology Information) database as templates. The efficiency of the reaction to establish the most suitable template and primer concentrations was calculated according to [72].

As a template, a pool of cDNA synthetized using RNA extracted from the pigmented myrtle fruit at 120, 150, and 180 DAF was used. Sanger sequencing at Secugen (Madrid, Spain) confirmed the PCR fragments. BLASTN was used to perform similarity searches in the NCBI databases.

### 4.3. Quantitative Real-Time RT-PCR (RT-qPCR)

The relative expression of all studied genes was assayed with RT-qPCR using Supreme NZYTaq II 2× Green Master (NZYTech™, Lisbon, Portugal), as described by [72]. The reaction mix was composed as follows: 11.25 µL SYBR green, 4.5 µL primer forward and primer reverse (3 µm), 4.5 µL water and 4.5 µL cDNA. The amplification program consisted of initial denaturation at 95 °C for 5 min, followed by 35 denaturation cycles at 94 °C for 30 s, pairing for 30 s, extension at 72 °C for 30 s, and final extension at 72 °C for 5 min. Amplifications were run in a 96-well plate iCycler iQ thermal cycler (Bio-Rad, Hercules, CA, USA), and quantification was performed with the iCycler iQTM software (Real Time Detection System Software, version 2.0, Bio-Rad Laboratories, Hercules, CA, USA). Each gene was evaluated at least in two independent runs. The previously indicated gene-specific primers (Table 1) were used. The actin gene was used as the internal reference gene for normalizing the transcript profiles. Gene-specific primers were designed for *ACTIN-1* from *Eucalyptus* (Table 3). Relative expression levels were estimated with the 2^−ΔΔCt^ method and were denoted as the fold difference from the expression present at the calibrator sample (30 DAF from dark-blue samples). The specificity of the products was validated with dissociation curve analysis and with agarose gel; the sequences were confirmed via Sanger sequencing at Secugen (Madrid, Spain).

### 4.4. Determination of Total Anthocyanin and Total Phenolic Content

For these determinations, 600 mg of fruit sample (peel with 1 mm of adherent pulp) pulverized in liquid nitrogen was extracted with 10 mL of acidified ethanol (0.1% hydrochloric acid) and placed in the dark overnight at room temperature. The extracts were filtered with Whatman filter paper and stored at −20 °C until the chemical analysis (within one week after sampling). The same extract was used for both anthocyanin and total phenolic content determination. Three biological replicates were analyzed for each sample.

The total anthocyanin content was quantified using the spectrophotometric method proposed by [73] with some modifications according to the expected concentration of anthocyanins. Then, 0.5 mL of acidified ethanol extract was added to 6.5 mL potassium chloride buffer at pH 1, while the other 0.5 mL of extract was added to 6.5 mL of sodium acetate buffer (pH 4.5). After 30 min, the absorbance (A) of the two mixtures was read at both 510 and 700 nm and was calculated as (A) = (A510–A700 nm) pH 1.0–(A510–A700 nm) pH 4.5 [74].

The content of anthocyanins was calculated according to the following equation [74]:

Anthocyanins = (A × MW × DF × 1000)/(ε × l),(1)

A = Absorbance
MW = Molecular weight
DF = Dilution factor
ε = Molar extinction coefficient, l × mol^−1^ × cm^−1^
l = Pathlength (1 cm)

Anthocyanin content was expressed as mg of cyanidin-3-glucoside equivalent/g of dry matter (DW). The results were compared with a calibration curve that was obtained using a range of concentrations of 0.05–0.30 mg/mL.

Total phenolic content was determined with the Folin–Ciocalteu colorimetric method [75]. For this, 0.5 mL of ethanolic acidified extract diluted with 35 mL of deionized water was added to 2.5 mL of Folin–Ciocalteu reagent. After 3 min of incubation, 5 mL of sodium carbonate solution (20% in water) was added to the mixture. The solution was heated at 70 °C for 20 min and deionized water was added to bring the solution to a final volume of 50 mL. The absorbance was read at 750 nm using a CARY 50 Scan Uv-Vis VARIAN (Amsterdam, The Netherlands) spectrophotometer. The calibration curve was obtained using a range of gallic acid between 0.05 and 0.5 mg/mL. The results were expressed as mg of gallic acid equivalent (GAE)/g DW.

### 4.5. Statistical Analysis

After analysis for normality and homogeneity, an analysis of variance (ANOVA) was performed using the SSPS statistical software with a split-plot design in order to evaluate the influence of the main variables: cultivar (A) and DAF (B). Tukey’s B-test (*p* ≤ 0.05) was applied for mean separation at the AxB level of the dependent variables (*PAL*, *CHS*, *CHI*, *DFR*, *FLS*, *ANR*, *LAR*, *LDOX*, and *UFGT* relative expression).

The correlations between the relative levels of structural gene expression, total anthocyanin content, and total phenolic content were evaluated with the Pearson coefficient at 99% significance using the R Studio software (version: 3.4.4; PBC, Boston, MA, USA).

## 5. Conclusions

In this preliminary study, we analyzed, for the first time, the expression of the structural genes involved in flavonoid biosynthesis in myrtle fruit. The overall results indicated that the differences in coloration between the dark-blue “Giovanna” cultivar and the white “Grazia” fruit might be due to the over-regulation of some structural genes in dark-blue fruit, particularly the *LDOX* and *UFGT* genes. Moreover, this first molecular approach showed significant positive correlations between the common and specific anthocyanin genes and the total anthocyanin content, whereas no correlations were found with total phenolic content. Further work, with the aim of elucidating the molecular mechanisms underlying the loss of anthocyanins in the white fruit cultivar and the eventual role of the transcription factors involved in flavonoid gene modulation, could initiate an interesting research line with regard to myrtle fruit.

## Figures and Tables

**Table 1:** Percentage of identity for partial sequences from myrtle with other species.

	Description	Identity	Accession
*PAL*	PREDICTED: *E. grandis* phenylalanine ammonia-lyase	95.00%	XM_010069013
	PREDICTED: *E. grandis* phenylalanine ammonia-lyase	95.00%	XM_010069014
	PREDICTED: *E. grandis* phenylalanine ammonia-lyase	95.00%	XM_010069015
	PREDICTED: *E. grandis* phenylalanine ammonia-lyase	94.17%	XM_010069016
	PREDICTED: *E. grandis* phenylalanine ammonia-lyase	94.17%	XM_010069017
	*V. vinifera* phenylalanine ammonia-lyase 1 (*PAL1*)	80.00%	KU162973
	PREDICTED: *M. domestica* phenylalanine ammonia-lyase 1-like	79.80%	XM_008357397
	*A. thaliana* phenylalanine ammonia-lyase 4 (*PAL4*)	76.50%	NM_111869
*CHS*	PREDICTED: *E. grandis* chalcone synthase 3	97.00%	XM_010030619
	*V. vinifera* chalcone synthase	85.10%	JF808008
	*A. thaliana* chalcone synthase family protein	82.80%	DQ062406
	*M. domestica MdCHS* chalcone synthase	80.60%	AB074485
*CHI*	PREDICTED: *E. grandis* chalcone--flavonone isomerase 3	92.30%	XM_010069312
	PREDICTED: *M. domestica* chalcone--flavonone isomerase 3	77.30%	XM_008371146
	PREDICTED: *V. vinifera* chalcone--flavonone isomerase 3	76.60%	XM_002280122
	*A. thaliana* chalcone-flavanone isomerase family protein (*CHIL*)	69.00%	NM_120609
*DFR*	PREDICTED: *E. grandis* dihydroflavonol-4-reductase	90.03%	XM_010062668
	*V. vinifera* dihydroflavonol 4-reductase	74.60%	AY780886
	*M. domestica* dihydroflavonol 4-reductase	71.00%	AY227728
	*A. thaliana* dihydroflavonol 4-reductase	67.40%	AK221622
*FLS*	PREDICTED: *E. grandis* flavonol synthase/flavanone 3-hydroxylase	93.80%	XM_010065055
	*V. vinifera FLS5* gene flavonol synthase	70.90%	AB213566
	*A. thaliana* flavonol synthase gene	60.30%	U84258
	*M. domestica* flavonol synthase	55.81%	AY965343
*LAR*	PREDICTED: *E. grandis* leucoanthocyanidin reductase	93.80%	XM_010045996
	*M. domestica* leucoanthocyanidin reductase 1	72.90%	DQ139836
	*V. vinifera* leucoanthocyanidin reductase 1 (*LAR1*)	69.80%	MK726357
*ANR*	PREDICTED: *E. grandis* anthocyanidin reductase	93.10%	XM_010054161
	*V. vinifera* anthocyanidin reductase (*ANR*)	78.10%	NM_001280956
	*M. domestica* anthocyanidin reductase (*ANR2a*)	76.90%	JN035300
	*A. thaliana* NAD(P)-binding Rossmann-fold superfamily protein (*BAN*)	67.50%	NM_104854
*LDOX*	PREDICTED: *E. grandis* leucoanthocyanidin dioxygenase	94.70%	XM_010055063
	*V. vinifera LDOX* leucoanthocyanidin dioxygenase	85.10%	X75966
	*M. domestica* leucoanthocyanidin dioxygenase-like	83.50%	NM_001328948
	*A. thaliana* leucoanthocyanidin dioxygenase (*LDOX)*	78.20%	NM_001036623
*UFGT*	PREDICTED: *E. grandis* anthocyanidin 3-*O*-glucosyltransferase 2	91.90%	XM_010064884
	PREDICTED: *E. grandis* anthocyanidin 3-*O*-glucosyltransferase 2	91.90%	XM_010064907
	*V. vinifera ItUFGT2* UDP-glucose:flavonoid 3-*O*-glucosyltransferase	73.70%	AB047093
	*A. thaliana* UDP glucose:flavonoid 3-*O*-glucosyltransferase-like protein	70.60%	AY072325
	*M. domestica* UDP glucose:flavonoid 3-*O*-glucosyl transferase (*UFGT1*)	60.20%	AF117267

**Table 2:** Pearson correlations between total anthocyanin content and gene expression levels in dark-blue fruit of “Giovanna” cultivar (significance: ** *p* < 0.01).

	*PAL*	*CHS*	*CHI*	*DFR*	*LDOX*	*UFGT*
Total anthocyanin content	0.854 **	−0.208	0.892 **	0.688 **	0.679 **	0.93 **

**Table 3:** Sequence of primers used for RT-qPCR analysis.

Gene	Forward Primer 5′–3′	Reverse Primer 5′–3′	Length Sequence (bp)
*ACT*	AGATGACCCAGATTATGTTTGAGACCTTC	ACCATCACCAGAATCCAACACAATACC	122
*PAL*	CAACCCTGTGACCAACCATG	TTCTCTTCCAGGTGCCTCAG	174
*CHS*	AGTCTTCTGCTCCACCTCTG	GATCTCAGAGCAGACGACGA	199
*CHI*	GCCACAGATGATGCCTTCTT	CTCTTCCTCCTCCTCCTCGT	200
*DFR*	CGCGAATTTGCTCAGGAAGA	AGCCCTTTCTCTCTGCATGT	183
*FLS*	TACTGGTCCCGAACGATGTC	AACACTGCCCATGACATTCG	193
*LDOX*	AGGTTGGAGAAGGAAGTCGG	AGGATCTCAATGGTGTCCCC	245
*UFGT*	CCAGAAGAGGACATCGAGCT	GCCGAGAGTCTGCCTGATAT	237
*LAR*	TGACATCGGGAAGTTCACCA	TGATGATGACACGAGGGAGG	155
*ANR*	GCCAAAGCGAAGACAGTGAA	TTTCCTCCGCGAATTTCCAC	200

## Supplementary Materials

The following are available online at ’https://www.mdpi.com/2223-7747/10/2/316/s1, Figures S1, S3, S5, S7, S9, S11, S13, S15, S17. Alignment of the partial nucleotide sequence of *PAL*, *CHS*, *CHI*, *DFR*, *LDOX*, *UFGT*, *FLS*, *LAR*, *ANR*, respectively, from *M. communis* and the full-length sequences from *E. grandis, V. vinifera*, *M. domestica* and *A. thaliana*. Identical nucleotides among the sequences are in a black background, whereas those that are similar among the nucleotide sequences are in grey background. The dashes indicate gaps introduced to improve alignment, Figures S2, S4, S6, S8, S10, S12, S14, S16, S18. Alignment of the translated amplicon-amino acid sequence from *M. communis* and the full-length *PAL*, *CHS*, *CHI*, *DFR*, *LDOX*, *UFGT*, *FLS*, *LAR*, *ANR*, respectively, sequences from *E. grandis, V. vinifera*, *M. domestica* and *A. thaliana*. Identical amino acids are shaded in black, and similar amino acids are shaded in grey. The remaining residues are in a white background.
