# Supplementary material for: Expression of Structural Flavonoid Biosynthesis Genes in Dark-Blue and White Myrtle Berries (Myrtus communis L.)
Source: Plants (Basel). 2021 Feb 6;10(2):316. doi: 10.3390/plants10020316 (PMC7915511; doi:10.3390/plants10020316)
Supplement: Supplementary file 1 [file plants-10-00316-s001.pdf]

Figure S1. Alignment of the partial nucleotide sequence of *PAL* from *M. communis* and the full-length sequences from *E. grandis, V. vinifera, M. domestica* and *A. thaliana*. Identical nucleotides among the sequences are in a black background, whereas those that are similar among the nucleotide sequences are in grey background. The dashes indicate gaps introduced to improve alignment.

```
XM_010069013Euc     1 -------------------------ATGGACATGGACACGGCAGCGTGCAACGGCAAGGG
XM_010069017Euc     1 -------------------------------ATGGACACGGCAGCGTGCAACGGCAAGGG
XM_010069014Euc     1 -------------------------ATGGACATGGACACGGCAGCGTGCAACGGCGAGGG
XM_010069015Euc     1 -------------------------ATGGACATGGACACGGCAGCGTGCAACGGCAAGGG
XM_010069016Euc     1 -------------------------ATGGACATGGACACGGCAGCGTGCAACGGCAAGGG
Myrtus              1 ------------------------------------------------------------
KU162973Vitis       1 ------------------ATGGAATTCTCACACCACAACGGCAACGGAAACGGAAAGGTG
XM_008357397Mal     1 ATGGAGGCGAAAACCATCACGCAAAATGGTCAAAACGGCCACCACCAGAACGGAGTGTCG
NM_111869Arabid     1 -------------------------------------------ATGGAGCTATGCAATCA

XM_010069013Euc    36 CTCCCTCGGGCTCTGCGTGGGGGCAGGTGGCGACCCGCTCAATTGGGGCGCCGCCGCCGC
XM_010069017Euc    30 CTGGCTCGGGCTCTGCTCGGGGGCAGGCGGTGACCCGCTCAACTGGGGCGCGGCCGCCGC
XM_010069014Euc    36 CTCCCTCGGGCTCTGCGCGGGGGCAGGAGGTGACCCACTCAACTGGGGCGCGGCTGCCGC
XM_010069015Euc    36 CTCCCTTGGGCTCTGCGCGGGGGCAGGCGGTGACCCACTCAACTGGGGCGCGGCTGCCGC
XM_010069016Euc    36 CTCCCTCGGGCTCTGCGCGGGGGCAGGCGGTGACCCGCTCAACTGGGGCGTGGCCGCCGC
Myrtus              1 ------------------------------------------------------------
KU162973Vitis      43 GACGCCAATGGCTTCTGCATGAA-GGCCGCGGATCCCCTCTACTGGGCGGCGGCGGCGGA
XM_008357397Mal    61 GAGTCCCAG---CTGTGCATTAA-AAA---GGACCCATTGAACTGGGGTCTAGTGGCTGA
NM_111869Arabid    18 AAACAATCACATCACCGCCGTCTCGGGC---GATCCGTTGAACTGGAACGCGACGGCCGA

XM_010069013Euc    96 GGCCCTCACGGGGAGCCACCTCGATGAGGTGAAGCGGATGGTCGAGGAGTCTCGGCGACC
XM_010069017Euc    90 GGCCCTCACGGGGAGCCACCTCGATGAGGTGAAGCGGATGGTTGAGGAGTCCCGGCGGCC
XM_010069014Euc    96 GGCCCTCACGGGGAGCCACCTCGATGAGGTGAAAAGGATGGTCGAGGAGTCCCGGCGGCC
XM_010069015Euc    96 GGCCCTCACGGGGAGCCACCTCGATGAGGTGAAAAGGATGGTCGAGGAGTCCCGGCGGCC
XM_010069016Euc    96 GGCCCTCACGGGGAGCCACCTCGATGAGGTGAAAAGGATGGTCGAGGAGTCCCGGCGGCC
Myrtus              1 ------------------------------------------------------------
KU162973Vitis     102 GACTCTGAAGGGGAGCCATTTGGAGGAGGTGAAGAGAATGGTGGCGGACTTCAGAAAACC
XM_008357397Mal   114 TTCACTAAAAGGGAGCCACTTGGATGAAGTGAAGCGTATGGTGGCCGAGTACAGAAAGCC
NM_111869Arabid    75 AGCTTTGAAAGGGAGCCACCTGGATGAGGTGAAACGAATGGTGAAAGAGTATAGGAAAGA

XM_010069013Euc   156 TGTGGTCCGCCTCGGCGGTGAGACGCTGACCATCGCACAGGTGGCGGCCATCGC---GGG
XM_010069017Euc   150 CGTGGTCCGCCTCGGCGGTGAGACGCTGACCATCGCACAGGTGGCGGCCATCGC---TGG
XM_010069014Euc   156 TGTGGTCCGCCTCGGCGGTGAGACGCTGACCATCGCACAGGTGGCGGCCATCGC---AGG
XM_010069015Euc   156 TGTGGTCCGCCTCGGCGGTGAGACGCTGACCATCGCACAGGTGGCGGCCATCGC---GGG
XM_010069016Euc   156 TGTGGTCCGCCTCGGCGGTGAGACGCTGACCATCGCACAGGTGGCGGCCATCGC---GGG
Myrtus              1 ------------------------------------------------------------
KU162973Vitis     162 GGTGGTGAGGCTTGGGGGTGAGAGCCTCAACATCGCACAGGTGGCGGCGGTGGCGGCCAC
XM_008357397Mal   174 GGTGGTGAAGCTCGGCGGAGAGAGTCTCACCATTTCCCAAGTTGCAGCCATAGC---CAC
NM_111869Arabid   135 GGCGGTGAAGTTAGGAGGTGAGACTTTGACGATTGGTCAAGTAGCCGCCGTGGC---TAG

XM_010069013Euc   213 GCGCGAGGCCGGCATGGCGGTCGAGCTGTCGGAGGCGGCCCGGGCGGGGGTCAAGGCCAG
XM_010069017Euc   207 GCAGGAGGCCGGTGCGGCGGTCGAGCTGTTGGAGGGGGCCCGGGCGGCGGTCAAGGCCAG
XM_010069014Euc   213 GCGGGAGGCCGGCGCAGCGGTCGAGCTGTCGGAGGCGGCCCGGGCGGCAGTCAAGGCCAG
XM_010069015Euc   213 GCGGGAGGCCGGCGCGGCGGTCGAGCTGTCGGAGGCGGCCCGGGCGGCAGTCAAGGCCAG
XM_010069016Euc   213 GCGGGAGGCTGGCATGGCGGTCGAGCTGTCGGAGGCGGCCCGGGCGGCAGTCAAGGCCAG
Myrtus              1 ------------------------------------------------------------
KU162973Vitis     222 CGGAAGCGACGGGGTGACAGTGGAACTGTCGGAGTCGGCGAGGGCCGGGGTGCAGGCCAG
XM_008357397Mal   231 TCATGACACCGGGGTCAAGGTTGAGCTGTCTGAGTCGGCTAGGGCTGGGGTCAAGGCCAG
NM_111869Arabid   192 AGGAGGAGGAGGATCTACGGTGGAGCTAGCGGAGGAGGCTCGTGCCGGAGTCAAGGCGAG

XM_010069013Euc   273 CAGCGACTGGGTGATGGAGAGCATGAGCAAGGGCACGGACAGCTACGGGGTCACCACTGG
XM_010069017Euc   267 CAGCGACTGGGTGATGGAGAGCATGAGCAACGGCACGGACAGCTACGGGATCACCACCGG
XM_010069014Euc   273 CAGCGACTGGGTGATGGAGAGCATGAGAAACGGCACGGACAGCTACGGGGTCACCACCGG
XM_010069015Euc   273 CAGCGACTGGGTGATGGAGAGCATGAGAAACGGCACGGACAGCTACGGGGTCACCACCGG
XM_010069016Euc   273 CAGCGACTGGGTCATGGAGAGCATGAGCAACGGCACGGACAGCTACGGGGTCACCACCGG
Myrtus              1 ------------------------------------------------------------
KU162973Vitis     282 CAGCAACTGGGTGATGGAGAGCATGAACCAAGGCACCGACAGTTATGGGGTGACTACCGG
XM_008357397Mal   291 CAGTGATTGGGTCATGGATAGCATGGGTAAGGGGACTGACAGCTACGGTGTCACCACCGG
NM_111869Arabid   252 TAGCGAATGGGTGATGGAGAGCATGAACCGAGGAACGGACAGTTATGGAGTTACCACAGG
```

```
XM_010069013Euc  333 CTTCGGCGCCACCTCGCATAGGCGGACCAAGCAAGGTGGTGCTCTGCAGAGGGAGCTCAT
XM_010069017Euc  327 CTTCGGCGCCACCTCGCATAGGCGGACCAAGCAAGGTGGTGCCCTGCAGATGGAGCTCAT
XM_010069014Euc  333 CTTCGGCGCCACCTCGCATAGGCGGACCAAGCAAGGTGGTGCTCTGCAGACGGAGCTCAT
XM_010069015Euc  333 CTTCGGCGCCACCTCGCATAGGCGGACCAAGCAAGGTGGTGCTCTGCAGACGGAGCTCAT
XM_010069016Euc  333 CTTCGGCGCCACCTCGCATAGGCGGACCAAGCAAGGTGGTGCTCTGCAGACGGAGCTCAT
Myrtus             1 ------------------------------------------------------------
KU162973Vitis    342 GTTCGGTGCGACTTCCCATAGAAGAACCAAACAAGGAAATGCTCTTCAGAAAGAACTGAT
XM_008357397Mal  351 GTTTGGTGCAACCTCCCACCGGAGAACAAACAAAGGAGCTGCCCTTCAAAAGGAGCTAAT
NM_111869Arabid  312 GTTTGGTGCAACTTCCCATAGAAGAACCAAACAAGGCGGTGCACTTCAAAATGAGCTTAT

XM_010069013Euc  393 AAGGTTCTTGAACGCCGGCATATTCGGCAATGGGAC-TGAGTCGTGCCACACGC--TGCC
XM_010069017Euc  387 AAGGTTCTTGAATGCCGGCATATTCGGCAATGGGAC-TGAGTCGTGCCACACTC--TGCC
XM_010069014Euc  393 AAGGTTCTTGAACGCCGGCATATTCGGCAACGGGAC-CGAGTCGTGCCACATGC--TGCC
XM_010069015Euc  393 AAGGTTCTTGAACGCCGGCATATTCGGCAACGGGAC-CGAGTCGTGCCACGTGC--TGCC
XM_010069016Euc  393 AAGGTTCTTAAACGCCGGCATATTCGGCAACGGGAC-CGAGTCGTGCCACGTGC--TGCC
Myrtus             1 ------------------------------------------------------------
KU162973Vitis    402 CAGATTCTTGAACGCTGGAATATTTGGGAAGGGAAC-AGAGTCATGCCACACAT--TGCC
XM_008357397Mal  411 TAGATTCTTGAACGCTGGAGTGTTTGGAAGTGCTAC-AGAATCAGGCCACACAC--TGCC
NM_111869Arabid  372 TAGGTTCTTGAATGCCGGAATATTTGGCCCCGGCGCCGGGGACACGTCACACACGTTGCC

XM_010069013Euc  450 CCACTCGGCGACCCGGGCTGCGATGCTCGTGAGGATCAACACCCTCCTCCAGGGCTACTC
XM_010069017Euc  444 CCACTCGACGACCCGGGCCGCAATGCTCGTGAGGATCAACACCCTCCTCCAGGGCTACTC
XM_010069014Euc  450 CCACTCGGCGACCCGGGCCGCCATGCTCGTGAGGATCAACACCCTCCTCCAGGGCTACTC
XM_010069015Euc  450 CCACTCGGCGACCCGGGCCGCGATGCTCGTGAGGATCAACACCCTCCTCCAAGGCTACTC
XM_010069016Euc  450 CCACTCGGCGACCCGGGCCGCGATGCTCGTGAGGATCAACACCCTCCTCCAAGGCTACTC
Myrtus             1 ------------------------------------------------------------
KU162973Vitis    459 TCACAGTGCGACAAGAGCAGCAATGCTGGTGAGGATCAATACCCTCCTCCAAGGATACTC
XM_008357397Mal  468 ACACCAAGCAACAAGAGCAGCCATGTTGGTCCGAATCAACACACTCCTCCAAGGCTACTC
NM_111869Arabid  432 AAAGCCGACAACAAGAGCGGCAATGCTCGTCCGTGTCAACACTCTCCTCCAAGGCTACTC

XM_010069013Euc  510 GGGCATCCGGTTCGAGATCCTGGAGACCATGGCCAAGCTCCTCAACCGCAACATCACCCC
XM_010069017Euc  504 AGGCATCCGGTTCGAGATCCTGGAGACCATGGCCAAGCTCCTCAACCGCAACATCACCCC
XM_010069014Euc  510 AGGCATCCGGTTTGAGATCCTGGAGACCATGGCCAAGCTCCTCAACCGCAACATCACCCC
XM_010069015Euc  510 AGGCATCCGGTTTGAGATCCTGGAGACCATGGCCAAGCTCCTCAACCGCAACATCACCCC
XM_010069016Euc  510 AGGCATCCGGTTTGAGATCCTGGAGACCATGGCCAAGCTCCTCAACCGCAACATCACCCC
Myrtus             1 ------------------------------------------------------------
KU162973Vitis    519 AGGCATCAGATTTGAGATCTTGGAAGCCACCGTTAAGCTCCTCAACGCCAACATTAGTCC
XM_008357397Mal  528 CGGCATAAGATTCGAAATCTTGGAAGCCATCACCAAGTTCCTGAACAACAACGTTACTCC
NM_111869Arabid  492 CGGTATACGCTTCGAGATTCTCGAAGCAATTACAAAGCTTCTCAACCACGAAATCACTCC

XM_010069013Euc  570 GTGCCTGCCTCTCAGGGGTACGATCACGGCCTCGGGCGACCTGGTCCCCTTGTCCTACAT
XM_010069017Euc  564 ATGCCTGCCTCTCAGGGGTACCGTCACAGCCTCGGGCGACCTGGTCCCCTTGTCCTACAT
XM_010069014Euc  570 GTGCCTGCCTCTCAGGGGTACCGTCACGGCCTCGGGCGACCTGGTCCCCTTGTCCTACAT
XM_010069015Euc  570 GTGCCTGCCTCTCAGGGGTACCGTCACGGCCTCAGGCGACCTGGTCCCCTTGTCCTACAT
XM_010069016Euc  570 GTGCCTGCCTCTCAGGGGTACTGTCACGGCCTCAGGCGACCTGGTCCCCTTGTCCTACAT
Myrtus             1 ------------------------------------------------------------
KU162973Vitis    579 TTGCTTGCCTCTGCGTGGCACCATCAGTGCCTCTGGTGATCTCGTCCCTCTTTCTTACAT
XM_008357397Mal  588 ATGCTTGCCCCTACGCGGTACTATTACCGCCTCCGGAGATCTTGTCCCGCTATCCTACAT
NM_111869Arabid  552 GTGCCTCCCTCTCCGTGGCACCATCACCGCCTCCGGTGACCTTGTTCCTCTCTCTTACAT

XM_010069013Euc  630 CGCCGGGCTTCTGACGGGCAGGCCCAACTCGAAGGCGGTCGGGCCAAACGGCGAGTCCCT
XM_010069017Euc  624 CGCCGGGCTTCTGACGGGCAGGCCCAACTCGAAGGCGGTCGGGCCAAACGGCGAGTCCCT
XM_010069014Euc  630 CGCCGGGCTTCTGACGGGCAGGCCCAACTCGAAGGCGGTCGGGCCAAATGGCGAGTCCCT
XM_010069015Euc  630 CGCCGGGCTTCTGACGGGCAGGCCCAACTCGAAGGCGGTTGGGCCAAATGGCGAGTCCCT
XM_010069016Euc  630 CGCCGGGCTTCTGACGGGCAGGCCCAACTCAAAGGCGGTCGGGCCAAATGGCGAGTCCCT
Myrtus             1 ------------------------------------------------------------
KU162973Vitis    639 TGCCGGTCTCCTCACCGGCAGGCCTAACTCCAGGGCCGTAGGGCCTAACGGAGAGTCCCT
XM_008357397Mal  648 TGCCGGATTACTCACCGGCAGGCCTAATTCCAAAGCTGTCGGACCAAATGGGCAGACCCT
NM_111869Arabid  612 CGCCGGACTTCTCACTGGCCGTCCCAACTCCAAAGCCGTGGGTCCCTCTGGTGAGACTCT
```

```
XM_010069013Euc  690 GAACGCTGTCGAGGCCTTCCGCCTTGCCGGGATCGAGCACGGGTTCTTTGACCTGCAGCC
XM_010069017Euc  684 GAACGCCGTCGAGGCCTTCCGCCTTGCTGGGATCGAGCACGGGTTCTTTGACCTGCAGCC
XM_010069014Euc  690 GAACGCCGTCGAGGCCTTCCGCCTTGCCGGGATCAAGCACGGGTTCTTTGACCTGCAGCC
XM_010069015Euc  690 GAACGCCGTCGAGGCCTTCCACCTTGCCGGGATCAAGCACGGGTTCTTTGACCTGCAGCC
XM_010069016Euc  690 GAACGCCGTCGAGGCCTTCCGCCTTGCCGGGATCAAGCACGGGTTCTTTGACCTGCAGCC
Myrtus             1 ------------------------------------------------------------
KU162973Vitis    699 CAATGCTGAGGAAGCCTTTCGCCTTGCCGGAATTGATGGAGGGTTCTTTGAGTTGCAGCC
XM_008357397Mal  708 CAATGCCTCAGAAGCTTTTGAGCTAGTAGGGATCAATTCCGGGTTTTTTGAGTTGCAGCC
NM_111869Arabid  672 CACTGCCTCTGAGGCCTTTAAGCTCGCCGGAGTATCGTCC---TTTTTCGAGCTGCAGCC

XM_010069013Euc  750 CAAAGAGGGCCTTGCGCTCGTGAATGGCACGGCAGTCGGGTCTGGGCTCGCTTCCATTGT
XM_010069017Euc  744 CAAAGAGGGCCTCGCGATCGTGAATGGCACGGCAGTCGGGTCTGGGCTCGCTTCCATTGT
XM_010069014Euc  750 CAAAGAGGGCCTCGCGATCGTGAATGGCACGGCAGTCGGGTCTGGGCTCGCTTCCATTGT
XM_010069015Euc  750 CAAAGAGGGCCTCGCGATCGTGAATGGCACGGCAGTCGGGTCTGGGCTCGCTTCCATTGT
XM_010069016Euc  750 CAAAGAGGGCCTCGCGATCGTGAATGGCACGGCAGTCGGGTCTGGGCTCGCTTCCATTGT
Myrtus             1 ------------------------------------------------------------
KU162973Vitis    759 TAAGGAGGGTCTTGCACTCGTTAATGGCACGGCAGTAGGGTCTGGTTTGGCTTCAACAGT
XM_008357397Mal  768 TAAAGAAGGGTTAGCTCTTGTGAACGGCACTGCTGTTGGTTCTGGCTTGGCCTCCACGGT
NM_111869Arabid  729 TAAGGAAGGACTAGCACTTGTGAACGGGACAGCGGTTGGATCGGGTTTGGCCTCAACGGT

XM_010069013Euc  810 CCTGTACGAGGCGAACATCCTGGCCGTCCTCTCAGAGGTTCTGTCGGCCATCTTCGCCGA
XM_010069017Euc  804 CCTGTTCGAGGCGAACATCCTGGCCGTCCTCTCAGAGGTTCTGTCGGCGATCTTCGCCGA
XM_010069014Euc  810 CCTGTTCGAGGCGAACATCCTGGCCGTCCTCTCAGAGGTTCTGTCGGCCATCTTCGCCGA
XM_010069015Euc  810 CCTGTTCGAGGTGAACATCCAGGCCGTCCTCTCAGAGGTTCTGTCGGCGATCTTCGCCGA
XM_010069016Euc  810 CCTGTTCGAGGCGAACATCCTGGCCGTCCTCTCAGAGGTTCTGTCGGCGATCTTCGCCGA
Myrtus             1 ------------------------------------------------------------
KU162973Vitis    819 TCTCTTTGAGGCTAACATACTTGCTGTTTTATCAGAAGTTTTGTCGGCTATATTTGCTGA
XM_008357397Mal  828 TCTTTTCGAGACCAACATTTTGGCATTGCTTGCGGAGATTTTATCAGCGATTTTCGCTGA
NM_111869Arabid  789 TTTGTTCGATGCAAATATTTGGCTGTTTTATCGGAAGTTATGTCTGCCATGTTCGCAGA

XM_010069013Euc  870 GGTGATGCAAGGGAAGCCGGAGTTCACCGACCACTTGACCCACAAGCTCAAGCACCATCC
XM_010069017Euc  864 GGTGATGCAAGGGAATCCGGAGTTCACCGACCACTTGACCCACAAGCTCAAGCACCATCC
XM_010069014Euc  870 GGTGATGCAAGGGAAGCCGGAGTTCACCGACCACTTGACCCACAAGCTCAAGCACCATCC
XM_010069015Euc  870 GGTGATGCAAGGGAAGCCGGAGTTCACCGACCACTTGACCCACAAGCTCAAGCACCATCC
XM_010069016Euc  870 GGTGATGCAAGGGAAGCCGGAGTTCACTGACCACTTGACCCACAAGCTCAAGCACCATCC
Myrtus             1 ------------------------------------------------------------
KU162973Vitis    879 GGTTATGCAGGGGAAGCCAGAGTTTACTGATCATTTGACGCACAAATTGAAACACCATCC
XM_008357397Mal  888 AGTGATGCAGGGGAAACCGGAGTTTACTGATCACTTGACGCACAAGTTGAAGCACCACCC
NM_111869Arabid  849 GGTTATGCAAGGGAAACCGGAGTTTACAGATCATCTTACGCATAAGCTCAAGCACCATCC

XM_010069013Euc  930 CGGACAAATTGAAGCTGCAGCTATAATGGAGCACATCTTGGATGGGAGCTCCTACGTGAA
XM_010069017Euc  924 CGGACAAATTGAAGCTGCAGCTATAATGGAGCACATCTTGGATGGGAGCTCCTACGTGAA
XM_010069014Euc  930 CGGACAAATTGAAGCTGCAGCTATAATGGAGCACATCTTGGATGGGAGCTCCTACGTGAA
XM_010069015Euc  930 CGGACAAATTGAAGCTGCAGCTATAATGGAGCACATCTTGGATGGGAGCTCCTACGTGAA
XM_010069016Euc  930 CGGACAAATTGAAGCTGCAGCTATAATGGAGCACATCTTGGATGGGAGCTCCTACGTGAA
Myrtus             1 ------------------------------------------------------------
KU162973Vitis    939 GGGACAAATTGAAGCTGCGGCTATTATGGAACACATTTTGGATGGAAGCGCTTATGTTAA
XM_008357397Mal  948 TGGACAGATTGAGGCAGCTGCAATTATGGAACATATTTTGGATGGCAGCTCTTATGTCAA
NM_111869Arabid  909 CGGTCAGATCGAAGCCGCCGCAATTATGGAACATATATTAGACGGAAGCTCTTACGTTAA

XM_010069013Euc  990 GGACGCTCAAAAGCTCCACGAGATGGACCCTCTCCAGAAGCCGAAGCAGGACCGATACGC
XM_010069017Euc  984 GGACGCTCAAAAGCTCCACGAGATCGACCCTCTCCAGAAGCCGAAGAAGGACCGATACGC
XM_010069014Euc  990 GGACGCTCAAAAGCTCCACGAGATGGACCCTCTCCAGAAGCCGAAGCAGGACCGATACGC
XM_010069015Euc  990 GGACGCTCAAAAGCTCCACGAGATCGACCCTCTCCAGAAGCCGAAGAAGGACCGATACGC
XM_010069016Euc  990 GGACGCTCAAAAGGTCCACGAGATCGACCCTCTCCAGAAGCCGAAGCAGGACCGATACGC
Myrtus             1 ------------------------------------------------------------
KU162973Vitis    999 AGCGGCTCAGATGCTGCACGAGATCGATCCCCTCCAGAAGCCCAAGCAAGATCGGTATGC
XM_008357397Mal 1008 AGCTGCTAAGAAGTTACATGAGCAGGATCCCCTGCAGAAGCCAAAGCAGGATCGCTACGC
NM_111869Arabid  969 AGAAGCTCAACTTCTCCACGAAATGGATCCTCTTCAAAAACCTAAACAAGATCGGTACGC
```

```
XM_010069013Euc 1050 GCTCCGCACGTCCCCGCAGTGGCTCGGCCCCCAGATCGAAGTGATCAGGGCCGCCACCAA
XM_010069017Euc 1044 GCTCCGCACGTCCCCGCAGTGGCTCGGCCCCCAGATCGAAGTGATCAGGGCCGCCACCAA
XM_010069014Euc 1050 GCTCCGCACGTCCCCGCAGTGGCTCGGCCCCCAGATCGAAGTGATCAGGGCCGCCACCAA
XM_010069015Euc 1050 GCTCCGCACGTCCCCGCAGTGGCTCGGCCCCCAGATCGAAGTGATCAGGGCCGCCACCAA
XM_010069016Euc 1050 GCTCCGCACGTCCCCGCAGTGGCTCGGCCCCCAGATCGAAGTGATCAGGGCCGCCACCAA
Myrtus             1 ------------------------------------------------------------
KU162973Vitis   1059 TCTTAGGACTTCACCCCAGTGGTTGGGTCCTCAGATTGAAGTTATTCGAACATCAACGAA
XM_008357397Mal 1068 TCTCCGAACATCACCTCAGTGGCTGGGACCTCAAATTGAAGTGATTCGTTTCTCCACCAA
NM_111869Arabid 1029 TTTACGTACGTCACCACAATGGCTTGGGCCGCAGATTGAAGTGATCAGAGCGGCTACTAA

XM_010069013Euc 1110 AATGATCGAGAGGGAGATCAATTCTGTCAACGACAACCCGCTGATCGACGTCTCCAGGAA
XM_010069017Euc 1104 AATGATCGAGAGGGAGATCAACTCTGTCAATGACAACCCGCTGATCGACGTCTCCAGGAA
XM_010069014Euc 1110 AATGATCGAGAGGGAGATCAACTCTGTCAACGACAACCCGCTGATCGACGTCTCCAGGAA
XM_010069015Euc 1110 AATGATCGAGAGGGAGATCAACTCTGTCAACGACAACCCGCTGATCGACGTCTCCAGGAA
XM_010069016Euc 1110 AATGATCGAGAGGGAGATCAACTCTGTCAACGACAACCCGCTGATCGATGTCTCCAGGAA
Myrtus             1 ------------------------------------------------------------
KU162973Vitis   1119 GTCCATAGAACGAGAGATCAATTCGGTGAATGACAACCCATTGATTGATGTGTCAAGGAA
XM_008357397Mal 1128 ATCCATTGAGAGGGAGATCAACTCGGTTAATGACAACCCTTTGATTGATGTTTCGAGGAA
NM_111869Arabid 1089 AATGATTGAGCGTGAGATCAACTCTGTTAATGATAACCCTTTGATAGATGTGTCGAGGAA

XM_010069013Euc 1170 CAAGGCTCTCCATGGAGGGAACTTCCAGGGGACCCCGATTGGCGTTTCCATGGACAACAC
XM_010069017Euc 1164 CAAGGCTCTCCATGTAGGGAACTTCCAGGGGACCCCGATTGGCGTTTCCATGGACAACAC
XM_010069014Euc 1170 CAAGGCTCTCCATGGAGGGAACTTCCAGGGGACCCCGATTGGCGTTTCCATGGACAACAC
XM_010069015Euc 1170 CAAGGCTCTCCATGGAGGGAACTTCCAGGGGACCCCGATTGGGGTTTCCATGGACAACAC
XM_010069016Euc 1170 CAAGGCTCTCCATGGAGGGAACTTCCAGGGGACCCCGATTGGCGTTTCCATGGACAACAC
Myrtus             1 -------------------------------------------GTTTCCATGGACAACAC
KU162973Vitis   1179 CAAGGCCTTGCATGGTGGGAACTTCCAGGGTACCCCAATTGGTGTTTCCATGGACAACAC
XM_008357397Mal 1188 CAAGGCCTTACATGGAGGCAACTTCCAGGGGACCCCAATTGGGGTTTCCATGGACAACAC
NM_111869Arabid 1149 CAAGGCGTTGCACGGTGGAAATTTCCAAGGGACACCGATCGGTGTTGCCATGGATAATTC

XM_010069013Euc 1230 CCGTCTCGCCATCGCCTCCATCGGCAAGCTCATGTTCGCGCAGTTCTCCGAGCTCGTCAA
XM_010069017Euc 1224 CCGTCTCGCCATCGCCTCCATCGGCAGGCTCATGTTCGCGCAGTTCTCCGAGCTCGTCAA
XM_010069014Euc 1230 CCGTCTCGCCATCGCCTCCATCGGCAAGCTCATGTTTGCGCAGTTCTCCGAGCTCGTTAA
XM_010069015Euc 1230 CCGTCTCGCCATCGCCTCCATCGGCAAGCTCATGTTTGCGCAGTTCTCCGAGCTCGTTAA
XM_010069016Euc 1230 CCGTCTCGCCATCGCCTCCATCGGCAAGCTCATGTTCGCGCAGTTCTCCGAGCTCGTTAA
Myrtus            18 CCGCCTCGCCATCGCCTCAATCGGCAAGCTCATGTTTGCGCAGTTCTCAGAGCTCGTCAA
KU162973Vitis   1239 CAGATTAGCAATAGCCTCCATTGGGAAGCTCATGTTTGCGCAATTCTCTGAGCTGGTTAA
XM_008357397Mal 1248 TCGCTTGGCGATTGCATCCATTGGCAAGCTCATGTTTGCACAATTTTCTGAACTTGTCAA
NM_111869Arabid 1209 CCGTCTAGCCATTGCTTCCATTGGGAAACTCATGTTTGCGCAGTTTTCTGAACTAGTGAA

XM_010069013Euc 1290 CGACTTCTACAACAACGGGCTGCCCTCTAACCTGTCCGGGGGACGGAACCCAAGCTTGGA
XM_010069017Euc 1284 CGACTTCTACAACAACGGGCTGCCCTCTAACCTGTCCGGGGGACGGAACCCAAGCCTGGA
XM_010069014Euc 1290 CGACTTCTACAACAACGGGCTGCCCTCTAACCTGTCCGGGGGACGGAACCCGAGCCTGGA
XM_010069015Euc 1290 CGACTTCTACAACAACGGGCTGCCCTCTAACCTGTCCGGGGGACGGAACCCGAGCCTGGA
XM_010069016Euc 1290 CGACTTCTACAACAACGGGCTGCCCTCTAACCTGTCCGGGGGACGGAACCCGAGCCTGGA
Myrtus            78 TGACTTCTACAATAACGGGCTGCCCTCTAACCTGTCCGGGGGA-----------------
KU162973Vitis   1299 CGATTTTTACAACAATGGGTTGCCCTCAAATCTCTCTGGTGGAAGGAATCCAAGTCTGGA
XM_008357397Mal 1308 TGACTTTTACAACAATGGTTTGCCTTCAAATCTGTCGGGAGGCAGGAATCCTAGCTTGGA
NM_111869Arabid 1269 CGATTTCTACAACAATGGTTTGCCTTCTAATCTATCTGGTGGCAGAAACCCTAGTCTTGA

XM_010069013Euc 1350 TTACGGCTTCAAGGGAGCTGAGATAGCCATGGCCTCATACTGCTCGGAGCTGCAGTTTCT
XM_010069017Euc 1344 TTACGGCTTCAAGGGAGCTGAGATCGCCATGGCCTCATACTGCTCGGAGCTGCAGTTCCT
XM_010069014Euc 1350 TTATGGCTTCAAGGGAGCCGAGATCGCCATGGCCTCATATTGCTCGGAGCTGCAGTTCCT
XM_010069015Euc 1350 TTATGGCTTCAAGGGAGCCGAGATCGCCATGGCCTCATACTGCTCGGAGCTGCAGTTCCT
XM_010069016Euc 1350 TTATGGCTTCAAGGGAGCCGAGATCGCCATGGCCTCATACTGCTCGGAGCTGCAGTTCCT
Myrtus               ------------------------------------------------------------
KU162973Vitis   1359 TTATGGGTTCAAGGGTGCTGAGATAGCAATGGCAGCATATTGTTCTGAGCTCCAATTCCT
XM_008357397Mal 1368 TTACGGTTTCAAGGGGGCTGAAATCGCCATGGCATCTTATTGTTCCGAGCTGCAGTTTCT
NM_111869Arabid 1329 TTACGGGTTTAAAGGCGCGGAAATAGCCATGGCTTCTTATTGCTCCGAGCTTCAGTTCCT
```

```
XM_010069013Euc 1410 TGCCAACCCTGTGACCAACCATGTCCAGAGCGCGGAGCAACACAACCAGGACGTGAACTC
XM_010069017Euc 1404 TGCCAACCCTGTGACCAACCATGTCCAGAGCGCGGAGCAACACAACCAGGACGTGAACTC
XM_010069014Euc 1410 TGCCAACCCTGTGACCAACCATGTCGAGAGCGCGGAGCAACACAACCAGGACGTGAACTC
XM_010069015Euc 1410 TGCCAACCCTGTGACCAACCATGTCGAGAGCGCGGAGCAACACAACCAGGACGTGAACTC
XM_010069016Euc 1410 TGCCAACCCTGTGACCAACCATGTCGAGAGCGCGGAGCAACACAACCAGGACGTGAACTC
Myrtus               ------------------------------------------------------------
KU162973Vitis   1419 AGCCAACCCAGTCACCAATCATGTTCAGAGCGCTGAGCAACACAACCAGGATGTGAACTC
XM_008357397Mal 1428 CGCAAATCCGGTAACTAACCATGTCCAGAGTGCTGAGCAGCACAACCAAGATGTTAACTC
NM_111869Arabid 1389 GGCTAATCCCGTGACCAACCATGTCCAAAGCGCAGAGCAGCATAACCAAGACGTTAATTC

XM_010069013Euc 1470 CTTGGGCCTGATCTCGTCGAGGAAGACCGCCGAGGCCGTCGACGTGCTGAAGCTCATGTC
XM_010069017Euc 1464 CTTGGGCCTGATCTCGTCGAGGAAGACCGCCGAGGCCGTCGATGTGCTGAAGCTCATGTC
XM_010069014Euc 1470 CTTGGGCCTGATCTCGTCGAGGAAGACTGCCGAGGCCGTCGACGTGCTGAAGCTCATGTC
XM_010069015Euc 1470 CTTGGGCCTGATCTCGTCGAGGAAGACTGCCGAGGCCGTCGACGTGCTGAAGCTCATGTC
XM_010069016Euc 1470 CTTGGGCCTGATCTCGTCGAGGAAGACTGCCGAGGCCGTTGACGTGCTGAAGCTCATGTC
Myrtus               ------------------------------------------------------------
KU162973Vitis   1479 CTTAGGATTGATCTCTTCGCGAAAAACGGAAGAAGCAATTGACATCTTAAAGCTCATGTC
XM_008357397Mal 1488 TTTGGGGTTGATCTCTTCAAGGAAAACAGCTGAAGCTGTTGATATCTTGAAGCTCATGTC
NM_111869Arabid 1449 CCTAGGGCTAATCTCTAGCAGGAAAACTGCAGAAGCAGTGGATATCCTCAAGCTAATGTC

XM_010069013Euc 1530 CTCCACCTTCCTGGTCGCCCTGTGCCAGGCCATCGACCTGAGGCACCTGGAAGAGAACCT
XM_010069017Euc 1524 CTCCACCTTCCTGGTCGCCCTATGCCAGGCCATCGACCTGAGGCACCTGGAAGAGAACCT
XM_010069014Euc 1530 CTCCACCTTCCTGGTCGCCCTGTGCCAGGCCATCGACCTGAGGCACCTGGAAGAGAACCT
XM_010069015Euc 1530 CTCCACCTTCCTGGTCGCCCTGTGCCAGGCCATCGACCTGAGGCACCTGGAAGAGAACCT
XM_010069016Euc 1530 CTCCACCTTCCTGGTCGCCCTGTGCCAGGCCATCGACCTGAGGCACCTGGAAGAGAACCT
Myrtus               ------------------------------------------------------------
KU162973Vitis   1539 CTCAACCTACCTGGTGGCACTTTGCCAGGCTATTGATTTGAGGCATTTGGAGGAGAACTT
XM_008357397Mal 1548 TTCCACATTTTTGGTTGCGCTTTGTCAGTCCATAGATTTGAGGCATTTGGAGGAGAACTT
NM_111869Arabid 1509 CACAACCTACTTAGTCGCGCTTTGCCAAGCCGTTGATCTAAGACATCTTGAAGAGAATCT

XM_010069013Euc 1590 CAAGAGCGTGGTCAAGAACACGGTGAACCAAGTGGCCAAGAAGGTCCTCTACGTCGGGTC
XM_010069017Euc 1584 CAAGAGCGTAGTCAAGAACACGGTGAACCAAGTGGCCAAGAAGGTCCTCTACGTCGGGTC
XM_010069014Euc 1590 CAAGAGCGTGGTCAAGAGCACGGTGAACCAAGTGGCCAAGAAGGTCCTCTACGTCGGGTC
XM_010069015Euc 1590 CAAGAGCGTGGTCAAGAACACGGTGAACCAAGTGGCCAAGAAGGTCCTCTACGTCGGGTC
XM_010069016Euc 1590 CAAGAGCGTGGTCAAGAACACGGTGAACCAAGTGGCCAAGAAGGTCCTCTACGTTGGGTC
Myrtus               ------------------------------------------------------------
KU162973Vitis   1599 GAAGAACACAGTCAAGAACACAGTGAGCCAAGTGGCCAAAAAGGTGCTAGCCATTGGGAT
XM_008357397Mal 1608 GAGGAACACTGTGAAGAACACGGTGAGCCAAGTAGCAAAGAGGACTTTAACAACTGGGGT
NM_111869Arabid 1569 GAAGAAGGCGGTTAAATCAGCAGTGAGTCAGGTGGCGAAACGGGTCTTAACCGTTGGTGC

XM_010069013Euc 1650 CAACGGCGAGCTCCACCCGTCGCGGTTCAGCGAGAAAGACCTGATCAAGGTGGTCGACCG
XM_010069017Euc 1644 CAACGACGAGCTCCACCCGTCGCGGTTCAGCGAGAAAGACCTGATCAAGGTGGTCGACCG
XM_010069014Euc 1650 CAACGGCGAGCTCCACCCGTCGCGGTTCAGCGAGAAAGACCTGATCAAGGTGGTCGACCG
XM_010069015Euc 1650 CAACGGCGAGCTCCACCCGTCGCGGTTCAGCGAGAAAGACCTGATCAAGGTGGTCGACCG
XM_010069016Euc 1650 CAACGGCGAGCTCCACCCGTCGCGGTTCAGCGAGAAAGACCTGATCAAGGTGGTCGACCG
Myrtus               ------------------------------------------------------------
KU162973Vitis   1659 CAATGGGGAGCTTCACCCGTCTAGATTCTGCGAGAAGGACTTGCTTAAAGTAGTAGACCG
XM_008357397Mal 1668 TAATGGAGAGCTTCACCCCTCAAGGTTCTGTGAGAAGGATCTGCTTAAAGTTGTCGATAG
NM_111869Arabid 1629 CAACGGGGAGCTACATCCGTCAAGGTTCACAGAACGTGATGTCCTCCAAGTGGTTGACCG

XM_010069013Euc 1710 GGAGTACGTCTTCGCCTACATCGATGACCCCTGCAGCGCCACGTACCCCCTGATGCAGAA
XM_010069017Euc 1704 GGAGTACGTCTTCGCCTACATCGATGACCCCTGCAGCGCCACGTACCCCCTGATGCAGAA
XM_010069014Euc 1710 GGAGCACGTCTTCGCCTACATTGATGACCCCTGCAGCGCCACGTACCCCCTGATGCAGAC
XM_010069015Euc 1710 GGAGCACGTCTTCGCCTACATTGATGACCCCTGTAGCGCCACGTACCCCCTGATGCAGAC
XM_010069016Euc 1710 GGAGCACGTCTTCGCCTACATTGATGACCCCTGCAGCGCCACGTACCCCCTGATGCAGAA
Myrtus               ------------------------------------------------------------
KU162973Vitis   1719 CGAGCACGTTTTTGCCTACATCGATGACCCATGCAGTGCAACATACCCCTTGATGCAAAA
XM_008357397Mal 1728 GGAGTATGTTTTTGCCTACATTGACGATCCCTGCAGTGCCACTTACCCATTGATGCAAAA
NM_111869Arabid 1689 AGAGTACGTGTTCTCATACGCAGACGATCCCTGCAGCCTCACTTACCCGCTAATGCAGAA
```

```
XM_010069013Euc 1770 ACTGAGGCAGGTCCTCGTGGACGATGCGCTGGACGACGTCGACCGGGAGAAGAACCCCAG
XM_010069017Euc 1764 GCTGAGGCAGGTCCTCGTGGACGATGCGCTGGACGACGTCGACCGGGAGATGAACCCCAG
XM_010069014Euc 1770 GCTGAGGCAGGTCCTCGTGGACCATGCGCTGGACGATGTCGACCGGGAGAAGAACCCCAG
XM_010069015Euc 1770 GCTGAGGCAGGTCCTCGTGGACCATGCGCTGGACGACGTCGACCGAGAGAAGAACCCCAG
XM_010069016Euc 1770 GCTGAGGCAGGTCCTCGTGGACCATGCACTGGACGACGTCGACCGGGAGAAGAACCCCAG
Myrtus               ------------------------------------------------------------
KU162973Vitis   1779 CCTAAGGCAAGTCCTGGTTGATCATGCGCTGATGAACAACGAGGATTTGAGGAATTCAAG
XM_008357397Mal 1788 ACTAAGGGAAGTGCTGGTTGAACATGCTTTGACAAATGGCGAGAGTGAGAAGAATGCAAG
NM_111869Arabid 1749 ACTTAGACACATTCTTGTAGACCACGCTTTAGCGGATCCAGAACGCGAGGCCAATTCCGC

XM_010069013Euc 1830 CACCTCCATCTTCCAGAAGATTGGGGCTTTCGAGGAGGAGCTCAAGGCACTCCTCCCGAA
XM_010069017Euc 1824 CACCTCCATCTTCCAGAAGATTGGGACTTTCGAGGAGGAGCTCAAGGCACTCCTCCCGAA
XM_010069014Euc 1830 CACCTCCATCTTCCAGAAGATTGGGGCTTTCGAGGAGCAGCTCAAGGCACTCCTCCCGAA
XM_010069015Euc 1830 CACCTCCATCTTCCAGAAGATTGGGGCTTTCGAGGAGCAGCTCAAGGTACTCCTCCCGCA
XM_010069016Euc 1830 CACCTCCATCTTCCAGAAGATTGGGGCTTTCGAGGAGCAGCTCAAGGCACTCCTCCCGAA
Myrtus               ------------------------------------------------------------
KU162973Vitis   1839 TACTTCAATCTTTCTCAAGATTGGGGCATTTGAGGAAGAACTGAAGACCCTTTTGCCTAA
XM_008357397Mal 1848 CACTTCGATCTTCCAAAAGATTGGAGCTTTCGAGGAAGAGCTGAAAGCGCTTTTGCCTAA
NM_111869Arabid 1809 GACATCGGTTTTCCACAAAATCGGAGCTTTTGAAGCCGAGCTGAAACTGCTTCTCCCTAA

XM_010069013Euc 1890 GGAGGTCGAGAACGCGAGAGCTCAGTTCGAGAGCGGGAACTCGGCGATCGCTAACAAGAT
XM_010069017Euc 1884 GGAGGTCGAGAACGCGAGAGCTCAGTTCGAGAGCGGGAACTCGGCGATCGCTAACAAGAT
XM_010069014Euc 1890 GGAGGTCGAGAACGCGAGAGCTCAGTTCGAGAGCGGGAACTCGGCGATCGCTAACAAGAT
XM_010069015Euc 1890 GGAGGTCGAGAACGCGAGAGCTCAGTTCGAGAGCGGGAGCTCGGCGATCGCTAACAAGAT
XM_010069016Euc 1890 GGAGGTTGAGAACGCGAGAGCTCAGTTCGAGAGCGGGAACTCGGCGATCGCTAACAAGAT
Myrtus               ------------------------------------------------------------
KU162973Vitis   1899 AGACGTGGAGAGCACGAGAATTGCCTTTGATAATGGGAATCTGGCCATTCCAAACCGCAT
XM_008357397Mal 1908 AGAGGTTGAGAGTGCAAGGAGTGCAATTGAGAGCGGAAATGCTGCAGTTCCAAACAGGAT
NM_111869Arabid 1869 AGAAGTAGAACGCGTCCGGGTTGAATACGAGGAAGGAACATCGGCTATAGCTAACCGGAT

XM_010069013Euc 1950 CAGGGGGTGCAGGTCGTACCCATTGTACAGGTTCGTGAGGGAAGAGCTCGGGACCGGTTT
XM_010069017Euc 1944 CAGGGGGTGCAGGTCGTACCCATTGTACAGGTTCGTGAGGGAAGAGCTCGGGACCGGTTT
XM_010069014Euc 1950 CAGGGGGTGCAGGTCGTACCCATTGTACAGGTTCGTGAGGGAAGAGCTCGGGACCGGTTT
XM_010069015Euc 1950 CAGGGGGTGCAGGTCGTACCCATTGTACAGGTTCGTGAGGGAAGAGCTCGGGACCGGTTT
XM_010069016Euc 1950 CAGGGGGTGCAGGTCGTACCCATTGTACAGGTTCGTGAGGGAAGAGCTCGGGACCGGTTT
Myrtus               ------------------------------------------------------------
KU162973Vitis   1959 CAAGGAGTGCAGGTCCTACCCTCTGTACAAGTTTGTGAGGGAAGAGTCAGGGACTGAGTT
XM_008357397Mal 1968 TGCGGAATGCAGATCTTATCCCTTGTACAAGTTTGTGAGGGAGGAGTTGGGGGGAGAGTA
NM_111869Arabid 1929 TAAGGAATGTCGGTCTTATCCATTGTATCGGTTTGTCCGCGATGAGCTAAATACTGAACT

XM_010069013Euc 2010 GCTCACGGGGGAGAAGGTCGGCTCGCCGGGCGAGGACTTTGATCTGGTGTTCTCCGCCAT
XM_010069017Euc 2004 GCTCACGGGGGAGAAGGTCGGCTCGCCGGGCGAGGACTTTGATCTGGTGTTCTCCGCCAT
XM_010069014Euc 2010 GCTCACGGGGGAGAAGGTCGGCTCGCCGGGCGAGGACTTTGATCTGGTGTTCTCCGCCAT
XM_010069015Euc 2010 GCTCACGGGGGAGAAGGTCGGCTCGCCGGGCGAGGACTTTGATCTGGTGTTCTCCGCCAT
XM_010069016Euc 2010 GCTCACGGGGGAGAAGGTCGGCTCACCGGGCGAGGACTTTGATCTGGTGTTCTCCGCCAT
Myrtus               ------------------------------------------------------------
KU162973Vitis   2019 TCTGACAGGAGAAAAGGTTACCTCGCCAGGGGAGGAGTTTGATAAGGTGTTCTCTGCAAT
XM_008357397Mal 2028 CCTAACCGGGGAGAAGGTCAGGTCACCGGGCGAGGAGTGTGACAAGGTGTTCCAAGCCAT
NM_111869Arabid 1989 GCTTACTGGAGAGAATGTTCGGTCGCCAGGAGAGGAGTTTGATAAAGTGTTCTTAGCGAT

XM_010069013Euc 2070 GTGCGAGGGCAAGCTGATCGATCCTTTGCTGGAGTGCTTGAGGGACTGGGATGGTGCTCC
XM_010069017Euc 2064 GTGCGAGGGCAAGCTGATCGATCCTTTGCTGGAGTGCTTGAGGGACTGGGATGGTGCTCC
XM_010069014Euc 2070 GTGCGAGGGCAAGCTGATCGATCCTTTGCTGGAGTGCTTGAGGGACTGGGATGGTGCTCC
XM_010069015Euc 2070 GTGCGAGGGCAAGCTGATCGATCCTTTGCTGGAGTGCTTGAGGGACTGGGATGGTGCTCC
XM_010069016Euc 2070 GTGCGAGGGCAAGCTGATCGATCCTTTGCTGGAGTGCTTGAAGGACTGGGATGGTGCTCC
Myrtus               ------------------------------------------------------------
KU162973Vitis   2079 ATGTGAGGGGAAGATCATCGATCCGCTGCTCAAATGTCTCAATGATTGGGATGGGACTCC
XM_008357397Mal 2088 CTGCCAGGGTAAGATTATCGACCCAATTCTGGGTTGCCTCGAGGGTTGGAACGGCGCACC
NM_111869Arabid 2049 TTCTGATGGAAAACTTATTGATCCGTTGTTGGAATGTCTCAAGGAGTGGAACGGAGCTCC
```

```
XM_010069013Euc 2130 GCTTCCCATCTGTTAG--------------------------------------------
XM_010069017Euc 2124 GCTTCCCATCTGTTAGAAACTTTTCCAAATCCTGAAGACTGCCTCATAATTTAACTTGCC
XM_010069014Euc 2130 GCTTCCCATCTGTTAG--------------------------------------------
XM_010069015Euc 2130 GCTTCCCATCTGTTAG--------------------------------------------
XM_010069016Euc 2130 GCTTCCCATCTGTTAG--------------------------------------------
Myrtus               ------------------------------------------------------------
KU162973Vitis   2139 TCTTCCAATTTGCTGA--------------------------------------------
XM_008357397Mal 2148 TCTGCCTATCTGTTAG--------------------------------------------
NM_111869Arabid 2109 GGTTTCAATCTGTTGA--------------------------------------------

XM_010069013Euc      ------------------------------------------------------------
XM_010069017Euc 2184 CACTTAGAGAGTTTTTCATGGTCTAGAAACTGTGGTTAAAATTGTAATGTATCTTATGTC
XM_010069014Euc      ------------------------------------------------------------
XM_010069015Euc      ------------------------------------------------------------
XM_010069016Euc      ------------------------------------------------------------
Myrtus               ------------------------------------------------------------
KU162973Vitis        ------------------------------------------------------------
XM_008357397Mal      ------------------------------------------------------------
NM_111869Arabid      ------------------------------------------------------------

XM_010069013Euc      ------------------------------------------------------------
XM_010069017Euc 2244 TTTTGTTCCCCCGCCAACCACACCCCCACGCCCGCCACCCCCCCCAACGATGTATATTTG
XM_010069014Euc      ------------------------------------------------------------
XM_010069015Euc      ------------------------------------------------------------
XM_010069016Euc      ------------------------------------------------------------
Myrtus               ------------------------------------------------------------
KU162973Vitis        ------------------------------------------------------------
XM_008357397Mal      ------------------------------------------------------------
NM_111869Arabid      ------------------------------------------------------------

XM_010069013Euc      ------------------------------------------------------------
XM_010069017Euc 2304 TACTTGGAACTTCATCGGTGTAACAACAATGTTAAAACGCGTGAAGGAAAAGATCATACA
XM_010069014Euc      ------------------------------------------------------------
XM_010069015Euc      ------------------------------------------------------------
XM_010069016Euc      ------------------------------------------------------------
Myrtus               ------------------------------------------------------------
KU162973Vitis        ------------------------------------------------------------
XM_008357397Mal      ------------------------------------------------------------
NM_111869Arabid      ------------------------------------------------------------

XM_010069013Euc      ---------------------------------------------------
XM_010069017Euc 2364 ACATACATTTCGTTACATTCTTCGTTCTTATAAAGGATCGTGACATTTACT
XM_010069014Euc      ---------------------------------------------------
XM_010069015Euc      ---------------------------------------------------
XM_010069016Euc      ---------------------------------------------------
Myrtus               ---------------------------------------------------
KU162973Vitis        ---------------------------------------------------
XM_008357397Mal      ---------------------------------------------------
NM_111869Arabid      ---------------------------------------------------
```

Figure S2. Alignment of the translated amplicon-amino acid sequence from *M. communis* and the full-length PAL sequences from *E. grandis, V. vinifera, M. domestica* and *A. thaliana*. Identical amino acids are shaded in black, and similar amino acids are shaded in grey. The remaining residues are in a white background.

```
XP_010067316Euc     1 ------MDMDTAACNGEGSLGLCAGAGGDPLNWGAAAAALTGSHLDEVKRMVEESRRPVV
XP_010067317Euc     1 ------MDMDTAACNGKGSLGLCAGAGGDPLNWGAAAAALTGSHLDEVKRMVEESRRPVV
XP_010067315Euc     1 ------MDMDTAACNGKGSLGLCVGAGGDPLNWGAAAAALTGSHLDEVKRMVEESRRPVV
XP_010067318Euc     1 ------MDMDTAACNGKGSLGLCAGAGGDPLNWGVAAAALTGSHLDEVKRMVEESRRPVV
XP_010067319Euc     1 ------MDMDTAACNGKGWLGLCSGAGGDPLNWGAAAAALTGSHLDEVKRMVEESRRPVV
XP_008355619Mal     1 MEAKTITQNGQNGHHQNGVSESQLCIKKDPLNWGLVADSLKGSHLDEVKRMVAEYRKPVV
NP_187645Arabid     1 -------------MELCNQNNHITAVSGDPLNWNATAEALKGSHLDEVKRMVKEYRKEAV
ANB59162Vitis       1 ---MEFSHHNGNGNGKVDANGFCMKA-ADPLYWAAAAETLKGSHLEEVKRMVADFRKPVV
Myrtus              1 ------------------------------------------------------------

XP_010067316Euc    55 RLGGETLTIAQVAAIAG-REAGAAVELSEAARAAVKASSDWVMESMRNGTDSYGVTTGFG
XP_010067317Euc    55 RLGGETLTIAQVAAIAG-REAGAAVELSEAARAAVKASSDWVMESMRNGTDSYGVTTGFG
XP_010067315Euc    55 RLGGETLTIAQVAAIAG-REAGMAVELSEAARAGVKASSDWVMESMSKGTDSYGVTTGFG
XP_010067318Euc    55 RLGGETLTIAQVAAIAG-REAGMAVELSEAARAAVKASSDWVMESMSNGTDSYGVTTGFG
XP_010067319Euc    55 RLGGETLTIAQVAAIAG-QEAGAAVELLEGARAAVKASSDWVMESMSNGTDSYGITTGFG
XP_008355619Mal    61 KLGGESLTISQVAAIAT-HDTGVKVELSESARAGVKASSDWVMDSMGKGTDSYGVTTGFG
NP_187645Arabid    48 KLGGETLTIGQVAAVAR-GGGGSTVELAEEARAGVKASSEWVMESMNRGTDSYGVTTGFG
ANB59162Vitis      57 RLGGESLNIAQVAAVAATGSDGVTVELSESARAGVQASSNWVMESMNQGTDSYGVTTGFG
Myrtus              1 ------------------------------------------------------------

XP_010067316Euc   114 ATSHRRTKQGGALQTELIRFLNAGIFGNGTES-CHMLPHSATRAAMLVRINTLLQGYSGI
XP_010067317Euc   114 ATSHRRTKQGGALQTELIRFLNAGIFGNGTES-CHVLPHSATRAAMLVRINTLLQGYSGI
XP_010067315Euc   114 ATSHRRTKQGGALQRELIRFLNAGIFGNGTES-CHTLPHSATRAAMLVRINTLLQGYSGI
XP_010067318Euc   114 ATSHRRTKQGGALQTELIRFLNAGIFGNGTES-CHVLPHSATRAAMLVRINTLLQGYSGI
XP_010067319Euc   114 ATSHRRTKQGGALQMELIRFLNAGIFGNGTES-CHTLPHSTTRAAMLVRINTLLQGYSGI
XP_008355619Mal   120 ATSHRRTNKGAALQKELIRFLNAGVFGSATES-GHTLPHQATRAAMLVRINTLLQGYSGI
NP_187645Arabid   107 ATSHRRTKQGGALQNELIRFLNAGIFGPGAGDTSHTLPKPTTRAAMLVRVNTLLQGYSGI
ANB59162Vitis     117 ATSHRRTKQGNALQKELIRFLNAGIFGKGTES-CHTLPHSATRAAMLVRINTLLQGYSGI
Myrtus              1 ------------------------------------------------------------

XP_010067316Euc   173 RFEILETMAKLLNRNITPCLPLRGTVTASGDLVPLSYIAGLLTGRPNSKAVGPNGESLNA
XP_010067317Euc   173 RFEILETMAKLLNRNITPCLPLRGTVTASGDLVPLSYIAGLLTGRPNSKAVGPNGESLNA
XP_010067315Euc   173 RFEILETMAKLLNRNITPCLPLRGTITASGDLVPLSYIAGLLTGRPNSKAVGPNGESLNA
XP_010067318Euc   173 RFEILETMAKLLNRNITPCLPLRGTVTASGDLVPLSYIAGLLTGRPNSKAVGPNGESLNA
XP_010067319Euc   173 RFEILETMAKLLNRNITPCLPLRGTVTASGDLVPLSYIAGLLTGRPNSKAVGPNGESLNA
XP_008355619Mal   179 RFEILEAITKFLNNNVTPCLPLRGTITASGDLVPLSYIAGLLTGRPNSKAVGPNGQTLNA
NP_187645Arabid   167 RFEILEAITKLLNHEITPCLPLRGTITASGDLVPLSYIAGLLTGRPNSKAVGPSGETLTA
ANB59162Vitis     176 RFEILEATVKLLNANISPCLPLRGTISASGDLVPLSYIAGLLTGRPNSRAVGPNGESLNA
Myrtus              1 ------------------------------------------------------------

XP_010067316Euc   233 VEAFRLAGIKHGFFDLQPKEGLAIVNGTAVGSGLASIVLFEANILAVLSEVLSAIFAEVM
XP_010067317Euc   233 VEAFHLAGIKHGFFDLQPKEGLAIVNGTAVGSGLASIVLFEVNIQAVLSEVLSAIFAEVM
XP_010067315Euc   233 VEAFRLAGIEHGFFDLQPKEGLALVNGTAVGSGLASIVLYEANILAVLSEVLSAIFAEVM
XP_010067318Euc   233 VEAFRLAGIKHGFFDLQPKEGLAIVNGTAVGSGLASIVLFEANILAVLSEVLSAIFAEVM
XP_010067319Euc   233 VEAFRLAGIEHGFFDLQPKEGLAIVNGTAVGSGLASIVLFEANILAVLSEVLSAIFAEVM
XP_008355619Mal   239 SEAFELVGINSGFFELQPKEGLALVNGTAVGSGLASTVLFETNILALLAEILSAIFAEVM
NP_187645Arabid   227 SEAFKLAG-VSSFFELQPKEGLALVNGTAVGSGLASTVLFDANILAVLSEVMSAMFAEVM
ANB59162Vitis     236 EEAFRLAGIDGGFFELQPKEGLALVNGTAVGSGLASTVLFEANILAVLSEVLSAIFAEVM
Myrtus              1 ------------------------------------------------------------

XP_010067316Euc   293 QGKPEFTDHLTHKLKHHPGQIEAAAIMEHILDGSSYVKDAQKLHEMDPLQKPKQDRYALR
XP_010067317Euc   293 QGKPEFTDHLTHKLKHHPGQIEAAAIMEHILDGSSYVKDAQKLHEIDPLQKPKKDRYALR
XP_010067315Euc   293 QGKPEFTDHLTHKLKHHPGQIEAAAIMEHILDGSSYVKDAQKLHEMDPLQKPKQDRYALR
XP_010067318Euc   293 QGKPEFTDHLTHKLKHHPGQIEAAAIMEHILDGSSYVKDAQKVHEIDPLQKPKQDRYALR
XP_010067319Euc   293 QGNPEFTDHLTHKLKHHPGQIEAAAIMEHILDGSSYVKDAQKLHEIDPLQKPKKDRYALR
XP_008355619Mal   299 QGKPEFTDHLTHKLKHHPGQIEAAAIMEHILDGSSYVKAAKKLHEQDPLQKPKQDRYALR
NP_187645Arabid   286 QGKPEFTDHLTHKLKHHPGQIEAAAIMEHILDGSSYVKEAQLLHEMDPLQKPKQDRYALR
ANB59162Vitis     296 QGKPEFTDHLTHKLKHHPGQIEAAAIMEHILDGSAYVKAAQMLHEIDPLQKPKQDRYALR
Myrtus              1 ------------------------------------------------------------
```

```
XP_010067316Euc  353 TSPQWLGPQIEVIRAATKMIEREINSVNDNPLIDVSRNKALHGGNFQGTPIGVSMDNTRL
XP_010067317Euc  353 TSPQWLGPQIEVIRAATKMIEREINSVNDNPLIDVSRNKALHGGNFQGTPIGVSMDNTRL
XP_010067315Euc  353 TSPQWLGPQIEVIRAATKMIEREINSVNDNPLIDVSRNKALHGGNFQGTPIGVSMDNTRL
XP_010067318Euc  353 TSPQWLGPQIEVIRAATKMIEREINSVNDNPLIDVSRNKALHGGNFQGTPIGVSMDNTRL
XP_010067319Euc  353 TSPQWLGPQIEVIRAATKMIEREINSVNDNPLIDVSRNKALHVGNFQGTPIGVSMDNTRL
XP_008355619Mal  359 TSPQWLGPQIEVIRFSTKSIEREINSVNDNPLIDVSRNKALHGGNFQGTPIGVSMDNTRL
NP_187645Arabid  346 TSPQWLGPQIEVIRAATKMIEREINSVNDNPLIDVSRNKALHGGNFQGTPIGVAMDNSRL
ANB59162Vitis    356 TSPQWLGPQIEVIRTSTKSIEREINSVNDNPLIDVSRNKALHGGNFQGTPIGVSMDNTRL
Myrtus             1 ----------------------------------------------------VSMDNTRL

XP_010067316Euc  413 AIASIGKLMFAQFSELVNDFYNNGLPSNLSGGRNPSLDYGFKGAEIAMASYCSELQFLAN
XP_010067317Euc  413 AIASIGKLMFAQFSELVNDFYNNGLPSNLSGGRNPSLDYGFKGAEIAMASYCSELQFLAN
XP_010067315Euc  413 AIASIGKLMFAQFSELVNDFYNNGLPSNLSGGRNPSLDYGFKGAEIAMASYCSELQFLAN
XP_010067318Euc  413 AIASIGKLMFAQFSELVNDFYNNGLPSNLSGGRNPSLDYGFKGAEIAMASYCSELQFLAN
XP_010067319Euc  413 AIASIGRLMFAQFSELVNDFYNNGLPSNLSGGRNPSLDYGFKGAEIAMASYCSELQFLAN
XP_008355619Mal  419 AIASIGKLMFAQFSELVNDFYNNGLPSNLSGGRNPSLDYGFKGAEIAMASYCSELQFLAN
NP_187645Arabid  406 AIASIGKLMFAQFSELVNDFYNNGLPSNLSGGRNPSLDYGFKGAEIAMASYCSELQFLAN
ANB59162Vitis    416 AIASIGKLMFAQFSELVNDFYNNGLPSNLSGGRNPSLDYGFKGAEIAMAAYCSELQFLAN
Myrtus             9 AIASIGKLMFAQFSELVNDFYNNGLPSNLSGG----------------------------

XP_010067316Euc  473 PVTNHVESAEQHNQDVNSLGLISSRKTAEAVDVLKLMSSTFLVALCQAIDLRHLEENLKS
XP_010067317Euc  473 PVTNHVESAEQHNQDVNSLGLISSRKTAEAVDVLKLMSSTFLVALCQAIDLRHLEENLKS
XP_010067315Euc  473 PVTNHVQSAEQHNQDVNSLGLISSRKTAEAVDVLKLMSSTFLVALCQAIDLRHLEENLKS
XP_010067318Euc  473 PVTNHVESAEQHNQDVNSLGLISSRKTAEAVDVLKLMSSTFLVALCQAIDLRHLEENLKS
XP_010067319Euc  473 PVTNHVQSAEQHNQDVNSLGLISSRKTAEAVDVLKLMSSTFLVALCQAIDLRHLEENLKS
XP_008355619Mal  479 PVTNHVQSAEQHNQDVNSLGLISSRKTAEAVDILKLMSSTFLVALCQSIDLRHLEENLRN
NP_187645Arabid  466 PVTNHVQSAEQHNQDVNSLGLISSRKTAEAVDILKLMSTTYLVALCQAVDLRHLEENLKK
ANB59162Vitis    476 PVTNHVQSAEQHNQDVNSLGLISSRKTEEAIDILKLMSSTYLVALCQAIDLRHLEENLKN
Myrtus               ------------------------------------------------------------

XP_010067316Euc  533 VVKSTVNQVAKKVLYVGSNGELHPSRFSEKDLIKVVDREHVFAYIDDPCSATYPLMQTLR
XP_010067317Euc  533 VVKNTVNQVAKKVLYVGSNGELHPSRFSEKDLIKVVDREHVFAYIDDPCSATYPLMQTLR
XP_010067315Euc  533 VVKNTVNQVAKKVLYVGSNGELHPSRFSEKDLIKVVDREYVFAYIDDPCSATYPLMQKLR
XP_010067318Euc  533 VVKNTVNQVAKKVLYVGSNGELHPSRFSEKDLIKVVDREHVFAYIDDPCSATYPLMQKLR
XP_010067319Euc  533 VVKNTVNQVAKKVLYVGSNDELHPSRFSEKDLIKVVDREYVFAYIDDPCSATYPLMQKLR
XP_008355619Mal  539 TVKNTVSQVAKRTLTTGVNGELHPSRFCEKDLLKVVDREYVFAYIDDPCSATYPLMQKLR
NP_187645Arabid  526 AVKSAVSQVAKRVLTVGANGELHPSRFTERDVLQVVDREYVFSYADDPCSLTYPLMQKLR
ANB59162Vitis    536 TVKNTVSQVAKKVLAIGINGELHPSRFCEKDLLKVVDREHVFAYIDDPCSATYPLMQNLR
Myrtus               ------------------------------------------------------------

XP_010067316Euc  593 QVLVDHALDDVDREKNPSTSIFQKIGAFEEQLKALLPKEVENARAQFESGNSAIANKIRG
XP_010067317Euc  593 QVLVDHALDDVDREKNPSTSIFQKIGAFEEQLKVLLPQEVENARAQFESGSSAIANKIRG
XP_010067315Euc  593 QVLVDDALDDVDREKNPSTSIFQKIGAFEEELKALLPKEVENARAQFESGNSAIANKIRG
XP_010067318Euc  593 QVLVDHALDDVDREKNPSTSIFQKIGAFEEQLKALLPKEVENARAQFESGNSAIANKIRG
XP_010067319Euc  593 QVLVDDALDDVDREMNPSTSIFQKIGTFEEELKALLPKEVENARAQFESGNSAIANKIRG
XP_008355619Mal  599 EVLVEHALTNGESEKNASTSIFQKIGAFEEELKALLPKEVESARSAIESGNAAVPNRIAE
NP_187645Arabid  586 HILVDHALADPEREANSATSVFHKIGAFEAELKLLLPKEVERVRVEYEEGTSAIANRIKE
ANB59162Vitis    596 QVLVDHALMNNEDLRNSSTSIFLKIGAFEEELKTLLPKDVESTRIAFDNGNLAIPNRIKE
Myrtus               ------------------------------------------------------------

XP_010067316Euc  653 CRSYPLYRFVREELGTGLLTGEKVGSPGEDFDLVFSAMCEGKLIDPLLECLRDWDGAPLP
XP_010067317Euc  653 CRSYPLYRFVREELGTGLLTGEKVGSPGEDFDLVFSAMCEGKLIDPLLECLRDWDGAPLP
XP_010067315Euc  653 CRSYPLYRFVREELGTGLLTGEKVGSPGEDFDLVFSAMCEGKLIDPLLECLRDWDGAPLP
XP_010067318Euc  653 CRSYPLYRFVREELGTGLLTGEKVGSPGEDFDLVFSAMCEGKLIDPLLECLKDWDGAPLP
XP_010067319Euc  653 CRSYPLYRFVREELGTGLLTGEKVGSPGEDFDLVFSAMCEGKLIDPLLECLRDWDGAPLP
XP_008355619Mal  659 CRSYPLYKFVREELGGEYLTGEKVRSPGEECDKVFQAICQGKIIDPILGCLEGWNGAPLP
NP_187645Arabid  646 CRSYPLYRFVRDELNTELLTGENVRSPGEEFDKVFLAISDGKLIDPLLECLKEWNGAPVS
ANB59162Vitis    656 CRSYPLYKFVREESGTEFLTGEKVTSPGEEFDKVFSAICEGKIIDPLLKCLNDWDGTPLP
Myrtus               ------------------------------------------------------------
```

```
XP_010067316Euc  713 IC
XP_010067317Euc  713 IC
XP_010067315Euc  713 IC
XP_010067318Euc  713 IC
XP_010067319Euc  713 IC
XP_008355619Mal  719 IC
NP_187645Arabid  706 IC
ANB59162Vitis    716 IC
Myrtus               --
```

Figure S3. Alignment of the partial nucleotide sequence of *CHS* from *M. communis* and the full-length sequences from *E. grandis, V. vinifera, M. domestica* and *A. thaliana*. Identical nucleotides among the sequences are in a black background, whereas those that are similar among the nucleotide sequences are in grey background. The dashes indicate gaps introduced to improve alignment.

```
Myrtus             1 ------------------------------------------------------------
XM_010030619Euc    1 ------ATGGCGGCTACCAAGTCAGTGGAGGAGATCAGGAAGGCGCAGCGGGCGGAGGGC
JF808008Vitis      1 ---------------ATGGTGACCGTCAACGAAGTTCGCAATGCCCAGCGCGCCGAAGGC
AB074485Malus      1 ---------------ATGGTTACAGTCGAGGAAGTTCGCAAGGCTCAACGGGCGGAGGGT
DQ062406Arabido    1 ATGGTGATGGCTGGTGCTTCTTCTTTGGATGAGATCAGACAGGCTCAGAGAGCTGATGGA

Myrtus             1 ------------------------------------------------------------
XM_010030619Euc   55 CCTGCCACCGTGCTCGCCATCGGGACGGCCACCCCAAACAACTGCATCTCCCAGGCGGAC
JF808008Vitis     46 CCAGCCACCGTGATGGCCATCGGCACTGCCACTCCCCCAAATTGTGTGGATCAAAGCACC
AB074485Malus     46 CCAGCCACAGTCATGGCCATCGGGACAGCAACTCCTTCCAACTGTGTGGATCAGGCTACC
DQ062406Arabido   61 CCTGCAGGCATCTTGGCTATTGGCACTGCTAACCCTGAGAACCATGTGCTTCAGGCGGAG

Myrtus             1 ------------------------------------------------------------
XM_010030619Euc  115 TACCCGGACTACTACTTCCGGATCACCAACAGCGAGCACATGACCGACCTCAAGGAGAAG
JF808008Vitis    106 TATCCTGACTACTACTTTCGCATCACAAATAGCGAACACAAGACTGAGCTCAAAGAGAAG
AB074485Malus    106 TACCCCGACTACTACTTTCGTATCACCAACAGCGAGCACAAGGTTGAGCTCAAAGAAAAA
DQ062406Arabido  121 TATCCTGACTACTACTTCCGCATCACCAACAGTGAACACATGACCGACCTCAAGGAGAAG

Myrtus             1 ------------------------------------------------------------
XM_010030619Euc  175 TTCAAGCGCATGTGCGACAAGTCGATGATCAAGAAGCGGTACATGCACCTGACGGAGGAC
JF808008Vitis    166 TTCAAGCGAATGTGTGACAAGTCTATGATCAAGAAACGTTACATGCACTTGACAGAGGAG
AB074485Malus    166 TTCCAGCGCATGTGCGACAAATCTATGATCAAGAAACGTTATATGTACTTGACTGAAGAA
DQ062406Arabido  181 TTCAAGCGCATGTGCGACAAGTCGACAATTCGGAAACGTCACATGCATCTGACGGAGGAA

Myrtus             1 ------------------------------------------------------------
XM_010030619Euc  235 TTCCTGAAGGAGAACCCGGCGATGTGCGCGTACATGGCCAACTCCCTCGACGCCCGCCAG
JF808008Vitis    226 ATCCTGAAAGAGAACCCCAATGTTTGTGAATACATGGCAGCTTCCCTGGATGCTAGGCAG
AB074485Malus    226 ATTTTAAAAGAGAACCCAAGTGTGTGCGAGTACATGGCTCCTTCAATTGATGCAAGGCAG
DQ062406Arabido  241 TTCCTCAAGGAAAACCCACACATGTGTGCTTACATGGCTCCTTCTCTGGACACCAGACAG

Myrtus             1 ------------------------------------------------------------
XM_010030619Euc  295 GACGTGGTGGTCGTGGAGGTCCCGAAGCTGGGCAAGGAGGCCGCCGCGAAGGCCATCAAG
JF808008Vitis    286 GACATGGTGGTGGTGGAGGTACCAAAGCTAGGCAAAGAGGCAGCCGCAAAGGCCATCAAG
AB074485Malus    286 GACATGGTGGTTGTGGAAGTCCCAAAACTTGGCAAAGAGGCTGCCATCAAAGCCATCAAG
DQ062406Arabido  301 GACATCGTGGTGGTCGAAGTCCCTAAGCTAGGCAAAGAAGCGGCAGTGAAGGCCATCAAG

Myrtus             1 ------------------------------------------------------------
XM_010030619Euc  355 GAGTGGGGCCAGTCCAAGTCGAAGATCACTCACCTCATCTTCTGCACCACCTCCGGCGTC
JF808008Vitis    346 GAGTGGGGTCAGCCCAAATCCAAGATCACCCACCTAGTCTTCTGCACCACCAGCGGTGTT
AB074485Malus    346 GAATGGGGACAGCCCAAGTCCAAAATCACCCACTTGGTCTTTTGCACCACCAGCGGTGTC
DQ062406Arabido  361 GAGTGGGGCCAGCCCAAGTCAAAGATCACTCATGTCGTCTTCTGCACTACCTCCGGCGTC

Myrtus             1 ------------------------CTCACCAAGCTCCTCGGCCTCCGCCCCTCTGTTAAG
XM_010030619Euc  415 GACATGCCCGGCGCCGACTACCAGCTCACCAAGCTCCTCGGCCTCCGCCCCTCCGTCAAG
JF808008Vitis    406 GACATGCCCGGTGCTGACTACCAACTCACCAAGCTGCTAGGCCTCCGCCCATCGGTGAAG
AB074485Malus    406 GACATGCCTGGAGCCGACTACCAACTCACCAAGCTCTTGGGCCTCCGCCCCTCCGTCAAG
DQ062406Arabido  421 GACATGCCTGGTGCTGACTACCAGCTCACCAAGCTTCTTGGTCTCCGTCCTTCCGTCAAG

Myrtus            37 CGCTACATGATGTACCAGCAGGGATGCTTCGCCGGCGGCACCGTCCTCCGCCTCGCCAAG
XM_010030619Euc  475 CGCTACATGATGTACCAGCAGGGGTGCTTCGCCGGCGGCACGGTCCTCCGCCTCGCCAAG
JF808008Vitis    466 CGCTTCATGATGTACCAACAGGGCTGCTTTGCTGGTGGCACCGTTCTGCGCCTTGCCAAG
AB074485Malus    466 CGCCTCATGATGTACCAACAAGGGTGCTTCGCCGGTGGGACGGTCCTCCGTTTGGCCAAG
DQ062406Arabido  481 CGTCTCATGATGTACCAGCAAGGTTGCTTCGCCGGCGGTACTGTCCTCCGTATCGCTAAG

Myrtus            97 GACCTCGCCGAGAACAACCGCGGCGCCCGCGTCCTCGTC---------------------
XM_010030619Euc  535 GACCTCGCCGAGAACAACCGCGGCGCCCGCGTCCTCGTCGTGTGCTCCGAAATCACCGCA
JF808008Vitis    526 GACCTTGCCGAGAACAACAAGGGCGCCCGTGTCCTTGTTGTCTGCTCTGAGATCACTGCT
AB074485Malus    526 GACTTGGCCGAAAACAACAAGGGTGCACGTGTTCTTGTTGTGTGCTCTGAGATCACCGCG
DQ062406Arabido  541 GATCTCGCCGAGAACAATCGTGGAGCACGTGTCCTCGTTGTCTGCTCTGAGATCACAGCC
```

```
Myrtus                     ------------------------------------------------------------
XM_010030619Euc  595 GTCACCTTCCGCGGGCCCACCGATACCCACCTCGACTCCCTCGTCGGCCAGGCCCTCTTT
JF808008Vitis    586 GTTACTTTCAGGGGACCTAGTGACACTCACTTGGACAGCCTTGTTGGGCAGGCACTTTTT
AB074485Malus    586 GTTACCTTCCGAGGGCCTAGTGACACCCACCTTGATAGTCTTGTGGGCCAAGCTTTGTTT
DQ062406Arabido  601 GTTACCTTCCGTGGTCCCTCTGACACCCACCTTGACTCCCTCGTCGGTCAGGCTCTTTTC

Myrtus                     ------------------------------------------------------------
XM_010030619Euc  655 GGCGACGGCGCCGCCGCCGTGATCGTCGGCGCCGACCCGGACACCGCCGTGG---AGCGC
JF808008Vitis    646 GGCGATGGGGCGGCCGCTGTTATAGTTGGTTCCGACCCGATCCCAGGGGTGG---AGAAG
AB074485Malus    646 GGCGACGGTGCAGCGGCCGTCATCATTGGTGCGGATCCAGTGCCCGAAGTCG---AGAAG
DQ062406Arabido  661 AGTGATGGCGCCGCCGCACTCATTGTGGGGTCGGACCCTGACACATCTGTCGGAGAGAAA

Myrtus                     ------------------------------------------------------------
XM_010030619Euc  712 CCGCTGTACCAGCTCGTCTCCGCGGCCCAGACCATCCTCCCGGACTCCGACGGCGCCATC
JF808008Vitis    703 CCCATGTTCGAATTGGTTTCAGCAGCCCAGACAATCCTCCCCGACAGCGATGGCGCCATC
AB074485Malus    703 CCCTTGTTTGAATTGGTGTCGGCGGCACAAACCATTCTCCCCGACAGTGATGGGGCTATC
DQ062406Arabido  721 CCCATCTTTGAGATGGTGTCTGCCGCTCAGACCATCCTTCCAGACTCTGATGGTGCCATA

Myrtus                     ------------------------------------------------------------
XM_010030619Euc  772 GACGGCCACCTCCGCGAGGTCGGCCTCACGTTCCACCTCCTCAAGGACGTGCCCGGGCTG
JF808008Vitis    763 GATGGGCATCTCCGGGAAGTGGGGCTGACCTTCCACTTACTTAAGGATGTGCCCGGCCTC
AB074485Malus    763 GACGGACATCTCCGTGAAGTAGGGCTTACATTTCACCTTCTCAAGGATGTTCCCGGACTT
DQ062406Arabido  781 GACGGACATTTGAGGGAAGTTGGTCTCACCTTCCATCTCCTCAAGGATGTTCCCGGCCTC

Myrtus                     ------------------------------------------------------------
XM_010030619Euc  832 ATCTCCAAGAACATCGAGAAGAGCCTCGCGGAGGCCTTCTCCCCGATCGGGATCAGCGAC
JF808008Vitis    823 ATTTCCAAGAACATTGAAAAAAGCCTGAATGAGGCCTTCCAGCCTTTGGGCATCAAGGAC
AB074485Malus    823 ATTTCGAAGAACATCGAAAAGAGCCTTAACGAGGCCTTCAAGCCTATTGGGATTTCGGAC
DQ062406Arabido  841 ATCTCCAAGAACATTGTGAAGAGTCTAGACGAAGCGTTTAAACCTTTGGGGATAAGTGAC

Myrtus                     ------------------------------------------------------------
XM_010030619Euc  892 TGGAACAGCCTCTTCTGGATCGCCCACCCCGGCGGCCCGGCCATCCTGGACCAGGTCGAG
JF808008Vitis    883 TGGAACTCCATTTTCTGGATCGCACACCCTGGTGGGCCTGCAATCTTAGATCAAGTTGAG
AB074485Malus    883 TGGAACTCGCTCTTCTGGATTGCACACCCAGGTGGCCCTGCTATTCTGGACCAAGTAGAG
DQ062406Arabido  901 TGGAACTCCCTCTTCTGGATAGCCCACCCTGGAGGTCCAGCGATCCTAGACCAGGTGGAG

Myrtus                     ------------------------------------------------------------
XM_010030619Euc  952 GCCAAGCTCGGGCTCAAGGAGGAGAAGATGCGAGCCACCCGCCACGTGCTCAGCGAGTAC
JF808008Vitis    943 GAAAAATTAGCCCTAAAGCCCGAGAAGTTACGCTCCACACGACACGTCTTGAGCGAGTGT
AB074485Malus    943 GCCAAGTTGGCACTGAAGCCGGAGAAACTAGAAGCAACAAGGCAAGTGCTGTCGGATTAC
DQ062406Arabido  961 ATAAAGCTAGGACTAAAGGAAGAGAAGATGAGGGCGACACGTCACGTGTTGAGCGAGTAT

Myrtus                     ------------------------------------------------------------
XM_010030619Euc 1012 GGCAACATGTCCAGCGCCTGCGTGCTCTTCATCCTCGACGAGATGCGGCGGAAGTCGGCG
JF808008Vitis   1003 GGTAACATGTCAAGTGCTTGCGTGTTGTTTATTTTGGACGAGATGAGGAGGAAGTCGGCC
AB074485Malus   1003 GGTAACATGTCGAGTGCGTGTGTGCTTTTTATTTTGGACGAGGTCAGGAGGAAGTCCGCC
DQ062406Arabido 1021 GGAAACATGTCGAGCGCGTGCGTTCTCTTCATACTAGACGAGATGAGGAGGAAGTCAGCT

Myrtus                     ------------------------------------------------------------
XM_010030619Euc 1072 GAGGAGGGGCGGCCGACCACCGGCGAGGGCCTGGACTGGGGGGTCCTGTTCGGGTTCGGG
JF808008Vitis   1063 GAGGAAGGGCTGAAGACCACGGGTGAGGGGCCGGAATGGGGCGTTCTTTTCGGTTTCGGA
AB074485Malus   1063 GAGAAAGGACTCAAAACGACCGGGGAGGGACTGGAGTGGGGTGTGCTTTTCGGATTTGGG
DQ062406Arabido 1081 AAGGATGGTGTGGCCACGACAGGAGAAGGGTTGGAGTGGAGTGTCTTGTTTGGTTTCGGA

Myrtus                     ------------------------------------------------------------
XM_010030619Euc 1132 CCGGGGCTGACGGTCGAGACCGTGGTGTTGCACAGCGTCCCCATCGAGGGAGCTCACTGA
JF808008Vitis   1123 CCAGGGCTCACTGTTGAGACCGTGGTGCTCCACAGTGTGTCTACTTGAGGGACTAAAAAA
AB074485Malus   1123 CCCGGCCTCACGGTGGAGACCGTCGTGCTTCACAGCGTGGGT--TTAACGGCTTGA----
DQ062406Arabido 1141 CCAGGTCTCACTGTTGAGACAGTCGTCTTGCACAGCGTTCCTCTCTAA------------
```

```
Myrtus                 ------------------------------------------------------------
XM_010030619Euc 1192 ------------------------------------------------------------
JF808008Vitis   1183 TATCTACTGTTTCCGCTTTACTTGCCCGATTTGTTTTTCCCCTTCTCTTGTTGGTATTGG
AB074485Malus          ------------------------------------------------------------
DQ062406Arabido        ------------------------------------------------------------

Myrtus                 ------------------------------------------------------------
XM_010030619Euc        ------------------------------------------------------------
JF808008Vitis   1243 ACTCTATTTCGAAGTTGTGTGTGGGTAGGCATCAAGGGTATGGTGTAATAGATTGCATTT
AB074485Malus          ------------------------------------------------------------
DQ062406Arabido        ------------------------------------------------------------

Myrtus                 ------------------------------------------------------------
XM_010030619Euc        ------------------------------------------------------------
JF808008Vitis   1303 GAAAAAACAACTTGTATACCAACTTATGATAATAATGATGATATGCTTTTTCTAAAAAAA
AB074485Malus          ------------------------------------------------------------
DQ062406Arabido        ------------------------------------------------------------

Myrtus                 ---------------------------------
XM_010030619Euc        ---------------------------------
JF808008Vitis   1363 AAGAAAAAAAAAAAAAAAAAAAAAAAAAAAAAA
AB074485Malus          ---------------------------------
DQ062406Arabido        ---------------------------------
```

Figure S4. Alignment of the translated amplicon-amino acid sequence from *M. communis* and the full-length CHS sequences from *E. grandis, V. vinifera, M. domestica* and *A. thaliana*. Identical amino acids are shaded in black, and similar amino acids are shaded in grey. The remaining residues are in a white background.

```
Myrtus             1 ------------------------------------------------------------
XP_010028921Euc    1 --MAATKSVEEIRKAQRAEGPATVLAIGTATPNNCISQADYPDYYFRITNSEHMTDLKEK
AEP17003Vitis      1 -----MVTVNEVRNAQRAEGPATVMAIGTATPPNCVDQSTYPDYYFRITNSEHKTELKEK
BAB92996Malus      1 -----MVTVEEVRKAQRAEGPATVMAIGTATPSNCVDQATYPDYYFRITNSEHKVELKEK
AAZ23747Arabido    1 MVMAGASSLDEIRQAQRADGPAGILAIGTANPENHVLQAEYPDYYFRITNSEHMTDLKEK

Myrtus             1 ------------------------------------------------------------
XP_010028921Euc   59 FKRMCDKSMIKKRYMHLTEDFLKENPAMCAYMANSLDARQDVVVVEVPKLGKEAAAKAIK
AEP17003Vitis     56 FKRMCDKSMIKKRYMHLTEEILKENPNVCEYMAASLDARQDMVVVEVPKLGKEAAAKAIK
BAB92996Malus     56 FQRMCDKSMIKKRYMYLTEEILKENPSVCEYMAPSIDARQDMVVVEVPKLGKEAAIKAIK
AAZ23747Arabido   61 FKRMCDKSTIRKRHMHLTEEFLKENPHMCAYMAPSLDTRQDIVVVEVPKLGKEAAVKAIK

Myrtus             1 ----------------------------LTKLLGLRPSVKRYMMYQQGCFAGGTVLRLAK
XP_010028921Euc  119 EWGQSKSKITHLIFCTTSGVDMPGADYQLTKLLGLRPSVKRYMMYQQGCFAGGTVLRLAK
AEP17003Vitis    116 EWGQPKSKITHLVFCTTSGVDMPGADYQLTKLLGLRPSVKRFMMYQQGCFAGGTVLRLAK
BAB92996Malus    116 EWGQPKSKITHLVFCTTSGVDMPGADYQLTKLLGLRPSVKRLMMYQQGCFAGGTVLRLAK
AAZ23747Arabido  121 EWGQPKSKITHVVFCTTSGVDMPGADYQLTKLLGLRPSVKRLMMYQQGCFAGGTVLRIAK

Myrtus            33 DLAENNRGARVLV-----------------------------------------------
XP_010028921Euc  179 DLAENNRGARVLVVCSEITAVTFRGPTDTHLDSLVGQALFGDGAAAVIVGADPDT-AVER
AEP17003Vitis    176 DLAENNKGARVLVVCSEITAVTFRGPSDTHLDSLVGQALFGDGAAAVIVGSDPIP-GVEK
BAB92996Malus    176 DLAENNKGARVLVVCSEITAVTFRGPSDTHLDSLVGQALFGDGAAAVIIGADPVP-EVEK
AAZ23747Arabido  181 DLAENNRGARVLVVCSEITAVTFRGPSDTHLDSLVGQALFSDGAAALIVGSDPDTSVGEK

Myrtus               ------------------------------------------------------------
XP_010028921Euc  238 PLYQLVSAAQTILPDSDGAIDGHLREVGLTFHLLKDVPGLISKNIEKSLAEAFSPIGISD
AEP17003Vitis    235 PMFELVSAAQTILPDSDGAIDGHLREVGLTFHLLKDVPGLISKNIEKSLNEAFQPLGIKD
BAB92996Malus    235 PLFELVSAAQTILPDSDGAIDGHLREVGLTFHLLKDVPGLISKNIEKSLNEAFKPIGISD
AAZ23747Arabido  241 PIFEMVSAAQTILPDSDGAIDGHLREVGLTFHLLKDVPGLISKNIVKSLDEAFKPLGISD

Myrtus               ------------------------------------------------------------
XP_010028921Euc  298 WNSLFWIAHPGGPAILDQVEAKLGLKEEKMRATRHVLSEYGNMSSACVLFILDEMRRKSA
AEP17003Vitis    295 WNSIFWIAHPGGPAILDQVEEKLALKPEKLRSTRHVLSECGNMSSACVLFILDEMRRKSA
BAB92996Malus    295 WNSLFWIAHPGGPAILDQVEAKLALKPEKLEATRQVLSDYGNMSSACVLFILDEVRRKSA
AAZ23747Arabido  301 WNSLFWIAHPGGPAILDQVEIKLGLKEEKMRATRHVLSEYGNMSSACVLFILDEMRRKSA

Myrtus               ---------------------------------------
XP_010028921Euc  358 EEGRPTTGEGLDWGVLFGFGPGLTVETVVLHSVPIEGAH
AEP17003Vitis    355 EEGLKTTGEGPEWGVLFGFGPGLTVETVVLHSVST----
BAB92996Malus    355 EKGLKTTGEGLEWGVLFGFGPGLTVETVVLHSVGLTA--
AAZ23747Arabido  361 KDGVATTGEGLEWSVLFGFGPGLTVETVVLHSVPL----
```

Figure S5. Alignment of the partial nucleotide sequence of *CHI* from *M. communis* and the full-length sequences from *E. grandis, V. vinifera, M. domestica* and *A. thaliana*. Identical nucleotides among the sequences are in a black background, whereas those that are similar among the nucleotide sequences are in grey background. The dashes indicate gaps introduced to improve alignment.

```
Myrtus              1 ------------------------------------------------------------
XM_010069312Euc     1 ATGGGGTCTGAAATGGTTATGGTGGACGAATTCCCTTTCCCTTCGCAGATCACCACTACC
XM_002280122Vit     1 ATGGGAACTGAAATGGTGATGGTGGATGAGATCCCTTTTCCTCCTCAGATCACAACGGCC
XM_008371146        1 ATGGGCACTGAAGTTGTATTGGTGGATGAGGTCCCTTTTCCTTCTAAGATCACAACCACC
NM_120609Arabid     1 ATGGGAACAGAGATGGTCATGGTTCACGAGGTTCCTTTTCCTCCACAGATCATCACTTCC

Myrtus              1 ------------------------------------------------------------
XM_010069312Euc    61 AAGCCTCTCTCACTGCTGGGTCATGGAATCACGGATATGGAGATACATTTCCTACAAATC
XM_002280122Vit    61 AAGCCATTATGCCTTCTGGGTTATGGAATTACTGACATTGAGATTCACTTTCTCCAGATC
XM_008371146       61 AAGCCTTTATCTCTGCTGGGTCAAGGAATTACCGACATAGAGATTCACTTTCTTCAAATT
NM_120609Arabid    61 AAGCCACTCTCTCTTCTGGGCCAAGGGATCACAGACATTGAGATCCACTTTCTTCAAGTG

Myrtus              1 ------------------------------------------------------------
XM_010069312Euc   121 AAATTCACTGCTATTGGAGTGTACTTGGATCCT---GAAATTGTCGGACATCTAAAGCAA
XM_002280122Vit   121 AAGTTCACAGCAATTGGAGTTTACTTGGAACCT---GAAATTGTGGGCCATCTGCAACCA
XM_008371146      121 AAGTTCACAGCAATTGGGGTTTACCTGGATGCT---GAAATTGTGTCACATCTTCAACAA
NM_120609Arabid   121 AAGTTCACTGCGATCGGAGTTTACTTAGATCCTTCAGATGTTAAAACACATCTTGATAAC

Myrtus              1 -----------------------------------TGATGACTTCTTCGAAGCCGTCGCT
XM_010069312Euc   178 TGGAAGGGAAAGCCAGGAAAAGTGTTGGCCGAGGATGACGACTTCTTCGATGCTGTAGCT
XM_002280122Vit   178 TGGAAGGGTAAATCTGGAAAGGAGCTTGCAGAGAATGATGATTTCTTTGAGGCTCTTATT
XM_008371146      178 TGGAAGGCCAAAAAGGCAAATGAACTTGCAGAAGATGATGACTTCTTTGATGCTCTTGTT
NM_120609Arabid   181 TGGAAAGGCAAAACCGGAAAAGAACTCGCCGGCGATGATGACTTCTTCGACGCCCTTGCC

Myrtus             26 TCTGCCCCTGTAGAGAAATTCTTCAGAATCGTCGTGATCAAAGAGATCAAAGGCTCTCAG
XM_010069312Euc   238 TCTGCCCCTGTGGAGAAATTCTTCAGAATTGTCGTGATCAAAGAGATCAAAGGCTCTCAG
XM_002280122Vit   238 TCAGCTCCTGGAGAGAAGTTTTTGAGAATTGTGGTGATCAAGGAGATCAAGGGTTCACAG
XM_008371146      238 TCTGCTCCTGTCGAGAAATTCATTAGGGTTGTGGTGATCAAGGAGATCAAAGGGTCACAA
NM_120609Arabid   241 TCCGCGGAGATGGAGAAGGTTATAAGAGTGGTGGTGATAAAGGAGATAAAAGGAGCTCAG

Myrtus             86 TACGGGGGGCTTCTGGAGAGTGCCGTCAGGGACAGGCTTGCGGCTGAAGACAAGTAC---
XM_010069312Euc   298 TATGGGGGACTTTTGGAGGGTGCCGTCAGGGACAGGCTCGCGGCTGAAGACAAGTACGAG
XM_002280122Vit   298 TACGGGGTGCAGCTAGAGAGTGCAGTCAGGGACCGATTGGCAGCAGATGACAAATATGAA
XM_008371146      298 TATGGAGTGCAGCTTGAGAGTGCAGTGAGGGACAGATTGGCAGCTGATGACAAATATGAG
NM_120609Arabid   301 TACGGAGTGCAGCTAGAGAATACGGTGAGAGATCGTTTGGCTGAGGAGGATAAGTACGAG

Myrtus                ------------------------------------------------------------
XM_010069312Euc   358 GAGGAGGAGGAAGCGGCCCTCGAGAAAGTAGTCGAGTTCTTCCAGTCCAAGTACTGCAAG
XM_002280122Vit   358 GAAGAGGAGGAGGAAGCTTTGGAGAAAGTTGTTGAGTTCTTCCAATCTAAATATTTCAAG
XM_008371146      358 GAAGAGGAGGAGGAAACCTTGGAGAAAGTTGTTGAGTTCTTCCAATCAAAGTACTTCAAG
NM_120609Arabid   361 GAAGAAGAAGAAACTGAGCTCGAGAAGGTCGTTGGCTTTTTCCAGTCCAAGTACTTCAAA

Myrtus                ------------------------------------------------------------
XM_010069312Euc   418 AGTAAATCGGTCATCACATTCCACTTCCCAGCAACTTCCAATACTGCTGAGATTACCTTC
XM_002280122Vit   418 AAAGATTCCATCATTACGTTCCATTTTCCTGCAACTTCATGCACTGCTGAGATTGTATTT
XM_008371146      418 AAGGACTCCGTCATCACATTCCATTTTCCTGCAACTTCTAACACTGCTGAGATTGTGTTT
NM_120609Arabid   421 GCTAACTCCGTTATCACTTACCATTTCTCAGCCAAAGATGGCATTTGCGAGATCGGGTTT

Myrtus                ------------------------------------------------------------
XM_010069312Euc   478 AGCACCGAAGGGAAGGAGGACTCGAAGATGGTGGTGGATAACGGGAACGTGGTCCACATG
XM_002280122Vit   478 GCAACTGAAGGAAAAGAGGAGTCAAAGATCACCGTAGAGAATGCAAACGTTGTTGAGATG
XM_008371146      478 AGCACTGAAGGGAAGGAAGAGTCAAAGATCAAAGTGGAGAATGCAAATGTGGTGGAGAAT
NM_120609Arabid   481 GAGACGGAAGGTAAAGAAGAGGAGAAACTGAAGGTGGAGAATGCGAATGTGGTGGGAATG
```

```
Myrtus                  ------------------------------------------------------------
XM_010069312Euc    538  ATCAAGAAGTGGTACTTGGGAGGGACCAGAGGAGTGTCTGAGACCACCATCACCAGTCTT
XM_002280122Vit    538  ATCAAGAAATGGTACTTGGGTGGAACAAGGGGAGTGTCTCCTACAACCATCTCGGCCTTA
XM_008371146       538  ATCAAGAAATGGTATTTGGGAGGGACGAGAGGGGTGTCTCCGTCGACCATCTCCTCCTTG
NM_120609Arabid    541  ATGCAGAGATGGTATTTGTCCGGAAGTCGTGGAGTCTCACCGTCGACTATTGTCTCCATC

Myrtus                  ---------------------------------
XM_010069312Euc    598  GCCAACACCCTCTCAGAGAAGTTGTCCAAATGA
XM_002280122Vit    598  GCCAACACCCTTGCAACAGAGTTGTCCAAATGA
XM_008371146       598  GCCAACACTCTCTCGGCTGAGTTGACCAAGTGA
NM_120609Arabid    601  GCTGATTCAATCTCCGCAGTTTTAACCTAA---
```

Figure S6. Alignment of the translated amplicon-amino acid sequence from *M. communis* and the full-length CHI sequences from *E. grandis, V. vinifera, M. domestica* and *A. thaliana*. Identical amino acids are shaded in black, and similar amino acids are shaded in grey. The remaining residues are in a white background.

```
Myrtus              1 ------------------------------------------------------------
XP_010067614Euc     1 MGSEMVMVDEFPFPSQITTTKPLSLLGHGITDMEIHFLQIKFTAIGVYLDP-EIVGHLKQ
XP_002280158Vit     1 MGTEMVMVDEIPFPPQITTAKPLCLLGYGITDIEIHFLQIKFTAIGVYLEP-EIVGHLQP
XP_008369368Mal     1 MGTEVVLVDEVPFPSKITTTKPLSLLGQGITDIEIHFLQIKFTAIGVYLDA-EIVSHLQQ
NP_568154Arabid     1 MGTEMVMVHEVPFPPQIITSKPLSLLGQGITDIEIHFLQVKFTAIGVYLDPSDVKTHLDN

Myrtus              1 ------------DAFFEAVASAPVEKFFRIVVIKEIKGSQYGGLLESAVRDRLAAEDKY-
XP_010067614Euc    60 WKGKPGKVLAEDDDFFDAVASAPVEKFFRIVVIKEIKGSQYGGLLEGAVRDRLAAEDKYE
XP_002280158Vit    60 WKGKSGKELAENDDFFEALISAPGEKFLRIVVIKEIKGSQYGVQLESAVRDRLAADDKYE
XP_008369368Mal    60 WKAKKANELAEDDDFFDALVSAPVEKFIRVVVIKEIKGSQYGVQLESAVRDRLAADDKYE
NP_568154Arabid    61 WKGKTGKELAGDDDFFDALASAEMEKVIRVVVIKEIKGAQYGVQLENTVRDRLAEEDKYE

Myrtus                ------------------------------------------------------------
XP_010067614Euc   120 EEEEAALEKVVEFFQSKYCKSKSVITFHFPATSNTAEITFSTEGKEDSKMVVDNGNVVHM
XP_002280158Vit   120 EEEEEALEKVVEFFQSKYFKKDSIITFHFPATSCTAEIVFATEGKEESKITVENANVVEM
XP_008369368Mal   120 EEEEETLEKVVEFFQSKYFKKDSVITFHFPATSNTAEIVFSTEGKEESKIKVENANVVEN
NP_568154Arabid   121 EEEETELEKVVGFFQSKYFKANSVITYHFSAKDGICEIGFETEGKEEEKLKVENANVVGM

Myrtus                -------------------------------
XP_010067614Euc   180 IKKWYLGGTRGVSETTITSLANTLSEKLSK
XP_002280158Vit   180 IKKWYLGGTRGVSPTTISALANTLATELSK
XP_008369368Mal   180 IKKWYLGGTRGVSPSTISSLANTLSAELTK
NP_568154Arabid   181 MQRWYLSGSRGVSPSTIVSIADSISAVLT-
```

Figure S7. Alignment of the partial nucleotide sequence of *DFR* from *M. communis* and the full-length sequences from *E. grandis, V. vinifera, M. domestica* and *A. thaliana*. Identical nucleotides among the sequences are in a black background, whereas those that are similar among the nucleotide sequences are in grey background. The dashes indicate gaps introduced to improve alignment.

```
Myrtus              1 ------------------------------------------------------------
XM_010062668Euc     1 ATGGGATCGGAAGCGCAAGTCGTGTGCGTCACCGGCGCCGCCGGGTTCATCGGCTCGTGG
AY780886Vitis       1 ATGGGTTCACAAAGTGAAACCGTGTGCGTCACCGGTGCCTCCGGTTTCATCGGTTCATGG
AY227728Malus       1 ATGGGATCCGAGTCCGAATCCGTTTGTGTCACCGGGGCGTCCGGTTTCATCGGCTCTTGG
AK221622Arabido     1 ATGGTTAGTCAGAAAGAGACCGTGTGTGTAACCGGCGCTTCGGGTTTCATCGGTTCATGG

Myrtus              1 ------------------------------------------------------------
XM_010062668Euc    61 CTCGTCATGAGGTTGCTCGAGCGCGGGTACACCGTCCGCGCCACCGTCCGTGACCCCAAC
AY780886Vitis      61 CTGGTCATGAGGCTCCTGGAGCGCGGCTACACTGTTCGGGCCACCGTTCGCGATCCAACT
AY227728Malus      61 CTCGTCATGAGACTCCTAGAGCACGGGTACACCGTCCGCGCCACCGTGCGTGACCCCACG
AK221622Arabido    61 CTAGTGATGCGATTACTAGAACGTGGTTACTTTGTTCGTGCCACCGTTCGAGATCCCGGT

Myrtus              1 ------------------------------------------------------------
XM_010062668Euc   121 AACATGAAGAAGGTGAAGCATCTGCTGGACCTGCCCCAGGCGAAAACGCACCTGACTCTC
AY780886Vitis     121 AACGTGAAAAAAGTGAAGCATTTGCTGGACTTGCCCAAAGCGGAGACGCATCTGACTCTC
AY227728Malus     121 AATCAGAAGAAGGTGAAGCATCTGTTGGACTTGCCGAAGGCGGAGACGCACCTGACGCTG
AK221622Arabido   121 AATTTGAAGAAAGTACAACATCTTCTTGATTTGCCAAACGCCAAGACGCAACTCACTTTA

Myrtus              1 ------------------------------------------------------------
XM_010062668Euc   181 TGGAAGGCCGACCTCAACGAAGCAGGAAGCTTCGACGAGGCCATCAATGGCTGCACCGGC
AY780886Vitis     181 TGGAAGGCAGATCTTGCTGATGAAGGAAGTTTCGATGAAGCTATTAAAGGCTGCACCGGC
AY227728Malus     181 TGGAAGGCGGACTTGGCGGATGAGGGAAGCTTTGATGAAGCCATACAAGGCTGCAGCGGA
AK221622Arabido   181 TGGAAGGCTGATTTATCTGAGGAAGGAAGCTACGATGATGCCATAAACGGATGTGACGGT

Myrtus              1 ------------------------------------------------------------
XM_010062668Euc   241 GTCTTCCATGTGGCCACTCCCATGGACTTCGAGTCCAAGGACCCCGAGAATGAAGTGATA
AY780886Vitis     241 GTCTTCCATGTTGCCACACCCATGGATTTTGAATCGAAGGATCCTGAGAATGAAGTGATA
AY227728Malus     241 GTGTTCCATGTTGCTACACCTATGGACTTTGAATCCAAGGACCCCGAGAATGAAGTGATC
AK221622Arabido   241 GTTTTCCACGTGGCAACACCCATGGATTTTGAATCAAAAGATCCTGAGAACGAAGTGATA

Myrtus              1 ------------------------------------------------------------
XM_010062668Euc   301 AAACCGACGGTGGAGGGAGTTCTAGGCATCATGAGAGCATGCGCCAAGGCCAAGACGGTC
AY780886Vitis     301 AAGCCAACTATTGAAGGGATGTTGGGCATAATGAAATCGTGTGCTGCCGCAAAGACTGTC
AY227728Malus     301 AAGCCCACAATCAATGGACTGTTGGACATCCTGAAGGCATGCCAGAAAGCAAAAACAGTT
AK221622Arabido   301 AAGCCGACAGTGAATGGAATGTTGGGGATAATGAAAGCATGTGTTAAGGCAAAGACCGTA

Myrtus              1 ------------------------------------------------------------
XM_010062668Euc   361 CGGCGGCTCGTGTTCACCTCCTCGGCTGGGACTGTCGACGTCCAAGAGCACCGGAAGCCC
AY780886Vitis     361 AGAAGGCTTGTATTCACATCCTCCGCAGGAACTGTGAACATTCAAGAACACCAACTGCCA
AY227728Malus     361 CGAAAGCTTGTGTTCACATCCTCAGCAGGAACCGTGAATGTGGAGGAGCATCAAAAGCCG
AK221622Arabido   361 CGAAGATTCGTATTTACTTCATCTGCCGGAACCGTTAATGTAGAAGAACATCAGAAGAAT

Myrtus              1 ------------------------------------------------------------
XM_010062668Euc   421 GTCTACAGCGAGGAAGACTGGTCCGACATGGACTTCGTGCTCGCCACGAAGATGACCGGA
AY780886Vitis     421 GTGTACGATGAAAGCTGCTGGAGTGATATGGAATTTTGCCGGGCTAAAAAGATGACTGCA
AY227728Malus     421 GTCTACGACGAGTCCAACTGGAGTGACGTTGAATTTTGCCGCTCCGTCAAGATGACTGGT
AK221622Arabido   421 GTCTATGATGAAAATGATTGGAGTGATCTTGAGTTTATCATGTCCAAAAAGATGACAGGA

Myrtus              1 ------------------------------------------------------------
XM_010062668Euc   481 TGGATGTATTTCGTGTCCAAGACGCTGGCAGAGAAAGCAGCTTGGAAATTTGCGGAAGAG
AY780886Vitis     481 TGGATGTACTTTGTCTCCAAGACACTGGCTGAGCAAGCTGCATGGAAGTATGCCAAGGAA
AY227728Malus     481 TGGATGTACTTCGTCTCCAAGACTCTAGCTGAGCAAGCTGCATGGAAATATGCCAAAGAA
AK221622Arabido   481 TGGATGTATTTCGTGTCAAAAACGTTAGCGGAGAAAGCAGCGTGGGATTTCGCCGAAGAG

Myrtus              1 ------------------------------------------------------------
XM_010062668Euc   541 AACAACATTGACCTCATCAGCATCATACCAAGTCTGGTTGTGGGCCCCTTTATCATGTCC
AY780886Vitis     541 AATAACATTGACTTCATCACTATCATACCGACTCTTGTGGTTGGCCCCTTCATAATGTCA
AY227728Malus     541 AACAACATTGATTTCATCACCATTATCCCAACTCTTGTGATTGGGCCATTTCTCATGCCA
```

```
AK221622Arabido    541  AAAGGATTAGATTTCATTAGTATTATTCCAACATTGGTGGTCGGTCCATTCATCACAACG

Myrtus               1  ------------------------------------------------------------
XM_010062668Euc    601  TCAATGCCACCTAGTCTTATCACTGGCCTGTCGCCAATCACAAGAAATGAAGCTCATTAC
AY780886Vitis      601  TCAATGCCTCCAAGCCTCATAACTGCTCTTTCCCCGATCACTGGAAACGAAGCTCATTAT
AY227728Malus      601  TCCATGCCACCAAGCCTCATCACTGGACTTTCGCCGATCTTAAGAAATGAATCACATTAT
AK221622Arabido    601  TCTATGCCGCCTAGCCTTATCACCGCGCTCTCTCCTATCACTCGGAACGAGGCGCATTAC

Myrtus               1  ------------------------------------------------------------
XM_010062668Euc    661  TCCATCATGAAGCAAGGTCACTTCGTCCACACCGACGATCTCTGCGAATCCCACATCTTC
AY780886Vitis      661  TCAATTATACGGCAGGGCCAATTTGTTCACCTGGATGACCTCTGCAATGCTCATATTTAC
AY227728Malus      661  GGCATCATCAAGCAGGGCCAGTATGTTCACTTGGACGACCTCTGCCTTTCTCACATTTAT
AK221622Arabido    661  TCGATCATAAGACAAGGACAGTATGTGCATTTGGACGACTTATGCAACGCTCATATCTTC

Myrtus               1  ------------------------------------------------------------
XM_010062668Euc    721  TTGTTCGAGCACCCGGAAGCCAAGGGACGGTACATCTGCTCCTCCCACGATGCCTCCATC
AY780886Vitis      721  TTGTTTGAGAATCCTAAGGCAGAGGGACGTTACATTTGCTCCTCCCACGATTGTATCATT
AY227728Malus      721  CTTTACGAGCATCCGAAAGCCGAAGGCCGTTACATTTGTTCGTCACATGATGCTACAATT
AK221622Arabido    721  TTATACGAACAAGCAGCCGCCAAGGGACGTTATATTTGTTCCTCTCATGATGCAACCATT

Myrtus               1  ----------------------------------------------TCCCCACGGAGTTC
XM_010062668Euc    781  CTCGAGGTCGCGAATTTGCTCAGGAAGAAATACCCAGAATATGATATTCCCACGGAGTTC
AY780886Vitis      781  CTCGATCTTGCAAAAATGCTTAGAGAAAAATACCCCGAGTATAATATCCCCACAGAGTTC
AY227728Malus      781  CACGAACTTGTAAAAATGCTCAGAGAAAAATACCCCGAATACAATATACCCACAAAGTTC
AK221622Arabido    781  CTTACTATCTCCAAATTTCTCAGGCCAAAATACCCCGAATATAACGTACCTTCAACGTTT

Myrtus              15  GAGGGAGTGGATGAGGGGATGGAGAAGGTTTCTTTCTCTACCAAGAAGCTGTTGGACTTG
XM_010062668Euc    841  GAGGGAGTGGATGAGAATATGCAGAGTGTCACTTTCTGTACCAAGAAGCTGTTGGACTTG
AY780886Vitis      841  AAAGGTGTTGATGAGAACTTGAAGAGTGTCTGTTTCTCCTCCAAGAAGCTGACAGATTTG
AY227728Malus      841  AAGGGCATCGACGACAACTTAGAACCAGTTCATTTTTCTTCAAAGAAGTTGAGGGAGATA
AK221622Arabido    841  GAAGGTGTTGATGAGAATCTAAAGAGCATTGAATTCAGTTCCAAGAAGCTGACGGACATG

Myrtus              75  GGGTTTCAGTACAAGTACACCTTGGAGGACATGTTCGTGGGAGCTGTGGAGACATGCAGA
XM_010062668Euc    901  GGGTTTAAGTACAAGTACACCTTAGAGGACATATTCGTGGGAGCCGTGGAGACATGCAGA
AY780886Vitis      901  GGGTTTGAGTTTAAATACAGCTTAGAGGACATGTTTACTGGAGCTGTGGACACATGCCGG
AY227728Malus      901  GGGTTTGAGTTCAAGTACAGCTTGGAGGACATGTTTGTGGGGGCTGTCGATGCGTGCCGG
AK221622Arabido    901  GGGTTTAACTTCAAGTATAGTCTCGAGGAAATGTTTATTGAATCTATTGAGACATGTCGT

Myrtus             135  GAGAAAGGGCTA------------------------------------------------
XM_010062668Euc    961  GAGAAAGGGCTGCTTCCTCTTTCGCACGAGAA---GCACGTGAAGGGCGCATGCTGA---
AY780886Vitis      961  GCCAAGGGATTGCTTCCCCCTTCACATGAGAA---ACCTGTAGATGGCAAGACCTAG---
AY227728Malus      961  GCAAAGGGCTTGATTCCGATTCCGATTCCGGC---AGAGAAAACTGAGGCTGCTGAGGAG
AK221622Arabido    961  CAAAAGGGTTTTCTCCCGGTTTCATTATCGTACCAATCCATATCGGAGATCAAGACTAAG

Myrtus                  ------------------------------------------------------------
XM_010062668Euc         ------------------------------------------------------------
AY780886Vitis           ------------------------------------------------------------
AY227728Malus     1018  AGCAACCTCGTTGATGTCAAAGCTGGTTAA------------------------------
AK221622Arabido   1021  AATGAAAACATTGACGTCAAAACCGGAGATGGTTTAACCGATGGTATGAAGCCATGTAAC

Myrtus                  ------------------------------------------------------------
XM_010062668Euc         ------------------------------------------------------------
AY780886Vitis           ------------------------------------------------------------
AY227728Malus           ------------------------------------------------------------
AK221622Arabido   1081  AAGACAGAAACGGGGATAACCGGCGAGAGAACCGATGCTCCCATGCTAGCACAACAGATG

Myrtus                  ------
XM_010062668Eu          ------
AY780886Vitis           ------
AY227728Malus           ------
AK221622Arabido   1141  TGTGCC
```

Figure S8. Alignment of the translated amplicon-amino acid sequence from *M. communis* and the full-length DFR sequences from *E. grandis, V. vinifera, M. domestica* and *A. thaliana*. Identical amino acids are shaded in black, and similar amino acids are shaded in grey. The remaining residues are in a white background.

```
AAX12423Vitis       1 MGSQSETVCVTGASGFIGSWLVMRLLERGYTVRATVRDPTNVKKVKHLLDLPKAETHLTL
AAO39816Malus       1 MGSESESVCVTGASGFIGSWLVMRLLEHGYTVRATVRDPTNQKKVKHLLDLPKAETHLTL
BAD95233Arabido     1 MVSQKETVCVTGASGFIGSWLVMRLLERGYFVRATVRDPGNLKKVQHLLDLPNAKTQLTL
Myrtus              1 ------------------------------------------------------------
XP_010060970Euc     1 MGSEAQVVCVTGAAGFIGSWLVMRLLERGYTVRATVRDPNNMKKVKHLLDLPQAKTHLTL

AAX12423Vitis      61 WKADLADEGSFDEAIKGCTGVFHVATPMDFESKDPENEVIKPTIEGMLGIMKSCAAAKTV
AAO39816Malus      61 WKADLADEGSFDEAIQGCSGVFHVATPMDFESKDPENEVIKPTINGLLDILKACQKAKTV
BAD95233Arabido    61 WKADLSEEGSYDDAINGCDGVFHVATPMDFESKDPENEVIKPTVNGMLGIMKACVKAKTV
Myrtus              1 ------------------------------------------------------------
XP_010060970Euc    61 WKADLNEAGSFDEAINGCTGVFHVATPMDFESKDPENEVIKPTVEGVLGIMRACAKAKTV

AAX12423Vitis     121 RRLVFTSSAGTVNIQEHQLPVYDESCWSDMEFCRAKKMTAWMYFVSKTLAEQAAWKYAKE
AAO39816Malus     121 RKLVFTSSAGTVNVEEHQKPVYDESNWSDVEFCRSVKMTGWMYFVSKTLAEQAAWKYAKE
BAD95233Arabido   121 RRFVFTSSAGTVNVEEHQKNVYDENDWSDLEFIMSKKMTGWMYFVSKTLAEKAAWDFAEE
Myrtus              1 ------------------------------------------------------------
XP_010060970Euc   121 RRLVFTSSAGTVDVQEHRKPVYSEEDWSDMDFVLATKMTGWMYFVSKTLAEKAAWKFAEE

AAX12423Vitis     181 NNIDFITIIPTLVVGPFIMSSMPPSLITALSPITGNEAHYSIIRQGQFVHLDDLCNAHIY
AAO39816Malus     181 NNIDFITIIPTLVIGPFLMPSMPPSLITGLSPILRNESHYGIIKQGQYVHLDDLCLSHIY
BAD95233Arabido   181 KGLDFISIIPTLVVGPFITTSMPPSLITALSPITRNEAHYSIIRQGQYVHLDDLCNAHIF
Myrtus              1 ------------------------------------------------------------
XP_010060970Euc   181 NNIDLISIIPSLVVGPFIMSSMPPSLITGLSPITRNEAHYSIMKQGHFVHTDDLCESHIF

AAX12423Vitis     241 LFENPKAEGRYICSSHDCIILDLAKMLREKYPEYNIPTEFKGVDENLKSVCFSSKKLTDL
AAO39816Malus     241 LYEHPKAEGRYICSSHDATIHELVKMLREKYPEYNIPTKFKGIDDNLEPVHFSSKKLREI
BAD95233Arabido   241 LYEQAAAKGRYICSSHDATILTISKFLRPKYPEYNVPSTFEGVDENLKSIEFSSKKLTDM
Myrtus              1 ------------------------------------PTEFEGVDEGMEKVSFSTKKLLDL
XP_010060970Euc   241 LFEHPEAKGRYICSSHDASILEVANLLRKKYPEYDIPTEFEGVDENMQSVTFCTKKLLDL

AAX12423Vitis     301 GFEFKYSLEDMFTGAVDTCRAKGLLPPSHEKPVDGKT-----------------------
AAO39816Malus     301 GFEFKYSLEDMFVGAVDACRAKGLIPIPIPAEKTEAAEESNLVDVKAG------------
BAD95233Arabido   301 GFNFKYSLEEMFIESIETCRQKGFLPVSLSYQSISEIKTKNENIDVKTGDGLTDGMKPCN
Myrtus             25 GFQYKYTLEDMFVGAVETCREKGL------------------------------------
XP_010060970Euc   301 GFKYKYTLEDIFVGAVETCREKGLLPLSHEKHVKGAC-----------------------

AAX12423Vitis         ----------------------
AAO39816Malus         ----------------------
BAD95233Arabido   361 KTETGITGERTDAPMLAQQMCA
Myrtus                ----------------------
XP_010060970Euc       ----------------------
```

Figure S9. Alignment of the partial nucleotide sequence of *LDOX* from *M. communis* and the full-length sequences from *E. grandis, V. vinifera, M. domestica* and *A. thaliana*. Identical nucleotides among the sequences are in a black background, whereas those that are similar among the nucleotide sequences are in grey background. The dashes indicate gaps introduced to improve alignment.

```
Myrtus               1 ------------------------------------------------------------
XM_010055063Euc      1 ATGGTGAG------CGTTGTGGCTGGTAGAGTCGAGAGCTTGTCGAGCAGTGGCATTCAG
X75966Vitis          1 ATGGTGAC------TTCAGTGGCTCCTAGAGTTGAGAGCTTGTCCAGCAGTGGGATCCAG
NM_001328948Mal      1 ATGGTGAGCTCTGATTCAGTGAATTCAAGGGTTGAGACCTTGGCCGGCAGTGGAATCTCA
NM_001036623Ara      1 ------------ATGGTTGCGGTTGAAAGAGTTGAGAGTCTAGCAAAAAGCGGAATCATA

Myrtus               1 ------------------------------------------------------------
XM_010055063Euc     55 TCGATCCCGCAGGAGTATGTGAGGCCGAAGGAGGAGCTCACAAGCATTGGCGACATCTTC
X75966Vitis         55 TCAATCCCCAAAGAGTACATCCGCCCCCAAGAAGAGCTCACCAGCATTGGCAATGTCTTT
NM_001328948Mal     61 ACCATCCCAAAAGAGTACATCAGACCTAAAGATGAGCTTGTAAACATTGGTGACATCTTC
NM_001036623Ara     49 TCGATTCCAAAAGAATATATTCGTCCAAAAGAAGAGCTCGAGAGCATCAACGATGTTTTC

Myrtus               1 ------------------------------------------------------------
XM_010055063Euc    115 GAGGAGGAGAAGAAGCATGAGGGCCCTCAGGTCCCGACCATCGACCTCGAGGACATAGCG
X75966Vitis        115 GAGGAGGAGAAGAAGGATGAAGGGCCTCAGGTTCCAACTATTGACTTGAAGGATATTGAG
NM_001328948Mal    121 GAACAAGAGAAGAACAACGAAGGGCCTCAAGTTCCCACCATCGATTTGAAGGAGATAGAG
NM_001036623Ara    109 CTAGAAGAGAAGAAAGAAGACGGTCCTCAAGTTCCCACAATCGATCTAAAGAACATCGAG

Myrtus               1 ------------------------------------------------------------
XM_010055063Euc    175 TCTAAAGACCCCGTGGT-----GAG-------GGAGAGGTGCCACGAGGAGCTCAGGAAG
X75966Vitis        175 TCTGAGGACGAGGTGGTCCGGCGAGAGATCCGGGAGAGATGCCGGGAGGAGTTGAAGAAA
NM_001328948Mal    181 TCTGATAACGAAAAAGT-----GAG-------AGCAAAATGCAGGGAGGAGTTGAAGAAG
NM_001036623Ara    169 TCAGACGATGAAAAGATCCG------------TGAGAATTGTATTGAGGAGCTGAAAAAG

Myrtus               1 ------------------------------------------------------------
XM_010055063Euc    223 GCTGCCACCGACTGGGGCGTCATGCACCTCGTCAACCATGGGATCCCCAACGACCTGATT
X75966Vitis        235 GCTGCCATGGAGTGGGGTGTGATGCACCTTGTTAACCATGGCATCTCTGATGACCTTATC
NM_001328948Mal    229 GCAGCTGTGGACTGGGGTGTTATGCACCTTGTGAACCATGGCATCTCTGACGAGCTCATG
NM_001036623Ara    217 GCATCTTTGGATTGGGGAGTGATGCATTTGATCAACCATGGAATACCAGCTGATCTAATG

Myrtus               1 ------------------------------------------------------------
XM_010055063Euc    283 GAGCGTGTCAAGAAGGCTGGCGAGGTGTTCTTCAACCTCCCGATCGAGGAGAAGGAGAAG
X75966Vitis        295 AACCGTGTTAAGGTTGCTGGAGAGACCTTTTTCAATCTCCCCATGGAGGAAAAGGAGAAG
NM_001328948Mal    289 GACAAGGTCAGGAAGGCCGGTAAGGCCTTCTTTGACCTTCCCATTGAGCTGAAGGAGAAG
NM_001036623Ara    277 GAGCGTGTCAAGAAAGCCGGAGAAGAGTTTTTCAGTTTGTCTGTGGAAGAGAAGGAGAAG

Myrtus               1 ------------------------------------------------------------
XM_010055063Euc    343 CATGCCAACGACCAGGGCGCCGGGAAGATCCAGGGCTACGGGAGCAAGCTTGCAAACAAT
X75966Vitis        355 TATGCTAATGACCAGGCCTCCGGCAAGATCGCTGGCTATGGCAGCAAGCTTGCCAACAAT
NM_001328948Mal    349 TATGCCAATGACCAGGCCTCTGGTAAGATTCAAGGCTATGGAAGCAAGCTTGCAAACAAT
NM_001036623Ara    337 TATGCAAACGATCAAGCCACTGGAAAGATTCAAGGCTATGGAAGTAAATTGGCTAACAAC

Myrtus               1 ------------------------------------------------------------
XM_010055063Euc    403 GCCAGCGGGCAGCTCGAGTGGGAGGACTACTTCTTCCACCTCGTTTACCCTGAGGACAAG
X75966Vitis        415 GCTAGTGGACAGCTTGAGTGGGAGGACTACTTCTTCCACCTCATCTTCCCTGAAGACAAG
NM_001328948Mal    409 GCATCTGGGCAGCTTGAGTGGGAGGACTACTTCTTCCACTGTGTATACCCAGAGGACAAG
NM_001036623Ara    397 GCGAGTGGACAATTGGAATGGGAAGATTACTTCTTTCATCTTGCGTATCCTGAAGAGAAG

Myrtus               1 ------------------------------------------------------------
XM_010055063Euc    463 CGTGACTTGTCCATCTGGCCCAAGCACCCGAGCGATTACACAGCTGCGACGAGCGAGTAC
X75966Vitis        475 CGCGATATGACCATCTGGCCTAAGACACCAAGCGACTACGTTCCAGCAACCTGTGAGTAC
NM_001328948Mal    469 CGTGACTTGTCTATTTGGCCTCAAACACCTGCTGATTACATTGAGGCAACCGCCGAGTAT
NM_001036623Ara    457 AGAGATCTATCAATTTGGCCTAAGACACCAAGTGATTACATAGAAGCAACGAGTGAGTAC

Myrtus               1 ------------------------------------------------------------
XM_010055063Euc    523 GCCAAGCTGCTGAGACAGCTCGCGACCAAGATCCTGTCAGCACTCTCAATCAGTCTAGGA
X75966Vitis        535 TCGGTGAAACTTAGGAGCCTGGCAACCAAGATACTATCGGTGCTATCGCTTGGGTTGGGA
NM_001328948Mal    529 GCTAAGCAACTGAGGGAGCTAGCAACCAAGGTACTGAAAGTTCTGTCACTTGGCTTGGGA
NM_001036623Ara    517 GCGAAGTGTCTTCGTTTGCTAGCGACTAAAGTCTTCAAGGCTCTCTCTGTCGGTCTAGGT
Myrtus               1 ------------------------------------------------------------
```

```
XM_010055063Euc  583 CTCGAAGAAGGGAGGTTGGAGAAGGAAGTCGGCGGGCTTGAGGAAATGCTGCTCCAGATG
X75966Vitis      595 TTGGAAGAAGGGAGACTAGAAAAGGAAGTTGGTGGGATGGAAGAGCTACTACTCCAAAAG
NM_001328948Mal  589 TTGGATGAAGGGAGGCTGGAGAAAGAAGTTGGTGGACTTGAAGAGCTCCTCTTGCAAATG
NM_001036623Ara  577 TTAGAGCCTGACCGTCTAGAGAAAGAAGTTGGTGGTTTAGAAGAGCTTCTTCTACAAATG

Myrtus             1 ------AACTACTACCCGAAATGCCCCCAACCGGAGCTAGCCCTAGGGGTCGAGGCTCAC
XM_010055063Euc  643 AAGATCAACTACTACCCGAAATGCCCCCAACCAGAGCTAGCCCTAGGAGTCGAGGCTCAC
X75966Vitis      655 AAGATCAACTACTACCCCAAGTGTCCCCAGCCTGAATTGGCTCTCGGGGTGGAAGCTCAC
NM_001328948Mal  649 AAAATCAACTACTACCCAAAATGCCCTCAGCCGGAGCTTGCACTTGGTGTTGAAGCTCAC
NM_001036623Ara  637 AAGATAAATTACTATCCAAAATGTCCTCAGCCTGAGCTAGCACTCGGTGTGGAAGCTCAC

Myrtus            55 ACGGACGTGAGCGCTCTGACTTTCATCCTCCACAACATGGTCCCCGGACTCCAGCTCTTC
XM_010055063Euc  703 ACGGATGTGAGTGCCCTGACTTTTATCCTCCACAACATGGTCCCTGGACTTCAGCTCTTC
X75966Vitis      715 ACTGACGTGAGCGCTCTCACCTTCATCCTCCACAACATGGTACCCGGCCTGCAACTTTTC
NM_001328948Mal  709 ACTGACGTGAGTGCACTCACCTTCATCCTCCACAACATGGTTCCTGGCCTGCAGCTTTTC
NM_001036623Ara  697 ACCGATGTAAGCGCTTTAACTTTCATTCTACACAACATGGTTCCGGGTTTGCAGCTTTTC

Myrtus           115 TACGAGGGCAAGTGGGTCACGGCCAAATGCGTCCCCAACTCCATCATTATGCACATTGGG
XM_010055063Euc  763 TACGAGGGCAAGTGGGTCACGGCCAAATGTGTCCCCAACTCCATCATCATGCACATTGGG
X75966Vitis      775 TATGAGGGCAAGTGGGTGACAGCCAAGTGTGTCCCCAACTCCATCATCATGCACATTGGA
NM_001328948Mal  769 TATGAAGGAAAGTGGGTCACTGCCAAGTGCGTTCCAAATTCCATCGTCATGCACATTGGG
NM_001036623Ara  757 TACGAGGGCAAATGGGTCACTGCAAAATGTGTTCCTGATTCGATTGTGATGCACATTGGG

Myrtus           175 GACACCATTGAGATC---------------------------------------------
XM_010055063Euc  823 GACACCATTGAGATCCTGAGCAATCGCAAGTACAAGAGCATCCTCCACCGCGGGCTGGTC
X75966Vitis      835 GACACCATAGAGATTCTCAGCAATGGTAAGTACAAGAGTATTCTTCACAGGGGACTGGTC
NM_001328948Mal  829 GACACACTTGAGATTTTGAGCAATGGGAAGTACAAAAGTATACTCCACAGGGGCATGGTG
NM_001036623Ara  817 GATACTTTGGAGATTCTTAGTAATGGGAAGTATAAGAGTATACTTCATCGTGGGTTGGTG

Myrtus               ------------------------------------------------------------
XM_010055063Euc  883 AACAAGGAGAAGGTGAGGATCTCGTGGGCCGTCTTCTGCGAGCCTCCAAAGGAGAAGATC
X75966Vitis      895 AACAAGGAGAAGGTGAGGATTTCATGGGCAGTTTTCTGCGAGCCGCCTAAGGAGAAGATC
NM_001328948Mal  889 AACAAGGAAAAGGTGAGGATTTCATGGGCAGTTTTCTGTGAGCCACCAAAGGAGAAGATC
NM_001036623Ara  877 AATAAGGAGAAGGTTAGGATTTCTTGGGCTGTGTTTTGTGAACCCCCAAAGGATAAGATT

Myrtus               ------------------------------------------------------------
XM_010055063Euc  943 ATCCTCAAGCCGC--TGCCTGAGATGGTGACTGAGGCAGAGCCTGCGCTGTTCCCTCCCC
X75966Vitis      955 ATCCTGAAGGCGCACTGCCAGAGACGGTGTCTGAGACTGAGCCACCACTCTTCCCACCTC
NM_001328948Mal  949 ATCCTTAAGCCAC--TGCCGGAGACCGTGTCCGAGGAGGAGCCGGCAATGTTCCCACCAC
NM_001036623Ara  937 GTTCTTAAGCCGT--TGCCGGAGATGGTGAGTGTTGAGTCTCCGGCTAAGTTTCCTCCAC

Myrtus               ------------------------------------------------------------
XM_010055063Euc 1001 GCACATTTGCTCAGCACATCGAGCACAAGCTCTTCAGGAAGAACCAGGCCGCA------T
X75966Vitis     1015 GCACCTTTTCCCAACATATTCAGCACAAGCTCTTCAGGAAGACCCAGGAGGCTCTACTCT
NM_001328948Mal 1007 GAACTTTTGCTGAGCACATTCAACACAAGTTGTTCAGGAAGAGTCAAGGGGCTTTGCTCC
NM_001036623Ara  995 GGACTTTTGCTCAACATATTGAGCATAAGTTGTTTGGGAAGGAACAAGAGGAATTGGTAT

Myrtus               -----------------
XM_010055063Euc 1055 CGAAATGA---------
X75966Vitis     1075 CCAAATGAGCTGTAG--
NM_001328948Mal 1067 CCAAGTGA---------
NM_001036623Ara 1055 CCGAGAAAAATGATTAA
```

Figure S10. Alignment of the translated amplicon-amino acid sequence from *M. communis* and the full-length LDOX sequences from *E. grandis, V. vinifera, M. domestica* and *A. thaliana*. Identical amino acids are shaded in black, and similar amino acids are shaded in grey. The remaining residues are in a white background.

```
NP_001031700Ara     1 ----MVAVERVESLAKSGIISIPKEYIRPKEELESINDVFLEEKKEDGPQVPTIDLKNIE
NP_001315877Mal     1 MVSSDSVNSRVETLAGSGISTIPKEYIRPKDELVNIGDIFEQEKNNEGPQVPTIDLKEIE
XP_010053365Euc     1 --MVSVVAGRVESLSSSGIQSIPQEYVRPKEELTSIGDIFEEEKKHEGPQVPTIDLEDIA
Myrtus              1 ------------------------------------------------------------
CAA53580Vitis       1 --MVTSVAPRVESLSSSGIQSIPKEYIRPQEELTSIGNVFEEEKKDEGPQVPTIDLKDIE

NP_001031700Ara    57 SDDEKIR----ENCIEELKKASLDWGVMHLINHGIPADLMERVKKAGEEFFSLSVEEKEK
NP_001315877Mal    61 SDNEKVR----AKCREELKKAAVDWGVMHLVNHGISDELMDKVRKAGKAFFDLPIELKEK
XP_010053365Euc    59 SKDPVVR----ERCHEELRKAATDWGVMHLVNHGIPNDLIERVKKAGEVFFNLPIEEKEK
Myrtus              1 ------------------------------------------------------------
CAA53580Vitis      59 SEDEVVRREIRERCREELKKAAMEWGVMHLVNHGISDDLINRVKVAGETFFNLPMEEKEK

NP_001031700Ara   113 YANDQATGKIQGYGSKLANNASGQLEWEDYFFHLAYPEEKRDLSIWPKTPSDYIEATSEY
NP_001315877Mal   117 YANDQASGKIQGYGSKLANNASGQLEWEDYFFHCVYPEDKRDLSIWPQTPADYIEATAEY
XP_010053365Euc   115 HANDQGAGKIQGYGSKLANNASGQLEWEDYFFHLVYPEDKRDLSIWPKHPSDYTAATSEY
Myrtus              1 ------------------------------------------------------------
CAA53580Vitis     119 YANDQASGKIAGYGSKLANNASGQLEWEDYFFHLIFPEDKRDMTIWPKTPSDYVPATCEY

NP_001031700Ara   173 AKCLRLLATKVFKALSVGLGLEPDRLEKEVGGLEELLLQMKINYYPKCPQPELALGVEAH
NP_001315877Mal   177 AKQLRELATKVLKVLSLGLGLDEGRLEKEVGGLEELLLQMKINYYPKCPQPELALGVEAH
XP_010053365Euc   175 AKLLRQLATKILSALSISLGLEEGRLEKEVGGLEEMLLQMKINYYPKCPQPELALGVEAH
Myrtus              1 ------------------------------------------NYYPKCPQPELALGVEAH
CAA53580Vitis     179 SVKLRSLATKILSVLSLGLGLEEGRLEKEVGGMEELLLQKKINYYPKCPQPELALGVEAH

NP_001031700Ara   233 TDVSALTFILHNMVPGLQLFYEGKWVTAKCVPDSIVMHIGDTLEILSNGKYKSILHRGLV
NP_001315877Mal   237 TDVSALTFILHNMVPGLQLFYEGKWVTAKCVPNSIVMHIGDTLEILSNGKYKSILHRGMV
XP_010053365Euc   235 TDVSALTFILHNMVPGLQLFYEGKWVTAKCVPNSIIMHIGDTIEILSNRKYKSILHRGLV
Myrtus             19 TDVSALTFILHNMVPGLQLFYEGKWVTAKCVPNSIIMHIGDTIEI---------------
CAA53580Vitis     239 TDVSALTFILHNMVPGLQLFYEGKWVTAKCVPNSIIMHIGDTIEILSNGKYKSILHRGLV

NP_001031700Ara   293 NKEKVRISWAVFCEPPKDKIVLKPLPEMVSVESPAKFPPRTFAQHIEHKLFGKEQEELVS
NP_001315877Mal   297 NKEKVRISWAVFCEPPKEKIILKPLPETVSEEEPAMFPPRTFAEHIQHKLFRKSQGALLP
XP_010053365Euc   295 NKEKVRISWAVFCEPPKEKIILKPLPEMVTEAEPALFPPRTFAQHIEHKLFRKNQAASK-
Myrtus                ------------------------------------------------------------
CAA53580Vitis     299 NKEKVRISWAVFCEPPKEKIILKAHCQRRCLRLSHHSSHLAPFPNIFSTSSSGRPRRLY

NP_001031700Ara   353 EKND
NP_001315877Mal   357 K---
XP_010053365Euc       ----
Myrtus                ----
CAA53580Vitis     359 PNEL
```

Figure S11. Alignment of the partial nucleotide sequence of *UFGT* from *M. communis* and the full-length sequences from *E. grandis, V. vinifera, M. domestica* and *A. thaliana*. Identical nucleotides among the sequences are in a black background, whereas those that are similar among the nucleotide sequences are in grey background. The dashes indicate gaps introduced to improve alignment.

```
XM_010064884Euc     1 ATGTCTTCTTCGCCTTCCGACAGCGTTCAGTGTCTCGACCCACCTCGTGAAATGAGGACC
XM_010064907Euc     1 ---------------------------------------------------ATGAGCACC
Myrtus              1 ------------------------------------------------------------
AB047093Vitis       1 ------------------------------------------------------------
AY072325Arabido     1 ---------------------------------------------------ATGACCAAA
AF117267Malus       1 --------ATGGCAGCGCCGCTGCCCATCGAAATCGAACCATCATCAACTAATGGTCAAC

XM_010064884Euc    61 TCA--GGCAGCGGCAGCGGCAGCCCCCACGTCGCCGTCCTGGCCTTCCCCTTCGGAACCC
XM_010064907Euc    10 --------ACTGGCAGAGGCAGCCCCCACGTTGCCGTCCTGGCGTTCCCATTCTCGACCC
Myrtus              1 ------------------------------------------------------------
AB047093Vitis       1 ATG--TCTCAAACCACCACCAACCCCATGTGGCCGTCCTGGCCTTCCCCTTCTCCACCC
AY072325Arabido    10 CCC--TCCGACCCAACCAGAGACTCCCACGTGGCAGTTCTCGCTTTTCCTTTCGGCACTC
AF117267Malus      53 CCCATCTCGCCGACGCCTACAACCGTCACGTGGCTGTCGTAGCCTTCCCTTTCACTAGCC

XM_010064884Euc   119 ACGCCGCTCCCCTCCTCGCCGTCATCCGCCGCCTGGCCTCCGCCACCCCCGACACGCTCT
XM_010064907Euc    62 ATGCGGCTCCCCTCCTTGCTGTGATCCGCCGCCTGGCCACCGCCACCCCAGACACGCTCT
Myrtus              1 ------------------------------------------------------------
AB047093Vitis      59 ATGCAGCCCCCCTCCTTGCCGTCGTTCGCCGCCTTGCTGCCGCTGCCCCTCATGCAGTCT
AY072325Arabido    68 ATGCAGCTCCTCTCCTCACCGTCACGCGCCGCCTCGCCTCCGCCTCTCCTTCCACCGTCT
AF117267Malus     113 ATGCAAGCGCCTTGCTTGAGACCGTGCGCCGCCTAGCCACCGCCCTTCCAAACACTCTCT

XM_010064884Euc   179 ACTCTTTCTTCAGCACTGCCGATTCCATCGCCTC--CGTCTTCT-CGGCT--TACCGCCA
XM_010064907Euc   122 ACTCTTTCTACAGCACTGCCAAGTCCATAGCCTC--CATCTTCT-CGGCT--TCCAACAA
Myrtus              1 ------------------------------------------------------------
AB047093Vitis     119 TCTCCTTCTTCAGCACCAGCCAATCCAACGCCTC--CATCTTCCACGACT--CCATGCAT
AY072325Arabido   128 TCTCTTTCTTCAACACCGCACAATCCAACTCTTCGTTATTTTCCTCCGGTGACGAAGCAG
AF117267Malus     173 TCTCGTTCTTCAGCACTTCAAAATCCAACAGCTCTCTCTTTTCCAACAACAGCATTG-AT

XM_010064884Euc   234 CTTGCCCAACTTGAGAGCT-----TACCACGTGGAGCCGGAGTACCGGAGGGTCATGTG
XM_010064907Euc   177 CTTGCCCAACGTGATAGGG-----TACGACGTGGAGATGGAGTGCCAGAGGGATATGTG
Myrtus              1 ------------------------------------------------------------
AB047093Vitis     175 ACCATGCAATGTAATATCAAGTCCTATGATGTGTCCGACGGTGTGCCTGAGGGGTATGTG
AY072325Arabido   188 ATCGTCCGGCG-AACATCAGAGTATACGATATTGCCGACGGTGTTCCGGAGGGATACGTG
AF117267Malus     232 AACATGCCGCGTAACATAAGGGTGTACGATGTGGCTGACGGGGTGCCGGAGGGGTACGTT

XM_010064884Euc   289 CGGGTGGGCAAGCCAGAAGAGGACATCGAGCTGTTCCTCAGGGCCGCCCCAGCCAGCTTC
XM_010064907Euc   232 TGGGTGGGGAAACCACAGGAGGACATCGAGTTGTTCCTGAGGGCGGCCCCAGCCAACTTC
Myrtus              1 ---------------------------------------------------------TTC
AB047093Vitis     235 TTCGCCGGGCGGCCCCAGGAGGATATTGAGCTGTTCATGAGGGCTGCGCCGGAGAGCTTT
AY072325Arabido   247 TTTAGCGGGAGACCACAGGAGGCGATCGAGCTGTTTCTTCAAGCTGCGCCGGAGAATTTC
AF117267Malus     292 TTCGTGGGCAAGCCGCAGGAGGACATAGAGCTCTTCATGAATGCCGCACCGGAAAACATC

XM_010064884Euc   349 AGAAAGGGGATGGAGCGGGCAGTGGCCGAGACGGGGAGGAGGGTGAGCTGCTTGGTCACC
XM_010064907Euc   292 AGGAAGGGGATGGAAGAGGCGGTGGCCAAGACGGGCAGGAGGGTGACCTGCTTGGTCACC
Myrtus              4 AGGAAGGGGATAGAGGAGGCGGTGGCCAAGACGGGGAGGGGGGTGAGCTGCTTGGTCACT
AB047093Vitis     295 AGGCAGGGGATGGTGATGGCTGTGGCCGAGACAGGGCGGCCAGTGAGCTGCCTGGTGGCT
AY072325Arabido   307 CGGAGAGAAATCGCGAAGGCGGAGACGGAGGTTGGTACGGAAGTGAAATGTTTGATGACT
AF117267Malus     352 CGGAGGAGCTTAGACGCTTCCGTGGCGGACATCGGGAAGCAGATCAGCTGCTTGATCACC

XM_010064884Euc   409 GACGCCTTCTTCTGGTTCGCCGCGGATATGGCCGCGGAGATGGAGTTGCCGTGGGTGGCC
XM_010064907Euc   352 GACGCCTTCTTCTGGTTCGCCGCGGATATGGCCGCGAAGATGGAGTTGCCGTGGGTGGCC
Myrtus             64 GATGCCTTCTTCTGTTTCTGCGCGGATATGGCAGCGGAGATGGAATTGCCGTGGGTGGCC
AB047093Vitis     355 GACGCATTCATTTGGTTTGCTGCAGATATGGCAGCAGAGATGGGGTGGCTTGGCTGCCG
AY072325Arabido   367 GATGCGTTCTTCTGGTTCGCGGCTGATATGGCGACGGAGATAAATGCGTCGTGGATTGCG
AF117267Malus     412 GACGCCTTCCTTTGGTTTGGAGTCCACTTGGCTGACGAGTTGGGAGTGCCTTGGGTCACT
```

```
XM_010064884Euc  469 TTCTGGACTGCTGGCCCTGCCTCTCTCTCTGCCCATCTCTACACCGAGCATATCAGGCAG
XM_010064907Euc  412 TTCTGGACTGCTGGCCCTGCCTCTCTCTCTGTCCATCTCTACACCGAGCATATCAGGCAG
Myrtus           124 TTCTGGCCTGCTGGCCCTGCCTCTCTCTCTGCCCATCTCTACACCGAGCATATCAGGCAG
AB047093Vitis    415 TTTTGGACTGCAGGGCCTAACTCACTCTCCACCCATGTTTACACTGATGAAATCAGAGAA
AY072325Arabido  427 TTTTGGACCGCCGGAGCAAACTCACTCTCTGCTCATCTCTACACAGATCTCATCAGAGAA
AF117267Malus    472 TTCTGGATCTCCGGACTCAAATCCCTCTCCGTTCATGTGCATACTGATCTCATCCGCGAC

XM_010064884Euc  529 ACTCTCGGCGCCAGCCTAGGAATGGACGGGCGAGCAGATGA-------GACCCTGCAATT
XM_010064907Euc  472 ACTCTTGGCGCCAGCCGAGGAATGGACGGGCGAGCAGATGA-------GACCCTGCAATT
Myrtus           184 ACT---------------------------------------------------------
AB047093Vitis    475 AAGATTGGAGTT---TCAGGCATTCAAGGCCGTGAAGACGA-------GCTGCTCAATTT
AY072325Arabido  487 ACCATCGGTGTCA---AAGAAGTAGGTGAGCGTATGGAGGA-------GACAATAGGGGT
AF117267Malus    532 ACTATTGGAACT---CAAGGCATTACAGGTCGTGAAAACGACCTCATCGTCGACAAAAAT

XM_010064884Euc  582 -----CATCCCGGGAATGTCAAAAATACGCATCAGGGACTTGCCAGAAGGAGTCGTGTTC
XM_010064907Euc  525 -----CATCCCGGGAATGTCAAAAATACGCATCAGGGACTTGCCAGAAGGAGTCGTGTTC
Myrtus               ------------------------------------------------------------
AB047093Vitis    525 -----CATTCCCGGAATGTCTAAAGTACGTTTTCGCGACCTGCAGGAAGGCATCGTGTTC
AY072325Arabido  537 -----TATCTCAGGAATGGAGAAGATCAGAGTCAAAGATACACCAGAAGGAGTTGTGTTT
AF117267Malus    589 GTTAACATCCAAGGTCTCTCCAATGTACGAATCAAAGACTTAGCGGAAGGAGTCATTTTC

XM_010064884Euc  637 GGGAACTTGGACTCACTCTTCTCCCGCATGCTTTGCGACATGGGCAGGGCATTGCCGAGA
XM_010064907Euc  580 GGGAACTTGGACTCACTCTTCTCCCGCATGCTTTGCGACATGGGCAGGGCCTTGCCGAGA
Myrtus               ------------------------------------------------------------
AB047093Vitis    580 GGAAACCTAAACTCGCTCTTCTCACGCATGCTCCATCGGATGGGCCAAGTGCTACCTAAG
AY072325Arabido  592 GGGAATTTAGACTCTGTTTTCTCAAAGATGCTTCATCAAATGGGTCTTGCTTTGCCTCGT
AF117267Malus    649 GGAAACTTGGACTCGGTAATTTCCGGCATGCTACTTCAGATGGGACGGCTCCTCCCCCGT

XM_010064884Euc  697 GCAGCTGCAGTTTTCCTGAACAGCTTCGAGGAGTTGGACCCAACCATAACCGCTGATCTC
XM_010064907Euc  640 GCAGCTGCAGTTTTCCTGAACAGCTTCGAGGAGTTGGACCCAACCATAACCGCTGATCTC
Myrtus               ------------------------------------------------------------
AB047093Vitis    640 GCGACTGCAGTTTTCATAAACTCCTTCGAGGAGCTCGACGATTCCCTAACCAATGATCTC
AY072325Arabido  652 GCCACTGCTGTTTTCATCAATTCTTTTGAAGATTTGGATCCTACATTGACGAATAACCTC
AF117267Malus    709 GCCACCGCAGTTTTCATGAACGGCTTCGAAGAATTGGAACTCCCCATACCAAACGACCTA

XM_010064884Euc  757 AAGTCCAAGCTCAACAACTTCCTCAACGTGGGTCCCTTCAACCTCATAGCCACT------
XM_010064907Euc  700 AAGTCCAAGCTCAACAACTTCCTCAACGTGGGTCCCTTCAACCTCATAGCCACT------
Myrtus               ------------------------------------------------------------
AB047093Vitis    700 AAATCCAAGCTCAAGACGTACCTCAATATCGGTCCATTTAACCTAATAACCCCA------
AY072325Arabido  712 AGATCGAGATTTAAACGATATCTGAACATCGGTCCTCTCGGGTTATTATCTTCTACATTG
AF117267Malus    769 AAGTCCAAAGTCAACAAACTCCTCAACGTAGGACCTTCCAACGTAGCATCCCCG------

XM_010064884Euc  811 CAGCCGCGTGCTCCCGATCAAAGCAAGTGCATGTCGTGGCTGGACGGACAA---GGGCGG
XM_010064907Euc  754 CAGCCGCGTGCTCCCGATCAAAGCAAGTGCATGTCGTGGCTGGACGGACAA---GGGCGG
Myrtus               ------------------------------------------------------------
AB047093Vitis    754 CCGCCGGTTATACCCAACACAACCGGCTGCCTCCAATGGCTCAAAGAAAGA---AAACCC
AY072325Arabido  772 CAACAACTAGTGCAAGATCCTCACGGTTGTTTGGCTTGGATGGAGAAGAGA---TCTTCT
AF117267Malus    823 CTGCCACCGCTGCCGCCATCAGATGCTTGCTTGTCATGGCTAGACAAGCAACAGGCTCCA

XM_010064884Euc  868 GCCTCCGTTGCCTACGTTAGCTTTGGGTCAGTGACGGTGCCTCCTCGCGAGGAGATTGTG
XM_010064907Euc  811 GCCTCCGTTGCCTACATTAGCTTTGGGTCGGTGACGGTGCCTCCTCGTGAGGAGATTGTG
Myrtus               ------------------------------------------------------------
AB047093Vitis    811 ACCTCGGTCGTGTACATTAGCTTTGGCACCGTCACGACACCACCCCCAGCCGAGCTTGTA
AY072325Arabido  829 GGTTCTGTGGCGTACATTAGCTTTGGTACGGTCATGACACCGCCTCCTGGAGAGCTTGCG
AF117267Malus    883 TCCTCCGTCGTGTACATAAGCTTCGGGACAGTGGCGAGCCCAGCGGAGAAGGAGCAGATG

XM_010064884Euc  928 GAACTAGCAGAGGCCTTGGAAGCGAGCCGAGTGCCATTCATATGGTCTCTCAAGGACCAT
XM_010064907Euc  871 GAACTAGCAGAGGCCTTGGAAGCGAGCCGAGTGCCATTCATATGGTCTCTCAAGGACCAT
Myrtus               ------------------------------------------------------------
AB047093Vitis    871 GCCCTAGCTGAGGCACTGGAGGCAAGCCGGGTACCGTTTATATGGTCCCTAAGGGACAAG
AY072325Arabido  889 GCGATAGCAGAAGGGTTGGAATCGAGTAAAGTGCCGTTTGTTTGGTCGCTTAAGGAGAAG
AF117267Malus    943 GCAATAGCGGAGGCCCTGGAAGCCACCGGAGCACCCTTCTTGTGGTCTATCAAGGACAGC
```

```
XM_010064884Euc  988 CTAAGAGAGAATCTGCCAGAGGGGTTTCTTGAGAGGAAC------GAGACAAGAGGGATG
XM_010064907Euc  931 TTAATAGAGACTCTTCCAGAGGGCTTTCTAAAGGGGACC------GAGACAAGAGGGATG
Myrtus               ------------------------------------------------------------
AB047093Vitis    931 GCAAGGGTGCATTTGCCAGAAGGTTTCTTGGAGAAGACC------AGAGGGTACGGAATG
AY072325Arabido  949 AGCTTGGTTCAGTTACCAAAAGGGTTTTTGGATAGGACA------AGAGAGCAAGGGATA
AF117267Malus   1003 TGCAAGACACCGTTGCTGAACGAGTTCTTGACAAAAACATTGTCAAAGCTGAACGGGATG

XM_010064884Euc 1042 ATGGTGGCATGGGCACCCCAGGAAGAGATACTGAAGCATGCTGCGATCGGCGCATTTATA
XM_010064907Euc  985 GTGGTGGCGTGGGCACCCCAGGAAGAGATACTGAGGCATGATGCAGTCGGCGCATTTATA
Myrtus               ------------------------------------------------------------
AB047093Vitis    985 GTGGTTCCATGGGCTCCTCAGGCGGAGGTCCTAGCACATGAGGCAGTTGGGGCTTTTGTA
AY072325Arabido 1003 GTGGTTCCATGGGCACCGCAAGTGGAACTGCTGAAACACGAAGCAACGGGTGTGTTTGTG
AF117267Malus   1063 GTGGTGCCGTGGGCTCCACAGCCGCATGTACTGGCCCACGATTCGGTCGGAGCCTTCGT

XM_010064884Euc 1102 ACGCACTGCGGTTGGAACTCTTTGCTGGAGAGCATAGGGGGTGGAGGGGTGGCCATGATG
XM_010064907Euc 1045 ACGCACTGCGGTTGGAACTCATTACTAGAGAGCATTGGGGGTGGAGGAGTGGCCATGATT
Myrtus               ------------------------------------------------------------
AB047093Vitis   1045 ACACATTGTGGATGGAACTCATTGTGGGAAAGCGT---GGCCGGTGGGGTACCCTTGATT
AY072325Arabido 1063 ACGCATTGTGGATGGAACTCGGTGTTGGAGAGTGT---ATCGGGTGGTGTACCGATGATT
AF117267Malus   1123 TCGCATTGCGGCTGGAACTCGATAATGGAGACTAT---AGCAGGACGGGTGCCCATGATT

XM_010064884Euc 1162 TGTAGGCCGTTCTTTGGGGACCAGAGGTTGAATGGGCGGATGATGGAGGAGGTGTGGGGG
XM_010064907Euc 1105 TGTAGGCCATTCTTTGGGGACCAGCGGTTGAATGGGCGCATGATGGAGGAGGTGTGGGGG
Myrtus               ------------------------------------------------------------
AB047093Vitis   1102 TGCAGGCCCTTTTTTGGGGACCAAAGGCTCAATGGGAGGATGGTGGAGGATGCTTTGGAG
AY072325Arabido 1120 TGCAGGCCATTTTTTGGGGATCAGAGATTGAACGGAAGAGCGGTGGAGGTTGTGTGGGAG
AF117267Malus   1180 TGTAGGCCATATTTTGCAGACCAGAGGCTTAATGCAAGGATGGTGGAGGAGGTGTTTGAG

XM_010064884Euc 1222 CTTGGAGTGGGAGTTGAGGGGGGTGTGTTCACCAAGCAGGCCCTGCTGAGTTGCTTGCAG
XM_010064907Euc 1165 CTTGGAGTGGGAATTGAGGGGGGTGTGTTCACCAAGAAGGCCGTGTTGAGTTGCTCGCAG
Myrtus               ------------------------------------------------------------
AB047093Vitis   1162 ATTGGAGTGAGAATTGAAGGTGGGGTTTTCACAAAGAGTGGGCTAATGAGTTGCTTTGAT
AY072325Arabido 1180 ATTGGAATGACGATTATCAATGGAGTCTTCACGAAAGATGGGTTTGAGAAGTGTTTGGAT
AF117267Malus   1240 ATCGGGGTAACCGTGGAGGATGGAGTTTTTACCAGGGAGGGGCTGGTAAAAAGCTTGGAA

XM_010064884Euc 1282 CTTCTTCTGTCCCAAGAAAGGGGGGAAATCATGAGGGACAACATGAGAGCCCTCCGACGA
XM_010064907Euc 1225 CTTCTTCTGGCGCAAGAAAGGGGGAAAATCATGAGGGACAACACGAGAACCCTCCGACAA
Myrtus               ------------------------------------------------------------
AB047093Vitis   1222 CAAATTCTCTCACAAGAAAAAGGGAAGAAACTGAGGGAAAATCTGAGAGCCCTAAGAGAG
AY072325Arabido 1240 AAAGTTTTAGTTCAAGATGATGGTAAGAAGATGAAATGTAATGCTAAGAAACTTAAAGAA
AF117267Malus   1300 GTGGTTTTGTCGCCTGAAAGTGGGAGGAAATTCAGAGACAATATAAAGAGGGTCAAACAA

XM_010064884Euc 1342 CAAGCTGAGAAGGCGGTAGGACCGGATGGGTCCTCCACTCGCAACTTTGAACTCCTCTTG
XM_010064907Euc 1285 CAAGCTGAGAAGGCGGTTGGACCGGATGGGTCCTCCACTCACAACTTCAAGCTCCTTTTG
Myrtus               ------------------------------------------------------------
AB047093Vitis   1282 ACTGCAGACAGGGCAGTTGGTCCTAAAGGGAGTTCTACTGAGAATTTCAAAACCCTGGTG
AY072325Arabido 1300 CTAGCTTACGAAGCTGTCTCTTCTAAAGGAAGGTCCTCTGAGAATTTCAGAGGATTGTTG
AF117267Malus   1360 CTGGCAGTAGAGGCGGTTGGACCACAAGGGAGCTCCACTCGGAACTTCAAATCGCTGTTG

XM_010064884Euc 1402 GACATTGTATCAAACGCCTAA------------
XM_010064907Euc 1345 GACATTGTATCAAACCCCTAG------------
Myrtus               ---------------------------------
AB047093Vitis   1342 GATTTAGTGTCAAAACCAAAGGATGTCTAG---
AY072325Arabido 1360 GATGCAGTTGTAAACATTATTTGA---------
AF117267Malus   1420 GACATCGTATCAGGATCCAATTATCAAGTATAG
```

Figure S12. Alignment of the translated amplicon-amino acid sequence from *M. communis* and the full-length UFGT sequences from *E. grandis, V. vinifera, M. domestica* and *A. thaliana*. Identical amino acids are shaded in black, and similar amino acids are shaded in grey. The remaining residues are in a white background.

```
XP_010063186Euc     1 MSSSPSDSVQCLDPPREMRTSGSGSGSPHVAVLAFPFGTHAAPLLAVIRRLASATPDTLY
XP_010063209Euc     1 -------------------MSTTGRGSPHVAVLAFPFSTHAAPLLAVIRRLATATPDTLY
Myrtus              1 ------------------------------------------------------------
BAB41020Vitis       1 --------------------MSQTITNPHVAVLAFPFSTHAAPLLAVVRRLAAAAPHAVF
AAL61932Arabido     1 -----------------MTKPSDPIRDSHVAVLAFPFGTHAAPLLTVTRRLASASPSTVF
AAD26203Malus       1 --MAAPLPIEIEPSSTNGQPHLADAYNRHVAVVAFPFTSHASALLETVRRLATALPNTLF

XP_010063186Euc    61 SFFSTADSIASVFSAYRH---LPNLRAYHVGAGVPEGHVRVGKPEEDIELFLRAAPASFR
XP_010063209Euc    42 SFYSTAKSIASIFSASNN---LPNVIGYDVGDGVPEGYVWVGKPQEDIELFLRAAPANFR
Myrtus              1 ----------------------------------------------------------FR
BAB41020Vitis      41 SFFSTSQSNASIFHDSMHTM-QCNIKSYDVSDGVPEGYVFAGRPQEDIELFMRAAPESFR
AAL61932Arabido    44 SFFNTAQSNSSLFSSGDEADRPANIRVYDIADGVPEGYVFSGRPQEAIELFLQAAPENFR
AAD26203Malus      59 SFFSTSKSNSSLFSNNSIDNMPRNIRVYDVADGVPEGYVFVGKPQEDIELFMNAAPENIR

XP_010063186Euc   118 KGMERAVAETGRRVSCLVTDAFFWFAADMAAEMELPWVAFWTAGPASLSAHLYTEHIRQT
XP_010063209Euc    99 KGMEEAVAKTGRRVTCLVTDAFFWFAADMAAKMELPWVAFWTAGPASLSVHLYTEHIRQT
Myrtus              3 KGIEEAVAKTGRGVSCLVTDAFFWFAADMAAEMELPWVAFWPAGPASLSAHLYTEHIRQT
BAB41020Vitis     100 QGMVMAVAETGRPVSCLVADAFIWFAADMAAEMGVAWLPFWTAGPNSLSTHVYTDEIREK
AAL61932Arabido   104 REIAKAETEVGTEVKCLMTDAFFWFAADMATEINASWIAFWTAGANSLSAHLYTDLIRET
AAD26203Malus     119 RSLDASVADIGKQISCLITDAFLWFGVHLADELGVPWVTFWISGLKSLSVHVHTDLIRDT

XP_010063186Euc   178 LGASLGMDGRA---DETLQFIPGMSKIRIRDLPEGVVFGNLDSLFSRMLCDMGRALPRAA
XP_010063209Euc   159 LGASRGMDGRA---DETLQFIPGMSKIRIRDLPEGVVFGNLDSLFSRMLCDMGRALPRAA
Myrtus             63 ------------------------------------------------------------
BAB41020Vitis     160 IGVSG-IQGRE---DELLNFIPGMSKVRFRDLQEGIVFGNLNSLFSRMLHRMGQVLPKAT
AAL61932Arabido   164 IGVKEVGERME---ETIG-VISGMEKIRVKDTPEGVVFGNLDSVFSKMLHQMGLALPRAT
AAD26203Malus     179 IGTQGITGRENDLIVDKNVNIQGLSNVRIKDLAEGVIFGNLDSVISGMLLQMGRLLPRAT

XP_010063186Euc   235 AVFLNSFEELDPTITADLKSKLNNFLNVGPFNLIAT--QPRAPDESKCMSWLDGQ-GRAS
XP_010063209Euc   216 AVFLNSFEELDPTITADLKSKLNNFLNVGPFNLIAT--QPRAPDESKCMSWLDGQ-GRAS
Myrtus                ------------------------------------------------------------
BAB41020Vitis     216 AVFINSFEELDDSLTNDLKSKLKTYLNIGPFNLITP--PPVIPNTTGCLQWLKER-KPTS
AAL61932Arabido   220 AVFINSFEDLDPTLTNNLRSRFKRYLNIGPLGLLSSTLQQLVQDPHGCLAWMEKR-SSGS
AAD26203Malus     239 AVFMNGFEELELPIPNDLKSKVNKLLNVGPSNVASP--LPPLPPSDACLSWLDKQQAPSS

XP_010063186Euc   292 VAYVSFGSVTVPPREEIVELAEALEASRVPFIWSLKDHLRENLPEGFLERNE--TRGMMV
XP_010063209Euc   273 VAYISFGSVTVPPREEIVELAEALEASRVPFIWSLKDHLIETLPEGFLKGTE--TRGMVV
Myrtus                ------------------------------------------------------------
BAB41020Vitis     273 VVYISFGTVTTPPPAELVALAEALEASRVPFIWSLRDKARVHLPEGFLEKTR--GYGMVV
AAL61932Arabido   279 VAYISFGTVMTPPPGELAAIAEGLESSKVPFVWSLKEKSLVQLPKGFLDRTR--EQGIVV
AAD26203Malus     297 VVYISFGTVASPAEKEQMAIAEALEATGAPFLWSIKDSCKTPLLNEFLTKTLSKLNGMVV

XP_010063186Euc   350 AWAPQEEILKHAAIGAFITHCGWNSLLESIGGGGVAMMCRPFFGDQRLNGRMMEEVWGLG
XP_010063209Euc   331 AWAPQEEILRHDAVGAFITHCGWNSLLESIGGGGVAMICRPFFGDQRLNGRMMEEVWGLG
Myrtus                ------------------------------------------------------------
BAB41020Vitis     331 PWAPQAEVLAHEAVGAFVTHCGWNSLWESVAGG-VPLICRPFFGDQRLNGRMVEDALEIG
AAL61932Arabido   337 PWAPQVELLKHEATGVFVTHCGWNSVLESVSGG-VPMICRPFFGDQRLNGRAVEVVWEIG
AAD26203Malus     357 PWAPQPHVLAHDSVGAFVSHCGWNSIMETIAGR-VPMICRPYFADQRLNARMVEEVFEIG

XP_010063186Euc   410 VGVEGGVFTKQALLSCLQLLLSQERGEIMRDNMRALRRQAEKAVGPDGSSTRNFELLLDI
XP_010063209Euc   391 VGIEGGVFTKKAVLSCSQLLLAQERGKIMRDNTRTLRQQAEKAVGPDGSSTHNFKLLLDI
Myrtus                ------------------------------------------------------------
BAB41020Vitis     390 VRIEGGVFTKSGLMSCFDQILSQEKGKKLRENLRALRETADRAVGPKGSSTENFKTLVDL
AAL61932Arabido   396 MTIINGVFTKDGFEKCLDKVLVQDDGKKMKCNAKKLKELAYEAVSSKGRSSENFRGLLDA
AAD26203Malus     416 VTVEDGVFTREGLVKSLEVVLSPESGRKFRDNIKRVKQLAVEAVGPQGSSTRNFKSLLDI
```

```
XP_010063186Euc  470 VSN-----
XP_010063209Euc  451 VSNP----
Myrtus               --------
BAB41020Vitis    450 VSKPKDV-
AAL61932Arabido  456 VVNII---
AAD26203Malus    476 VSGSNYQV
```

Figure S13. Alignment of the partial nucleotide sequence of *FLS* from *M. communis* and the full-length sequences from *E. grandis, V. vinifera, M. domestica* and *A. thaliana*. Identical nucleotides among the sequences are in a black background, whereas those that are similar among the nucleotide sequences are in grey background. The dashes indicate gaps introduced to improve alignment.

```
Myrtus               1 ------------------------------------------------------------
XM_010065055Euc      1 ATGGAAGTGGAGAGAATTCAAGCATTGGCCGCGT---TCGGCCTC---GACACGCTCCCA
AB213566Vitis        1 ATGGAGGTTGAGAGAGTGCAAGCCCTGGCCTCTGGTGCTGATCTC---AATGAGCTTCCG
AY965343Malus        1 ATGGGAGTGGAGTCTGTGGAGAGAGAACGCGAAAG---CAATGAG---GGTACAATCCCA
U84258Arabidops      1 ATGGAGGTCGAAAGAGTCCAAGACATTTCATCTTCTTCTCTACTAACAGAAGCAATCCCG

Myrtus               1 ------------------------------------------------------------
XM_010065055Euc     55 GCCCAGTTTGTCCGCCCCTCCCACGAGCGGCCAGAGAACAGCCCGGCCGTGGAGGGCGT-
AB213566Vitis       58 GCGAAATTCATCCGGCCAGCCCATGAGCGGCCGGAGAATACCAAGCCCCTTGAAGGGGT-
AY965343Malus       55 GCTGAGTTCATTAGGTCGGAGAACGAGCAACCGGGAATCACAACCGTTCATGGGAAAGTC
U84258Arabidops     61 TTGGAGTTCATCAGATCAGAGAAAGAACAACCAGCGATCACAACATTCCGAGGTCCAACG

Myrtus               1 ------------------------------------------------------------
XM_010065055Euc    114 --CACCGTGCCGGTGATCTCGCTATCCCAGCCCCACCACGCT---CTTATCCGGGAAATC
AB213566Vitis      117 --GAGCGTGCCGGTCATCTCCTTAGCGGAGTCTCACGATGTT---TTGGTGAAGGAAATC
AY965343Malus      115 TTGGAAGTACCAATAATTGATTTCAGTGACCCTGATGAGGAAAAGCTGATCGTCCAAATT
U84258Arabidops    121 CCGGCGATTCCCGTCGTCGATCTAAGCGATCCCGACGAAGAAAGCGTGAGGCGCGCGGTG

Myrtus               1 ---GAGGCGTGCTGCGAGTGGGGGTTCTTCCTCGTGACCGACCACGGGATACCGCCGGAG
XM_010065055Euc    169 CACGAGGCGTGCCGCGACTGGGGATTCTTCCTTGTGACCGATCACGGGATACCACCGGAG
AB213566Vitis      172 TACAAGGCTTGTAGCGAGTGGGGATTTTTCCTTCTGAAGGATCATGGTATATCTCCAGGG
AY965343Malus      175 ACTGAGGCCAGCTCCAACTGGGGCATGTACCAGATAGTGAACCACGACATTCCCAGCGAG
U84258Arabidops    181 GTGAAAGCGAGTGAAGAATGGGGGCTATTCCAAGTGGTTAACCACGGGATTCCGACGGAG

Myrtus              58 CTGATCGGGCGGCTGCAGGAGGCGGCCGCGAGTTCTTCAGGCTCCCGCAAGAGGAGAAG
XM_010065055Euc    229 CTGATCGGGCAGCTGCAGGAGGCGGCCGCGAGTTCTTCAGGCTCCCGCAGGAGGAGAAG
AB213566Vitis      232 TTGATAGAAAAGTTGCAGGAGGTTGGGATAGAATTCTTTAAGCAGCCACAGGAGGAGAAG
AY965343Malus      235 GTCATAAGCAAATTGCAGGCTGTTGGGAAGGAGTTTTTTGAGCTTCCACAGGAGGAAAAG
U84258Arabidops    241 CTGATACGACGTTTACAAGACGTCGGAAGAAAATTCTTCGAGCTTCCTTCGTCGGAGAAA

Myrtus             118 GAGAAGTACGCG------------------------------------------------
XM_010065055Euc    289 GAGAAGTACGCGAACGATCCGTCCAAAGGGAATTTCGAAGGGTACGGGACGAAGATGACC
AB213566Vitis      292 GAGAAGTATGCAAATGATCCTTCAACTGGTAAGTTTGAAGGGTATGGAACCAAGATGACT
AY965343Malus      295 GAGGCTTATGCTAAGCCTCCGGACTCCGGCTCAATTGAAGGCTATGGGACAAAGCTCTTC
U84258Arabidops    301 GAATCCGTCGCTAAACCGGAAGATTCGAAAGACATTGAAGGATACGGAACAAAGCTTCAG

Myrtus                 ------------------------------------------------------------
XM_010065055Euc    349 AAGAACCATGACGA------------AAAGGTTGAGTGGGTGGATTACTTTTTCCACTTC
AB213566Vitis      352 AAGAACCTTGATGA------------GAAGGTGGAGTGGGTTGATTATTTTTTCCATCTC
AY965343Malus      355 AAGGAAATTTCTGAAGGCGATACTACGAAGAAAGGGTGGGTGGACAATCTGTTCAATAAG
U84258Arabidops    361 AAAGATCCAGAAGG------------TAAAAAAGCTTGGGTCGATCATCTCTTCCATCGA

Myrtus                 ------------------------------------------------------------
XM_010065055Euc    397 ATGCATCCATCCGGCAAGGTCAGGCATGAAATCTGGCCTCAACACCCGCCTTCTTACAGG
AB213566Vitis      400 ATGTCTCCTCCTTCTAATGTTAACCATCAGATCTGGCCCCAAACCCCTTCTTCCTACAGG
AY965343Malus      415 ATTTGGCCTCCATCTGTTGTTAATTACCAGTTCTGGCCTAAGAACCCACCTTCTTACAGG
U84258Arabidops    409 ATCTGGCCACCGTCATGCGTCAATTACAGATTCTGGCCTAAGAATCCACCTGAATACAGG

Myrtus                 ------------------------------------------------------------
XM_010065055Euc    457 GAAGTCACTGAAGAATACAACAAGGAGATACTGAAAGTGACAGAGGTGCTGCTGGAGCTG
AB213566Vitis      460 GAAGTCACAGAAGTATACAACAAGGAACTACTAAAAGTAACAGATACGCTACTGGAGCTG
AY965343Malus      475 GAAGCTAATGAAGAGTATGCTAAGCATTTGCACAACGTGGTGGAAAAGCTATTTAGGCTG
U84258Arabidops    469 GAGGTGAATGAAGAGTATGCAGTGCATGTGAAGAAGCTATCGGAGACGTTATTAGGGATT

Myrtus                 ------------------------------------------------------------
XM_010065055Euc    517 ATCTCGGAGGGCCTAGGACTGGAGAAAAATGTCTTGAAAGCAAGTTTGGGAGGTGACCAG
AB213566Vitis      520 CTCTCAGAGGGGCTCGGTTTGGAAGGGAAGGTCCTGAAATCACACGTGGGTGGTGATGAA
AY965343Malus      535 CTATCTTTAGGGTTAGGGCTTGAAGGACAAGAGTTAAAGAAGGCTGCCGGTGGTGATAAC
U84258Arabidops    529 CTCTCGGATGGATTAGGGTTAAAGCGTGATGCGTTGAAAGAAGGTCTCGGCGGAGAGATG
```

```
Myrtus                 ------------------------------------------------------------
XM_010065055Euc   577 ATGGAGCTAGAGATGAAAATAAACCTGTACCCACCCTGCCCACAACCTCAGCTGGCCCTC
AB213566Vitis     580 ATTGAACTTGAAATGAAAATCAACATGTACCCACCATGCCCACAGCCTCAACTGGCGCTC
AY965343Malus     595 TTAGAGTACCTTCTCAAAATTAACTACTACCCACCATGTCCTCGCCCTGATCTTGCCCTT
U84258Arabidops   589 GCGGAGTATATGATGAAGATTAACTATTATCCGCCGTGTCCTCGGCCGGATTTAGCTTTA

Myrtus                 ------------------------------------------------------------
XM_010065055Euc   637 GGCGTCGAGCCTCACACCGACATGTCGGCAGTCACCATACTGGTCCCGAACGATGTCCCG
AB213566Vitis     640 GGAGTTGAGCCTCACACCGACATGTCCGCCCTCACCCTACTCGTGCCCAATGATGTTCCA
AY965343Malus     655 GGAGTGGTGGCTCACACCGACATGTCCACCGTCACCATTCTCGTCCCCAACGATGTTCAG
U84258Arabidops   649 GGTGTACCGGCTCATACAGATCTCAGTGGAATCACTCTTCTTGTTCCTAACGAAGTTCCT

Myrtus                 ------------------------------------------------------------
XM_010065055Euc   697 GGTCTCCAAGTTTGGAAAGACGGCAATTGGTTCGCCGCAGATTACTTGCCCGATGCTCTT
AB213566Vitis     700 GGCCTTCAAGTCTGGAAAGACGATTACTGGCTTGCCGTCGATTACTTGCCAAATGCCCTC
AY965343Malus     715 GGCCTCCAGGCTTGCAAAGATGGCCGGTGGTACGACGTTAAGTACATCCCTAATGCCCTT
U84258Arabidops   709 GGACTTCAAGTTTTCAAAGATGATCACTGGTTCGATGCAGAGTATATTCCCTCCGCCGTC

Myrtus                 ------------------------------------------------------------
XM_010065055Euc   757 TTCTGCCACGTCGGCGATCAAATAGAGGTTCTAAGCAACGGAAAGTACAAAAGTGTACTT
AB213566Vitis     760 TTTGTCCATGTCGGTGATCAAATTGAGGTGCTGAGCAATGGGAAGTACAAGAGTGTTCTC
AY965343Malus     775 GTCATCCACATTGGTGATCAGATGGAGATAATGAGCAATGGAAAGTACACGAGTGTCCTG
U84258Arabidops   769 ATTGTTCACATCGGCGATCAGATTCTGAGGTTGAGTAATGGGAGGTATAAAAATGTGTTG

Myrtus                 ------------------------------------------------------------
XM_010065055Euc   817 CATAGGAGCTTGGTGAACAAGGAAAAGACGCGAATGTCATGGGCAGTGTTTGTTGCACCC
AB213566Vitis     820 CACAGGAGTACAGTGAACAAGGAACGAACCCGCATGTCATGGGCTGTGTTTTGCGCACCT
AY965343Malus     835 CACAGAACCACTGTGAACAAGGATAAGACAAGAATCTCATGGCCTGTGTTCTTGGAGCCG
U84258Arabidops   829 CATAGGACGACGGTGGATAAAGAGAAGACGAGGATGTCGTGGCCGGTTTTCTTGGAGCCT

Myrtus                 ------------------------------------------------------------
XM_010065055Euc   877 CCACAAGAGGCACTGATCGGACCTCTCCCGGAGCTCATCAACGATCAGAACCCGGTGAAG
AB213566Vitis     880 CCCCATAAGGCAATGATCGGACCTCTTCCAGAGCTTGTGGATGAACCAAACCCTGCCAAG
AY965343Malus     895 CCAGCAGACCACGTCGTGGGTCCTCATCCACAGCTTGTGAACGCCGTTAATCAACCAAAG
U84258Arabidops   889 CCCCGTGAAAAGATTGTTGGACCTTTACCGGAACTAACCGGAGATGATAATCCTCCAAAG

Myrtus                 ------------------------------------------------------------
XM_010065055Euc   937 TACTCCACCAAGACGTTTGCCGAGTTTCGATACCGCAAATTCAACAAGCTCCCGCAGTAG
AB213566Vitis     940 TATTCAACCAAAACCTTTGCTGAGTACCGCTACCGCAAATTTAATAAGCTTCCTCAATAA
AY965343Malus     955 TACAAGACCAAGAAGTACGGCGACTATGTTTACTGTAAGATTAACAAGCTTCCCCAATAA
U84258Arabidops   949 TTTAAACCGTTTGCTTTCAAGGATTACAGTTACCGCAAGCTCAATAAACTTCCTCTGGAT

Myrtus                 ---
XM_010065055Euc   997 ---
AB213566Vitis    1000 ---
AY965343Malus    1015 ---
U84258Arabidops  1009 TGA
```

Figure S14. Alignment of the translated amplicon-amino acid sequence from *M. communis* and the full-length FLS sequences from *E. grandis, V. vinifera, M. domestica* and *A. thaliana*. Identical amino acids are shaded in black, and similar amino acids are shaded in grey. The remaining residues are in a white background.

```
Myrtus                1 ------------------------------------------------------------
XP_010063357Euc       1 ---MEVERIQALAAFGLDTLPAQFVRPSHERPENSPAVEGVTVPVISL-SQPHHALIREI
BAE75810Vitis         1 --MEVERVQALASGADLNELPAKFIRPAHERPENTKPLEGVSVPVISL-AESHDVLVKEI
AAX89401Malus         1 --MGVESVERERESNEGTIPAEFIRSENEQPGITTVHGKVLEVPIIDFSDPDEEKLIVQI
AAC69362Arabido       1 MEVERVQDISSSSLLTEAIPLEFIRSEKEQPAITTFRGPTPAIPVVDLSDPDEESVRRAV

Myrtus                1 -EACCEWGFFLVTDHGIPPELIGRLQEAGREFFRLPQEEKEKYA----------------
XP_010063357Euc      57 HEACRDWGFFLVTDHGIPPELIGQLQEAGREFFRLPQEEKEKYANDPSKGNFEGYGTKMT
BAE75810Vitis        58 YKACSEWGFFLLKDHGISPGLIEKLQEVGIEFFKQPQEEKEKYANDPSTGKFEGYGTKMT
AAX89401Malus        59 TEASSNWGMYQIVNHDIPSEVISKLQAVGKEFFELPQEEKEAYAKPPDSGSIEGYGTKLF
AAC69362Arabido      61 VKASEEWGLFQVVNHGIPTELIRRLQDVGRKFFELPSSEKESVAKPEDSKDIEGYGTKLQ

Myrtus                  ------------------------------------------------------------
XP_010063357Euc     117 K----NHDEKVEWVDYFFHFMHPSGKVRHEIWPQHPPSYREVTEEYNKEILKVTEVLLEL
BAE75810Vitis       118 K----NLDEKVEWVDYFFHLMSPPSNVNHQIWPQTPSSYREVTEVYNKELLKVTDTLLEL
AAX89401Malus       119 KEISEGDTTKKGWVDNLFNKIWPPSVVNYQFWPKNPPSYREANEEYAKHLHNVVEKLFRL
AAC69362Arabido     121 K----DPEGKKAWVDHLFHRIWPPSCVNYRFWPKNPPEYREVNEEYAVHVKKLSETLLGI

Myrtus                  ------------------------------------------------------------
XP_010063357Euc     173 ISEGLGLEKNVLKASLGGDQMELEMKINLYPPCPQPQLALGVEPHTDMSAVTILVPNDVP
BAE75810Vitis       174 LSEGLGLEGKVLKSHVGGDEIELEMKINMYPPCPQPQLALGVEPHTDMSALTLLVPNDVP
AAX89401Malus       179 LSLGLGLEGQELKKAAGGDNLEYLLKINYYPPCPRPDLALGVVAHTDMSTVTILVPNDVQ
AAC69362Arabido     177 LSDGLGLKRDALKEGLGGEMAEYMMKINYYPPCPRPDLALGVPAHTDLSGITLLVPNEVP

Myrtus                  ------------------------------------------------------------
XP_010063357Euc     233 GLQVWKDGNWFAADYLPDALFCHVGDQIEVLSNGKYKSVLHRSLVNKEKTRMSWAVFVAP
BAE75810Vitis       234 GLQVWKDDYWVAVDYLPNALFVHVGDQIEVLSNGKYKSVLHRSTVNKERTRMSWAVFCAP
AAX89401Malus       239 GLQACKDGRWYDVKYIPNALVIHIGDQMEIMSNGKYTSVLHRTTVNKDKTRISWPVFLEP
AAC69362Arabido     237 GLQVFKDDHWFDAEYIPSAVIVHIGDQILRLSNGRYKNVLHRTTVDKEKTRMSWPVFLEP

Myrtus                  ----------------------------------------
XP_010063357Euc     293 PQEALIGPLPELINDQNPVKYSTKTFAEFRYRKFNKLPQ-
BAE75810Vitis       294 PHKAMIGPLPELVDEPNPAKYSTKTFAEYRYRKFNKLPQ-
AAX89401Malus       299 PADHVVGPHPQLVNAVNQPKYKTKKYGDYVYCKINKLPQ-
AAC69362Arabido     297 PREKIVGPLPELTGDDNPPKFKPFAFKDYSYRKLNKLPLD
```

Figure S15. Alignment of the partial nucleotide sequence of *LAR* from *M. communis* and the full-length sequences from *E. grandis, V. vinifera, M. domestica* and *A. thaliana*. Identical nucleotides among the sequences are in a black background, whereas those that are similar among the nucleotide sequences are in grey background. The dashes indicate gaps introduced to improve alignment.

```
Myrtus               1 ------------------------------------------------------------
XM_010045996Euc      1 ------------ATGGCAGCGCCAACATCAGGCTCAGGCCGGGTCCTCATCGTAGGGGCG
MK726357Vitis        1 ATGACTGTTTCTCCGGTTCCTTC----GCCCAAG--GGTCGTGTCCTCATTGCCGGAGCA
DQ139836Malus        1 ATGACCGTTTCATCTTCTCTTTCTGTTGCCAAAAATGGCAGAGTCCTCATCGCCGGTGCC

Myrtus               1 ------------------------------------------------------------
XM_010045996Euc     49 ACCGGTTTCATTGGCCGTTTCATCACGGAAGCGAGCATCGCTGAGGGCCGGCCGACATAC
MK726357Vitis       55 ACCGGTTTCATTGGTCAGTTCGTGGCCACAGCAAGCCTGGATGCCCATCGACCCACCTAT
DQ139836Malus       61 ACCGGTTTCATCGGGAGGTTCGTAGCTGAAGCCAGCCTCGCAGCCGGTCAACCCACGTAT

Myrtus               1 ------------------------------------------------------------
XM_010045996Euc    109 ATCCTCGTCAGGCCCCGCTCGAGCAGCTCCTCCAAGGCGAACACCGCCGAGACTCTGGAA
MK726357Vitis      115 ATTCTCGCACGTCCAGGGCCCAGGAGTCCTTCTAAGGCCAAGATCTTCAAGGCCCTCGAG
DQ139836Malus      121 GTTCTCGTCCGGCCCGGCCCTCTGCACCCTTCTAAGGCTGACACCGTCAAGTCCTTCAAA

Myrtus               1 ------------------------------------------------------------
XM_010045996Euc    169 AAGAAAGGCGCGATAGTCATACATGGTTTGATGAATGACAGGAGTCTCATGGAGGCAATA
MK726357Vitis      175 GACAAAGGCGCCATCATCGTATACGGGTTGATAAACGAGCAGGAGGCTATGGAGAAGATA
DQ139836Malus      181 GACAAAGGCGCAATAATCTTACACGGGCTGATTTCCGATAAAACCCTAATGGAGAAAATG

Myrtus               1 ------------------------------------------------------------
XM_010045996Euc    229 CTCAAGGAGCATGAGATAGAGATCGTGATCTCGGCTGCTGGTGGGCGGACATTTTGGAT
MK726357Vitis      235 CTAAAAGAACATGAGATAGACATAGTAGTATCAACCGTGGCGGAGAGAGCATATTGGAT
DQ139836Malus      241 CTAAGAGAGCATGAAATCGAGATAGTAATATCAGCCGTGGTGGTGCCACAATATTGGAC

Myrtus               1 ------------------------------------------------------------
XM_010045996Euc    289 CAACTCACTTTGGTGGAGGCCATCAAATCTGTCGGTACCGTTAAGAGATTCTTGCCGTCA
MK726357Vitis      295 CAAATCGCCCTAGTGAAAGCCATGAAGGCTGTTGGAACCATTAAGAGATTTTTGCCGTCT
DQ139836Malus      301 CAGATCACCCTAGTCGACGCCATTGCGGCAGTTGGTACAGTTAAGAGGTTTTTGCCGTCG

Myrtus               1 ------------------------------------------------------------
XM_010045996Euc    349 GAGTTTGGGCACGACGTGGACAGGGCCGAACCGGTGGAGCCGGGTCTCACCATGTACAAG
MK726357Vitis      355 GAATTCGGGCACGATGTGAACAGAGCTGATCCAGTTGAGCCAGGGCTCAACATGTACAGA
DQ139836Malus      361 GAGTTTGGACACGATGTGGACAGGGCTGATCCGGTGGAACCAGGGCTCACCATGTATTTG

Myrtus               1 ------------------------------------------------------------
XM_010045996Euc    409 GAGAAGCGCAAGGTCAGACGGTTGATAGAAGAGCATGATCTGCCCTACACCTACATCTGT
MK726357Vitis      415 GAGAAGCGTAGGGTCCGACAATTAGTGGAGGAATCGGGCATACCCTTCACTTATATCTGC
DQ139836Malus      421 GAGAAACGCAAGGTTAGACGTTCCGTCGAGACATCTGGGGTACCCTACACATATATTTGC

Myrtus               1 ------------------------------------------------------------
XM_010045996Euc    469 TGCAACTCCATCGCCTCTTGGCCCTACTACGACAATAGACACCCCGCTGAGGTCCTCCCA
MK726357Vitis      475 TGCAACTCAATTGCTTCTTGGCCATACTACAATAACATTCACCCTTCTGAGGTTCTTCCT
DQ139836Malus      481 TGCAACTCGATTGCTTCTTGGCCCTACTTTGACAACACCCACCCTTCTGAGGTTCTCCCA

Myrtus               1 ------------------------------------------------------------
XM_010045996Euc    529 CCATTGGACCAGTTCCAAATCTACGGAGACGGGAACGTCAAAGCTTATTTTATTGCTGGG
MK726357Vitis      535 CCGACGGATTTCTTCCAGATTTACGGTGATGGCAATGTCAAAGCTTACTTTGTTGCAGGC
DQ139836Malus      541 CCGTTGGATCGGTTCCAAATCTACGGTGATGGCACCGTCAAAGCCTACTTTGTAGACGGT

Myrtus               1 ------------------------------------------------------------
XM_010045996Euc    589 ACTGACATCGGGAAGTTCACCATGATGACGGCGGATGATGACCGGACTCTGAACAAGTCA
MK726357Vitis      595 ACAGACATCGGAAAATTCACGATGAAAACAGTGGACGATGTCCGAACACTGAACAAATCA
DQ139836Malus      601 ACGGATATCGGGAAGTTCACAATGAAAACGGTGGACGACATGCGAACAATCAACAAAAAT

Myrtus               1 GTGCATTTCCGGCCGCCTTGCAACAACTGCAACATGAACGAGCTCGCGGCCTTGTGGGAG
XM_010045996Euc    649 GTGCATTTCCGGCCGCCTCGCAACAAATACAACATGAACGAGCTCGCGGCCTTGTGGGAA
MK726357Vitis      655 GTGCATTTCCGGCCATCTTGCAATTGTCTCAACATAAATGAACTCGCATCTGTGTGGGAA
DQ139836Malus      661 GTTCATTTCCGACCGCCGAGCAATTTATACGACATCAATGGGCTTGCATCCCTGTGGGAG
```

```
Myrtus              61 ACGAAGATCGGACGCACCCTCCCTCGTGTCATCATC------------------------
XM_010045996Euc    709 ACGAAGCTCGGATGCACCCTCCCTCGTGTCATCATCACCGAAGACGACCTCCTATCTGCT
MK726357Vitis      715 AAGAAGATTGGGAGGACACTTCCCAGAGTAACCGTCACTGAAGATGATCTACTAGCTGCA
DQ139836Malus      721 AAGAAAATTGGCCGGACCCTCCCTAGAGTCACCGTCACTGAAGATCACCTACTAGCCCTT

Myrtus                 ------------------------------------------------------------
XM_010045996Euc    769 GCCGCAGAGAATTGCATCCCGCAGAGCATCGTTGCCTCGTTCACTCACGACATCTTCATA
MK726357Vitis      775 GCCGGAGAAAACATCATCCCACAGAGTGTAGTAGCGGCGTTCACGCACGACATTTTCATA
DQ139836Malus      781 GCTGCAGATAACCGTATACCAGAAAGCATTGTGGCCTCGTTCACGCATGACATCTTTATC

Myrtus                 ------------------------------------------------------------
XM_010045996Euc    829 AAAGGCTGTCAAGTTAACTTCTCGGTCGACGGCCCCAACGAAGTCGAAGTGTGCAGTCTC
MK726357Vitis      835 AAGGGGTGTCAGGTGAATTTCTCCATCGATGGGCCGGAGGACGTGGAAGTGACCACCCTC
DQ139836Malus      841 AAAGGATGCCAGGTTAATTTTGCAGTGGAAGGGCCTCATGATGTGGAAGTGGGAACTCTC

Myrtus                 ------------------------------------------------------------
XM_010045996Euc    889 TACCCGGAAGAACCCTTCCGCACCGTCGACGAGTGCTTCACTGAATTCGCCGCCAAATTG
MK726357Vitis      895 TACCCTGAGGATTCTTTCAGGACGGTGGAGGAATGCTTCGGCGAATACATTGTGAAGATG
DQ139836Malus      901 TACCCTGGAGATTCTTTCAGGACCTTGGATGAGTGCTTCGATGGCTTTCTTCTCAAGTTG

Myrtus                 ------------------------------------------------------------
XM_010045996Euc    949 GGCAAGAAGGT-----------CAATCCC----------AAAGGAGAGGTCGCCGCGCAG
MK726357Vitis      955 GAGGAAAAGC---AGCCG-----ACCGCC----------GACTCTGCAATTGCCAACACC
DQ139836Malus      961 AAGGACAACCTAGAGCTAGAGCTACTGCAAGAGGAGGAGGATCAGAAAGTCTCCACCGAA

Myrtus                 ---------------------------------------------
XM_010045996Euc    988 AACCCCGTGATGGAATCCATTGCGATCGCAGCAACGTGTTCGTAA
MK726357Vitis      997 GGTCCTGTGGTTGGGATGCGGCAAGTCACTGCAACCTGCGCTTGA
DQ139836Malus     1021 AACACCGTTGTCGAATCCAGGACTGTCACTGCCACTTGTGCTTGA
```

Figure S16. Alignment of the translated amplicon-amino acid sequence from *M. communis* and the full-length LAR sequences from *E. grandis, V. vinifera, M. domestica* and *A. thaliana*. Identical amino acids are shaded in black, and similar amino acids are shaded in grey. The remaining residues are in a white background.

```
Myrtus              1 ------------------------------------------------------------
XP_010044298Eu      1 ----MAAPTSGSGRVLIVGATGFIGRFITEASIAEGRPTYILVRPRSSSSSKANTAETLE
QDH44602Vitis       1 --MTVSPVPSPKGRVLIAGATGFIGQFVATASLDAHRPTYILARPGPRSPSKAKIFKALE
AAZ79364Malus       1 MTVSSSLSVAKNGRVLIAGATGFIGRFVAEASLAAGQPTYVLVRPGPLHPSKADTVKSFK

Myrtus              1 ------------------------------------------------------------
XP_010044298Eu     57 KKGAIVIHGLMNDRSLMEAILKEHEIEIVISAAGGADILDQLTLVEAIKSVGTVKRFLPS
QDH44602Vitis      59 DKGAIIVYGLINEQEAMEKILKEHEIDIVVSTVGGESILDQIALVKAMKAVGTIKRFLPS
AAZ79364Malus      61 DKGAIILHGLISDKTLMEKMLREHEIEIVISAVGGATILDQITLVDAIAAVGTVKRFLPS

Myrtus              1 ------------------------------------------------------------
XP_010044298Eu    117 EFGHDVDRAEPVEPGLTMYKEKRKVRRLIEEHDLPYTYICCNSIASWPYYDNRHPAEVLP
QDH44602Vitis     119 EFGHDVNRADPVEPGLNMYREKRRVRQLVEESGIPFTYICCNSIASWPYYNNIHPSEVLP
AAZ79364Malus     121 EFGHDVDRADPVEPGLTMYLEKRKVRRSVETSGVPYTYICCNSIASWPYFDNTHPSEVLP

Myrtus              1 ----------------------------------------VHFRPPCNNCNMNELAALWE
XP_010044298Eu    177 PLDQFQIYGDGNVKAYFIAGTDIGKFTMMTADDDRTLNKSVHFRPPRNKYNMNELAALWE
QDH44602Vitis     179 PTDFFQIYGDGNVKAYFVAGTDIGKFTMKTVDDVRTLNKSVHFRPSCNCLNINELASVWE
AAZ79364Malus     181 PLDRFQIYGDGTVKAYFVDGTDIGKFTMKTVDDMRTINKNVHFRPPSNLYDINGLASLWE

Myrtus             21 TKIGRTLPRVII------------------------------------------------
XP_010044298Eu    237 TKLGCTLPRVIITEDDLLSAAAENCIPQSIVASFTHDIFIKGCQVNFSVDGPNEVEVCSL
QDH44602Vitis     239 KKIGRTLPRVTVTEDDLLAAAGENIIPQSVVAAFTHDIFIKGCQVNFSIDGPEDVEVTTL
AAZ79364Malus     241 KKIGRTLPRVTVTEDHLLALAADNRIPESIVASFTHDIFIKGCQVNFAVEGPHDVEVGTL

Myrtus                ------------------------------------------------------
XP_010044298Eu    297 YPEEPFRTVDECFTEFAAKLGK------KVNPKGEVAAQN-PVMESIAIAATCS
QDH44602Vitis     299 YPEDSFRTVEECFGEYIVKMEE------KQPTADSAIANTGPVVGMRQVTATCA
AAZ79364Malus     301 YPGDSFRTLDECFDGFLLKLKDNLELELLQEEEDQKVSTENTVVESRTVTATCA
```

Figure S17. Alignment of the partial nucleotide sequence of *ANR* from *M. communis* and the full-length sequences from *E. grandis, V. vinifera, M. domestica* and *A. thaliana*. Identical nucleotides among the sequences are in a black background, whereas those that are similar among the nucleotide sequences are in grey background. The dashes indicate gaps introduced to improve alignment.

```
Myrtus              1 ------------------------------------------------------------
XM_010054161Euc     1 ------ATGGCCTCAGAGATTGTAGGGAACAAGAAGTCGTGTGTCATCGGAGGGACTGGT
NM_001280956Vit     1 ---ATGGCCACCCAGCACCCCATCGGAAAGAAGACCGCATGTGTCGTCGGCGGCACCGGA
JN035300Malus       1 ---ATGGCCACCCAACAACCCATCTCAAAGAAGACGGCTTGCGTCGTCGGCGGCACCGGG
NM_104854Arabid     1 ATGGACCAGACTCTTACACACACCGGATCGAAGAAGGCTTGTGTCATTGGTGGCACGGGA

Myrtus              1 ------------------------------------------------------------
XM_010054161Euc    55 TTCGTGGCTTCTTTGCTGGTCAAGCTTTTGCTCGAGAAGGGCTATGCTGTGAACACCACC
NM_001280956Vit    58 TTTGTTGCATCTTTGCTGGTTAAGCTTTTGCTGCAGAAGGGCTATGCTGTCAACACCACT
JN035300Malus      58 TTCGTGGCGTCTCTGCTGGTCAAGCTGCTGCTCCAGAAGGGCTACGCCGTCAGAACCACC
NM_104854Arabid    61 AACTTAGCCTCTATTCTCATCAAGCATTTGCTTCAAAGTGGCTACAAAGTTAACACTACA

Myrtus              1 ------------------------------------------------------------
XM_010054161Euc   115 GTCAGGGACCCAGATAATCAAAAGAAGGTGTGCCATCTCCATGAGCTCCAGAACTTGGGT
NM_001280956Vit   118 GTCAGGGACCCTGACAATCAGAAAAAAGTCTCTCACCTCCTAGAACTACAGGAGTTGGGT
JN035300Malus     118 GTCAGAGACCCAGACAATCACAAGAAGGTCTCCCACCTCACATCACTACAAGAGTTGGGT
NM_104854Arabid   121 GTTAGAGATCCAGAAAACGAGAAGAAAATAGCTCACCTTAGGAAACTTCAAGAGCTTGGC

Myrtus              1 ------------------------------------------------------------
XM_010054161Euc   175 GACCTTAAGATTTTCAGGGCGGATCTCACCGAGGAATCGAGCTTCGATGCTCCTATAGCC
NM_001280956Vit   178 GACCTAAAAATCTTTCGAGCAGATCTAACTGACGAATTGAGCTTTGAGGCCCCTATAGCA
JN035300Malus     178 GAGCTAGAGATTTTAGCAGGAGATCTGACTGATGAAGGGAGCTTCGATGCTCCGATAGCA
NM_104854Arabid   181 GACCTGAAGATCTTCAAGGCAGATTTGACTGATGAAGACAGTTTCGAATCCTCATTCTCC

Myrtus              1 ------------------------------------------------------------
XM_010054161Euc   235 GGCTGCGACCTTGTCTTCCATCTAGCGACCCCGGTCAACTTTGCTTCCAAAGACCCTGAG
NM_001280956Vit   238 GGTTGCGACTTTGTCTTCCATGTTGCTACGCCCGTCCACTTTGCTTCTGAAGATCCAGAG
JN035300Malus     238 GGTTGTGATCTTGTTTTCCATGTTGCAACCCCTGTCAACTTTGCCTCAGAGGACCCGGAG
NM_104854Arabid   241 GGCTGTGAATACATCTTCCATGTCGCAACTCCGATCAACTTTAAATCCGAAGATCCCGAG

Myrtus              1 ------------------------------------------------------------
XM_010054161Euc   295 AATGACATGATCAAGCCGGCAATCCAGGGAGTCCTGAATGTTCTCAAAGCCTGTGCCAAA
NM_001280956Vit   298 AATGACATGATCAAGCCAGCAATTCAAGGAGTAGTGAATGTCATGAAAGCTTGTACAAGG
JN035300Malus     298 AACGACATGATCAAACCGGCAATCCAAGGGGTACTAAACGTTCTGAAATCGTGTGTGAAG
NM_104854Arabid   301 AAAGACATGATCAAGCCGGCGATACAAGGAGTGATCAATGTGTTGAAATCTTGCTTAAAA

Myrtus              1 ---------------------------------------GCCTCAGTTACAATCAACAAT
XM_010054161Euc   355 GCGAAGACAGTGAAACGTGTCGTGATGACTTCATCAGCAGCCGCAGTTTCAATCAACAAT
NM_001280956Vit   358 GCAAAATCAGTTAAACGAGTCATTTTGACATCCTCTGCAGCTGCTGTTACCATCAATCAG
JN035300Malus     358 GCCAAAACAGTTAAGCGTGTTGTTTTGACATCATCAGCTGCTACAGTTTCGATCAATACA
NM_104854Arabid   361 TCGAAATCAGTCAAGCGTGTGATCTACACATCTTCAGCTGCTGCTGTTTCCATCAACAAT

Myrtus             22 CTTCAGGAAACGGGTCTGGTCATGGATGAGAAAAACTGGACAGACCTCGAGTTCTTGACC
XM_010054161Euc   415 CTTCAGGAAACGGGCTTGGTCATGGATGAGAAAAACTGGACGGATGTCGAGTTCTTGTCC
NM_001280956Vit   418 CTTGATGGGACAGGTCTGGTTGTGGATGAGAAGAACTGGACTGATATTGAGTTCTTGACT
JN035300Malus     418 CTTGAGGGAACAGGTTTGGTCATGGACGAAAAAGATTGGAGTGATTTGGAGTTCTTGACT
NM_104854Arabid   421 CTTTCTGGAACCGGAATCGTGATGAACGAAGAAAACTGGACTGACGTTGAATTTCTCACA

Myrtus             82 TCTGCGAAGCCGCCTACCTGGGGGTACCCTGCTTCCAAGACGCTGGCAGAAAAGGCAGCG
XM_010054161Euc   475 TCTGTGAAGCCGCCTACCTGGGGGTACCCTGCTTCCAAGACGCTGGCGGAGAAGGCAGCG
NM_001280956Vit   478 TCCGCGAAGCCACCTACTTGGGGCTATCCTGCCTCCAAGACACTAGCTGAGAAAGCAGCT
JN035300Malus     478 ACTGTAAAGCCACCCACTTGGGGGTACCCTGCCTCCAAGACACTAGCTGAGAAGACAGCT
NM_104854Arabid   481 GAGGAGAAGCCTTTTAACTGGGGTTACCCAATCTCGAAGGTGTTAGCAGAAAAGACAGCT

Myrtus            142 TGGAAATTCGCGGAGGAAA-----------------------------------------
XM_010054161Euc   535 TGGAAATTCGCGGAGGAAAACAATATCAAACTTATAACTGTCATCCCATCCCTAATGGCT
NM_001280956Vit   538 TGGAAATTTGCCGAAGAAAATAACATTGATCTGATCACTGTCATCCCTACTCTGATGGCC
JN035300Malus     538 TGGAAATTCGCCGAAGAGAACAATATTGATCTCATCACTGTGATCCCTTCTCTTATGGCT
NM_104854Arabid   541 TGGGAATTTGCAAAAGAGAATAAGATCAATCTCGTTACCGTGATTCCGGCACTTATAGCC
```

```
Myrtus                   ------------------------------------------------------------
XM_010054161Euc  595 GGCCGTTCTCTCACTCTGGATGTCCCCAGCAGCGTCTGCCTCGCAATGTCTCTGCTGAGA
NM_001280956Vit  598 GGTTCCTCTCTTACTTCAGATGTCCCCAGCAGCATTGGACTTGCAATGTCCTTGATTACA
JN035300Malus    598 GGTCCTTCTCTCACTCCAGACGTCCCCAGCAGTATCGGCCTTGCCATGTCTTTAATCACA
NM_104854Arabid  601 GGAAACTCTCTCCTCTCCGATCCTCCGAGTAGCTTATCTCTCTCGATGTCTTTCATCACC

Myrtus                   ------------------------------------------------------------
XM_010054161Euc  655 GGAGACGAGTTCTTGATGAAT---GGGCTGAAAGGCATGCAAATGCTGTCGGGTTCTATT
NM_001280956Vit  658 GGGAATGAATTCCTCATAAAC---GGTATGAAGGGTATGCAGATGCTGTCAGGTTCAGTC
JN035300Malus    658 GGAAATGATTTCCTCATAAATATGGCCTTGAAAGGTATGCAAATGCTATCAGGTTCGATA
NM_104854Arabid  661 GGGAAAGAAATGCATGTGACC---GGTCTCAAGGAAATGCAGAAGCTATCTGGCTCGATC

Myrtus                   ------------------------------------------------------------
XM_010054161Euc  712 TCCATAACGCATGTTGAGGACGTCTGCCGTGCCCACATTTTCTTGGCCGAGAAGGAATCT
NM_001280956Vit  715 TCCATTGCACATGTGGAAGATGTTTGCCAGGCACATATATTTGTAGCTGAGAAAGAATCA
JN035300Malus    718 TCCATTTCACATGTGGAGGATGTCTGCCGGGCGCATATATTTTTGGCTGAGAAAGAATCT
NM_104854Arabid  718 TCGTTCGTCCACGTAGACGATTTAGCTCGTGCCCATTTGTTTCTTGCGGAGAAAGAAACT

Myrtus                   ------------------------------------------------------------
XM_010054161Euc  772 GCTTCGGGACGATACATCTGCTGTGCTGCTAACACCAGTGTTCCGGAACTCGCAAAATTC
NM_001280956Vit  775 GCTTCTGGCCGATACATCTGCTGTGCTGCCAATACCAGTGTTCCTGAGCTAGCAAAGTTC
JN035300Malus    778 GCTTCTGGTCGGTACATTTGCTGTGCTGCCAACACCAGCGTTCCTGAGCTTGCTCGGTTC
NM_104854Arabid  778 GCTTCTGGTCGCTACATTTGCTGTGCTTACAACACAAGTGTTCCAGAGATTGCGGATTTT

Myrtus                   ------------------------------------------------------------
XM_010054161Euc  832 CTTAACAAAAGATACCCACAATACAAATCCCCAACAGAATTCGGGGATTTCCCATCTAAG
NM_001280956Vit  835 CTGAGCAAAAGATACCCTCAGTACAAAGTCCCAACTGATTTTGGAGACTTCCCCCCTAAA
JN035300Malus    838 CTCAACAAAAGATACCCCCAGTACAAAGTCCCCACTGAGTTTGGAGATTTTCCGTCCAAG
NM_104854Arabid  838 CTCATACAGAGATATCCTAAGTACAATGTGTTGTCAGAATTCGAAGAGGGCTTGTCGATT

Myrtus                   ------------------------------------------------------------
XM_010054161Euc  892 GCCAAGTTGATCATCTCTTCAGAGAAGCTTATCAAGGAGGGCTTCACCTACAAGTACAGC
NM_001280956Vit  895 TCGAAGTTGATAATCTCCTCAGAGAAGCTTGTGAAAGAGGGGTTCAGTTTTAAGTACGGG
JN035300Malus    898 GCCAAATTGATCATCTCTTCGGAGAAGCTTATCAAGGAGGGGTTCGATTTTAAGTATGGC
NM_104854Arabid  898 CCGAAATTGACACTATCTTCGCAAAAACTTATCAATGAAGGCTTTCGATTCGAATATGGG

Myrtus                   ------------------------------------------------------------
XM_010054161Euc  952 ATTGAGGATATCTATGACCAATCTGTCGAATACTTCAAGGAAAAGGGACTGTTGCAGGTG
NM_001280956Vit  955 ATTGAAGAAATTTATGATGAAAGTGTGGAGTATTTCAAGGCCAAGGGGCTATTGCAGAAT
JN035300Malus    958 ATCGAAGAAATATATGACCAAACTGTGGAGTACTTCAAGGCCAAGGGGTTGCTGCAGAAC
NM_104854Arabid  958 ATCAATGAGATGTATGATCAAATGATAGAGTACTTCGAGTCAAAAGGATTGAT-CAAAGC

Myrtus                   -------
XM_010054161Euc 1012 TAG----
NM_001280956Vit 1015 TGA----
JN035300Malus   1018 TAG----
NM_104854Arabid 1017 TAAATAA
```

Figure S18. Alignment of the translated amplicon-amino acid sequence from *M. communis* and the full-length ANR sequences from *E. grandis, V. vinifera, M. domestica* and *A. thaliana*. Identical amino acids are shaded in black, and similar amino acids are shaded in grey. The remaining residues are in a white background.

```
Myrtus             1 ------------------------------------------------------------
XP_010052463Euc    1 --MASEIVGNKKSCVIGGTGFVASLLVKLLLEKGYAVNTTVRDPDNQKKVCHLHELQNLG
NP_001267885Vit    1 -MATQHPIGKKTACVVGGTGFVASLLVKLLLQKGYAVNTTVRDPDNQKKVSHLLELQELG
AEL79860Malus      1 -MATQQPISKKTACVVGGTGFVASLLVKLLLQKGYAVRTTVRDPDNHKKVSHLTSLQELG
NP_176365Arabid    1 MDQTLTHTGSKKACVIGGTGNLASILIKHLLQSGYKVNTTVRDPENEKKIAHLRKLQELG

Myrtus             1 ------------------------------------------------------------
XP_010052463Euc   59 DLKIFRADLTEESSFDAPIAGCDLVFHLATPVNFASKDPENDMIKPAIQGVLNVLKACAK
NP_001267885Vit   60 DLKIFRADLTDELSFEAPIAGCDFVFHVATPVHFASEDPENDMIKPAIQGVVNVMKACTR
AEL79860Malus     60 ELEILAGDLTDEGSFDAPIAGCDLVFHVATPVNFASEDPENDMIKPAIQGVLNVLKSCVK
NP_176365Arabid   61 DLKIFKADLTDEDSFESSFSGCEYIFHVATPINFKSEDPEKDMIKPAIQGVINVLKSCLK

Myrtus             1 -------------ASVTINNLQETGLVMDEKNWTDLEFLTSAKPPTWGYPASKTLAEKAA
XP_010052463Euc  119 AKTVKRVVMTSSAAAVSINNLQETGLVMDEKNWTDVEFLSSVKPPTWGYPASKTLAEKAA
NP_001267885Vit  120 AKSVKRVILTSSAAAVTINQLDGTGLVVDEKNWTDIEFLTSAKPPTWGYPASKTLAEKAA
AEL79860Malus    120 AKTVKRVVLTSSAATVSINTLEGTGLVMDEKDWSDLEFLTTVKPPTWGYPASKTLAEKTA
NP_176365Arabid  121 SKSVKRVIYTSSAAAVSINNLSGTGIVMNEENWTDVEFLTEEKPFNWGYPISKVLAEKTA

Myrtus            48 WKFAEE------------------------------------------------------
XP_010052463Euc  179 WKFAEENNIKLITVIPSLMAGRSLTLDVPSSVCLAMSLLRGDEFLMNGLKG-MQMLSGSI
NP_001267885Vit  180 WKFAEENNIDLITVIPTLMAGSSLTSDVPSSIGLAMSLITGNEFLINGMKG-MQMLSGSV
AEL79860Malus    180 WKFAEENNIDLITVIPSLMAGPSLTPDVPSSIGLAMSLITGNDFLINMALKGMQMLSGSI
NP_176365Arabid  181 WEFAKENKINLVTVIPALIAGNSLLSDPPSSLSLSMSFITGKEMHVT-GLKEMQKLSGSI

Myrtus               ------------------------------------------------------------
XP_010052463Euc  238 SITHVEDVCRAHIFLAEKESASGRYICCAANTSVPELAKFLNKRYPQYKSPTEFGDFPSK
NP_001267885Vit  239 SIAHVEDVCQAHIFVAEKESASGRYICCAANTSVPELAKFLSKRYPQYKVPTDFGDFPPK
AEL79860Malus    240 SISHVEDVCRAHIFLAEKESASGRYICCAANTSVPELARFLNKRYPQYKVPTEFGDFPSK
NP_176365Arabid  240 SFVHVDDLARAHLFLAEKETASGRYICCAYNTSVPEIADFLIQRYPKYNVLSEFEEGLSI

Myrtus               -----------------------------------------
XP_010052463Euc  298 AKLIISSEKLIKEGFTYKYSIEDIYDQSVEYFKEKGLLQV-
NP_001267885Vit  299 SKLIISSEKLVKEGFSFKYGIEEIYDESVEYFKAKGLLQN-
AEL79860Malus    300 AKLIISSEKLIKEGFDFKYGIEEIYDQTVEYFKAKGLLQN-
NP_176365Arabid  300 PKLTLSSQKLINEGFRFEYGINEMYDQMIEYFESKGLIKAK
```
